# Solvent Controlled Generation of Spin Active Polarons in Two-Dimensional Material under UV Light Irradiation

**DOI:** 10.1021/jacs.3c13296

**Published:** 2024-05-02

**Authors:** Giorgio Zoppellaro, Miroslav Medveď, Vítězslav Hrubý, Radek Zbořil, Michal Otyepka, Petr Lazar

**Affiliations:** † Regional Centre of Advanced Technologies and Materials, The Czech Advanced Technology and Research Institute (CATRIN), Palacký University Olomouc, Šlechtitelů 27, Olomouc 779 00, Czech Republic; ‡ Nanotechnology Centre, Centre for Energy and Environmental Technologies (CEET), VŠB—Technical University of Ostrava, 17. listopadu 2172/15, Ostrava-Poruba 708 00, Czech Republic; § Department of Chemistry, Faculty of Natural Sciences, Matej Bel University, Tajovského 40, Banská Bystrica 974 01, Slovak Republic; ∥ Department of Physical Chemistry, Faculty of Science, Palacký University Olomouc, 17. listopadu 12, Olomouc 771 46, Czech Republic; ⊥ IT4Innovations, VŠB − Technical University of Ostrava, 17. listopadu 2172/15, Ostrava-Poruba 708 00, Czech Republic

## Abstract

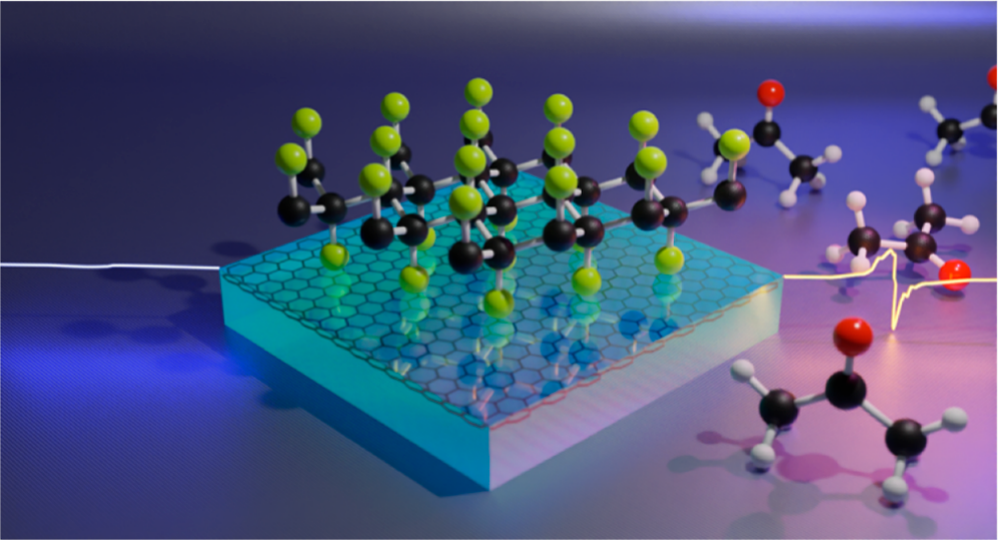

Polarons belong to a class of extensively studied quasiparticles that have found applications spanning diverse fields, including charge transport, colossal magnetoresistance, thermoelectricity, (multi)ferroism, optoelectronics, and photovoltaics. It is notable, though, that their interaction with the local environment has been overlooked so far. We report an unexpected phenomenon of the solvent-induced generation of polaronic spin active states in a two-dimensional (2D) material fluorographene under UV light. Furthermore, we present compelling evidence of the solvent-specific nature of this phenomenon. The generation of spin-active states is robust in acetone, moderate in benzene, and absent in cyclohexane. Continuous wave X-band electron paramagnetic resonance (EPR) spectroscopy experiments revealed a massive increase in the EPR signal for fluorographene dispersed in acetone under UV-light irradiation, while the system did not show any significant signal under dark conditions and without the solvent. The patterns appeared due to the generation of transient magnetic photoexcited states of polaronic character, which encompassed the net 1/2 spin moment detectable by EPR. Advanced ab initio calculations disclosed that polarons are plausibly formed at radical sites in fluorographene which interact strongly with acetone molecules in their vicinity. Additionally, we present a comprehensive scenario for multiplication of polaronic spin active species, highlighting the pivotal role of the photoinduced charge transfer from the solvent to the electrophilic radical centers in fluorographene. We believe that the solvent-tunable polaron formation with the use of UV light and an easily accessible 2D nanomaterial opens up a wide range of future applications, ranging from molecular sensing to magneto-optical devices.

## Introduction

Polarons are composite quasiparticles that can form in polarizable materials and have a profound impact on material properties and functionalities. The term polaron refers to an excess charge carrier (electron or hole) localized within a potential well generated by a displacement of surrounding ions. In a quasiparticle picture, it is a quasiparticle consisting of an electron or a hole dressed by a cloud of virtual phonons, which follows the polaron as it propagates through the crystal. A polaron may result from the separation of excitons, which are charge-neutral Coulomb-bound electron–hole pairs. In this context, polarons and excitons play a central role in the electronic and optical properties of organic semiconducting polymers^1^ and are crucial to understanding the operation of modern organic optoelectronic devices such as solar cells and light-emitting diodes.^2^ Besides that, polarons were found to stand behind the colossal magnetoresistance of manganese perovskites,^3^ ferroelectricity of lead halide perovskites,^4^ surface reactivity of TiO_2_,^5^ or the Seebeck effect in oxide-based resistive switching memory,^6^ to name just the most relevant examples. A recent review has summarized almost 90 years of research into polaron effects in materials, comprising an enormous amount of data.^7^

However, the physics of polarons and other quasiparticles such as excitons has been studied mostly in bulk crystals and on clean surfaces in a vacuum. In contrast to bulk materials, experimental evidence of polarons in two-dimensional (2D) materials is rare.^8^ Owing to this, our fundamental understanding of quasiparticles at solid–gas and solid–liquid interfaces has not yet been developed^9^ despite the fact that low dimensional nanomaterials, including also 2D nanomaterials like graphene, have large surface area exposed to the external environment, which must affect the energetics and behavior of polarons in these materials. Optical excitation near the interface can result not only in the formation of excitons but also in the creation of a wealth of other quasiparticles such as biexcitons, polaron pairs, and trions. Biexcitons are created from two free excitons. A polaron pair is a Coulomb-bound pair of a negative and a positive polaron, situated on opposite sides of the interface or on different molecules.^1,10^ Polaron pairs are sometimes also called charge-transfer excitons. A (negative) trion is an electron–hole–electron charged quasiparticle that features a net half-integer spin. The unexpected role of an interface is supported by recent studies which have revealed a surprisingly strong interaction between polarons and surface adsorbate molecules.^11,12^ Namely, polarons significantly modulated adsorption and dissociation of water on the rutile (110) surface,^11^ and, in turn, CO adsorbates were able to alter the stability of polarons in rutile.^12^ Coupling of polarons to external molecules could be crucial in 2D materials and nanomaterials because their surfaces are exposed to interaction with the environment.^13^ It is worth noting that in chemistry, we can find an analogue to a polaron—a solvated electron.^14^ A solvated electron is a carrier of negative charge in solution and is one of the most fundamental chemical reagents. The introduction of excess electrons strongly perturbs the structure of molecules within a screening volume of the liquid solution in a manner similar to how the polaron perturbs the crystalline lattice. The analogy continues as further knowledge is being acquired regarding the properties of solvated electrons in homogeneous media; however, much less is known about their behavior in inhomogeneous media such as liquid/solid and liquid/vacuum interfaces.^15^ All of these facts motivated us to study the interaction of a 2D material with a solvent.

We report massive generation of spin active states in a fluorographene (FG) frozen matrix solution under UV light triggered by specific solvents, i.e., by the specific environment surrounding the polarons in FG. We used continuous wave (CW) X-band electron paramagnetic resonance (EPR) spectroscopy to determine the character of the generated spin active species and to follow the time evolution under various conditions. The experimental observations were elucidated with the help of theoretical calculations based on the density functional theory (DFT) using both periodic and finite-size models including an explicit solvent molecule. The finite models were studied by time-dependent DFT (TD-DFT) using the ωB97X-D and CAM-B3LYP functionals in combination with the universal implicit solvation model based on the solute electron density (SMD) in order to account for the dielectric nature of the solvent environment. The periodic model was used to study the polaron formation and electron excitation in the presence of solvent molecules. For the latter, the TD-DFT and the Green function (GW) approach combined with the Bethe–Salpeter equation (BSE) were used.^16^ This combined approach allowed us to compute one-particle as well as two-particle electronic excitations in an accurate manner from the first-principles, correctly capture band alignment at the molecule–surface interface, and, at the same time, correctly account for the excitonic and local-field effects.

## Results and Discussion

**Figure 1:**
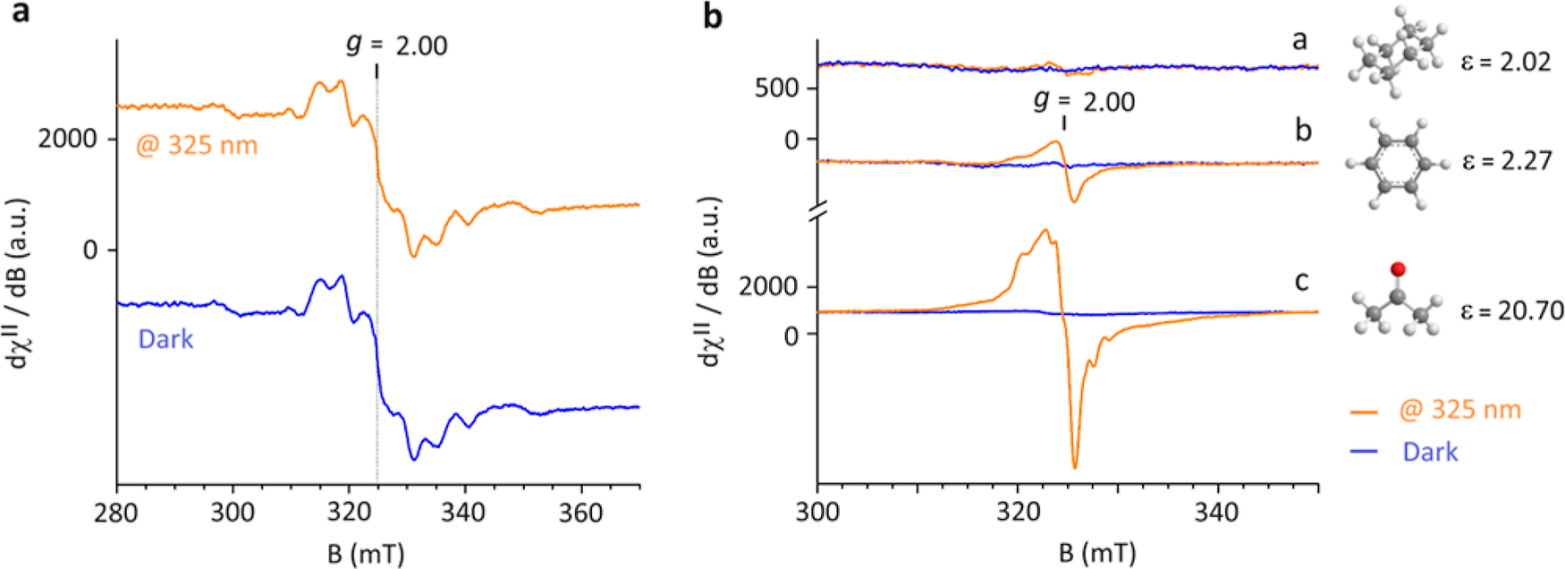
X-band EPR spectra recorded at *T* = 90 K: Panel (a) The spectrum of FG powder under dark conditions (lower spectrum, 0.6 mW of applied microwave power) compared to that obtained after 10 min of in situ continuous UV light irradiation (upper spectrum, at 325 nm, 200 mW). Panel (b) The spectra of FG powder freshly dispersed in (a) cyclohexane, (b) benzene, and (c) acetone, recorded after 10 min of in situ UV irradiation at 325 nm (orange) and in dark conditions (blue). Experimental conditions: (a) 9.077 GHz frequency, 1.6 mW microwave power; (b) 9.080 GHz frequency, 1.0 mW microwave power; (c) 9.088 GHz frequency, 0.6 mW microwave power. The solvents’ respective dielectric constants are shown next to the recorded EPR traces (a–c).

FG is a wide-gap 2D semiconductor derived from graphene,^17^ which holds a great promise for applications in high performance materials such as batteries, dielectrics, or biosensors.^18^ FG exhibits unusually high exciton binding energy of about 2 eV owing to the strongly bound Frenkel exciton.^19^ We measured CW-EPR signals under light irradiation at 3.8149 eV, which was below the optical band gap of pristine FG (∼5.6 eV).^20^Figure 1a shows the experimental EPR signal (*T* = 90 K) of the neat FG powder material obtained from a commercial source (μm size sheets, Sigma-Aldrich, graphite fluorinated polymer, (CF_*x*_)_*n*_, *x* ∼ 1.1) recorded under dark conditions, compared to the resonance signal detected during continuous UV irradiation (Figure 1a, upper spectrum). The detailed chemical and spectroscopic characterization of the commercial FG material used throughout this study is given in the Supporting Information (Figures S1–S4). The observed EPR resonances shown in Figure 1a highlight the presence of a complex pattern that developed nearly symmetrically around *g* ∼ 2.000. Such a pattern indicates the coexistence of *S* = 1/2 and *S* = 1 states. The spin content was estimated as 10.03 × 10^18^ spin/g, using CuSO_4_·5H_2_O spin *S* = 1/2 standard. Similar EPR features and comparable spin density (23.2 × 10^18^ spin/g) have been earlier observed to emerge in a different commercial batch of FG from the same commercial source (graphite fluorinated polymer, Sigma-Aldrich, CF_*x*_, with *x* ∼ 1.1) and have been interpreted to arise from the presence of spin active defects in the 2D lattice of FG. Therefore, the concentration of spin defects could slightly vary in different FG batches, but the presence of admixture of *S* = 1/2 and *S* = 1 states in bulk solids appears to be a well-retained property for samples coming from the same supplier. From the experimental EPR resonances shown in Figure 1a, it was confirmed that in the powder form there were no significant differences between the EPR spectra of the FG recorded under dark conditions and under UV light irradiation.

When the FG powder was freshly dispersed in cyclohexane (∼1 mg/mL), and the EPR spectrum of its frozen matrix solution was recorded at 90 K, an extremely weak signal was detected due to a large diamagnetic dilution (Figure 1b, EPR trace a) compared to that of the powder material in which clear resonances were seen. In situ irradiation of the cyclohexane frozen solution did not lead to the formation of additional EPR resonances or any increase in the EPR signal.

The EPR patterns changed dramatically with FG freshly dispersed in benzene and acetone (Figure 1b, EPR traces b,c) (∼1 mg/mL) and recorded under UV-light irradiation (10 min irradiation-time inside the cavity resonator before signal acquisition). While neither system showed any significant EPR signals in the frozen matrix suspensions (at *T* = 90 K) in dark conditions, upon irradiation, a strong asymmetric resonance signal was detected in the acetone solution and a weaker one was still clearly discernible in benzene. These signals were attributed to the generation of transient spin active photoexcited states. In acetone, the evaluated spin concentration for FG reached a value of 30.00 × 10^18^ spin/g, which was about 3 times higher than for the material measured under dark condition. Upon thawing the samples and then recooling down to 90 K, no leftover residual paramagnetic signals were detected under dark conditions in both the benzene and acetone solutions, implying that the paramagnetic transient species did not remain in the FG solution without irradiation. It must be emphasized that blank samples containing only the acetone or benzene solvent placed inside the EPR tubes did not show the emergence of any of such EPR features after exposure to UV light at 325 nm and at *T* = 90 K for up to 30 min. Similar results were obtained for the oxygen-free solutions. Therefore, all the following experiments for the FG system dispersed either in benzene or acetone solvents were conducted in oxygen-free and nitrogen-saturated suspensions in order to obtain the best possible spectral resolutions and to minimize paramagnetic broadening from dissolved oxygen on the detected photoexcited spin species.

**Figure 2:**
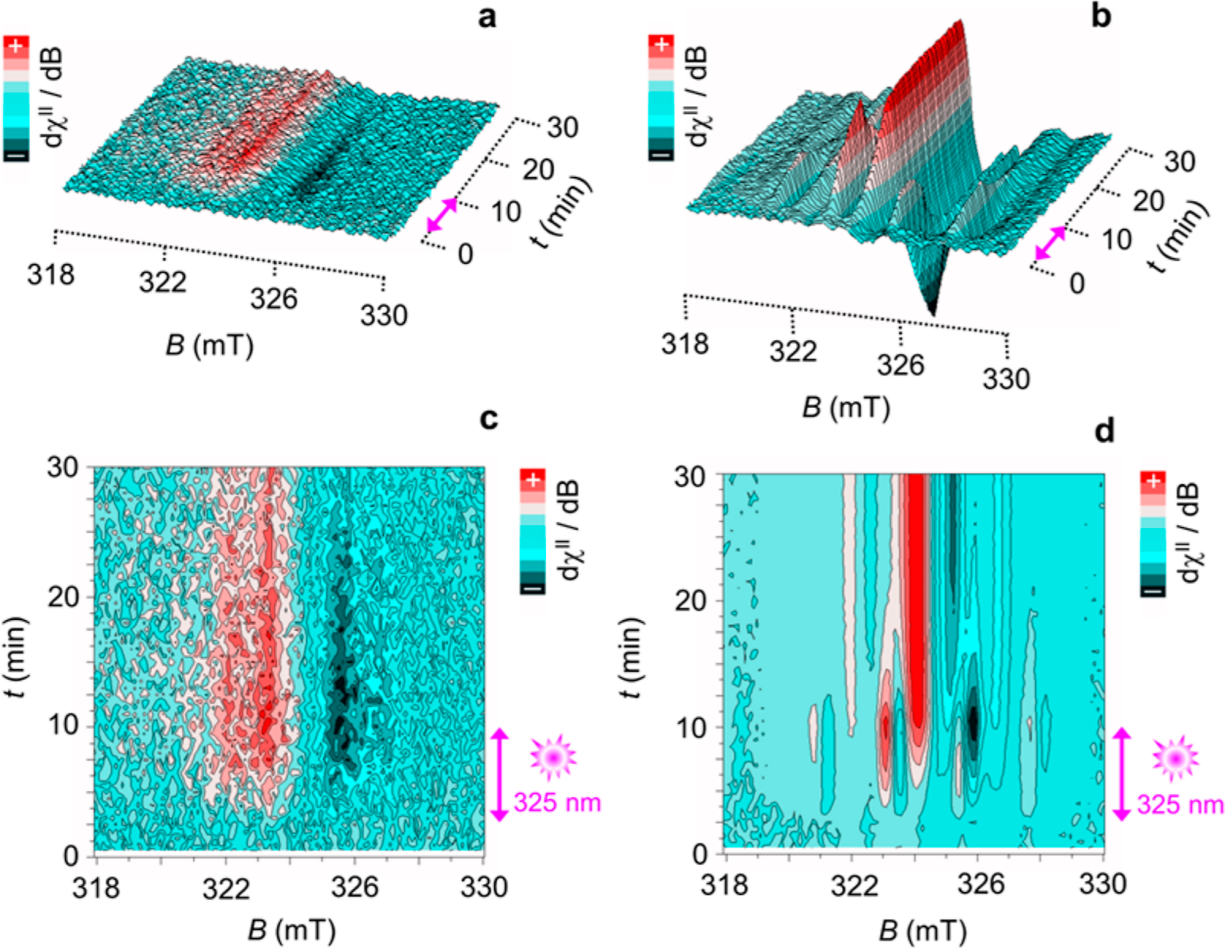
(a) In situ light-induced X-band EPR spectra (LEPR) of FG powder (C_1_F_1.1_) freshly dispersed in an oxygen free benzene solution. The sample was kept in a nitrogen saturated atmosphere. Experimental parameters: 9.081 GHz frequency, 1.0 mW microwave power, 100 kHz modulation frequency, 0.5 mT modulation width, and 30 s acquisition time for each sequential spectrum. Signal acquisition sequence: 2.5 min under dark conditions, followed by 7.5 min under light irradiation (at 325 nm), then 20 min under dark conditions. (c) The correspondent 2D LEPR plot of the spectra shown in panel (a). (b) LEPR spectra of FG powder (C_1_F_1.1_) freshly dispersed in oxygen free acetone solution. Experimental parameters: 9.075 GHz frequency, 1.0 mW microwave power, 100 kHz modulation frequency, 0.5 mT modulation width, and 30 s acquisition time for each sequential spectrum. Signal acquisition sequence: 2.5 min under dark conditions, followed by 7.5 min under light irradiation (at 325 nm), then 20 min under dark conditions. (d) The correspondent 2D LEPR plot of the spectra shown in panel (b).

The photoexcitation dynamics was examined by in situ CW-LEPR experiments using a light-off to light-on irradiation sequence at intervals of 30 s in the fast scan mode within the time window of 1800 s. To observe if any spin relaxation occurred at 90 K, a light-off to light-on irradiation sequence was employed for the in situ signal detection; 2.5 min under dark conditions, followed by 7.5 min under light irradiation and then 20 min under dark conditions. The results obtained in these dynamic light-induced EPR experiments are shown in Figure 2a,c for the FG in benzene and in Figure 2b,d for the FG in the acetone solution. The emergence of resonance signals indicated that as soon as the UV light was switched on, new paramagnetic species were clearly generated in the systems. Specific EPR features depended on the type of employed solvent. While in benzene, the weak and slightly anisotropic signal started to decay slowly as soon as the UV light was cut off, the most prominent resonance signature of FG dispersed in acetone was more complex, and the EPR signal did not decay within the experimental time window employed (20 min dark conditions). Moreover, the signal emerging under photoexcitation was significantly richer in hyperfine components (either from ^1^H or ^19^F, or from both) and underwent a significant reorganization during the light-on to light-off time window, indicating that in the FG-acetone system, there was a change in the spatial configuration of the acetone molecules that were interacting with the FG sheets. We assume that this change was not caused by the diffusion of acetone molecules because the solid-state system had limited thermal mobility at 90 K. Presumably, rotational freedom of the methyl groups of the acetone with respect to the FG plane resulted in the observed differences in the strength, type, and number of effective hyperfine components coupled to the electron spin moment when the light was cut off. Besides that, the electric dipole-driven reorientation of acetone molecules surrounding the polarons may have played its role as well. In both systems—FG in the benzene and acetone solution—the spin systems can be interpreted as bound polaron states (h^+^/e^–^), which are characterized by the formation of *S* = 1/2 species. From the overall shape of the resonance transition observed in the frozen matrix, the generation of populated triplet states under photoexcitation can be excluded at this temperature. To further generalize that the presented observations hold also to FG systems having smaller dimension (nanometer size sheets), we probed the existence of the phenomenon in FG nanosheets obtained by sonication of the commercial FG material in acetone, in order to produce small FG fragments without inducing defluorination processes. Synthetic details and characterization of FG nanoflakes are given in the Supporting Information. In particular, the EPR spectrum of small FG sheets recorded under light irradiation (Figures S5 and S6) showed that the feature of the photoexcited e^–^/h^+^ spin state (*S* = 1/2) formed in acetone remained similar in shape and resonance position to that observed in the large FG sheets recorded in commercial sample (Aldrich). However, in the small FG sheets, it was also noted that in the photoexcited EPR spectra, a stronger resonance signal around *g* ∼ 2.00 emerged, which was associated with firmly localized spins on the carbon backbone of FG. This effect was, therefore, size dependent and indicated that in the small FG sheets there are *S* = 1/2 sites that have limited coupling with the solvent molecules under light irradiation. However, the photoexcited LEPR spectra in both large and small FG sheets remained consistent with the formation of small “Holstein-type” polarons (localized systems).

**Figure 3:**
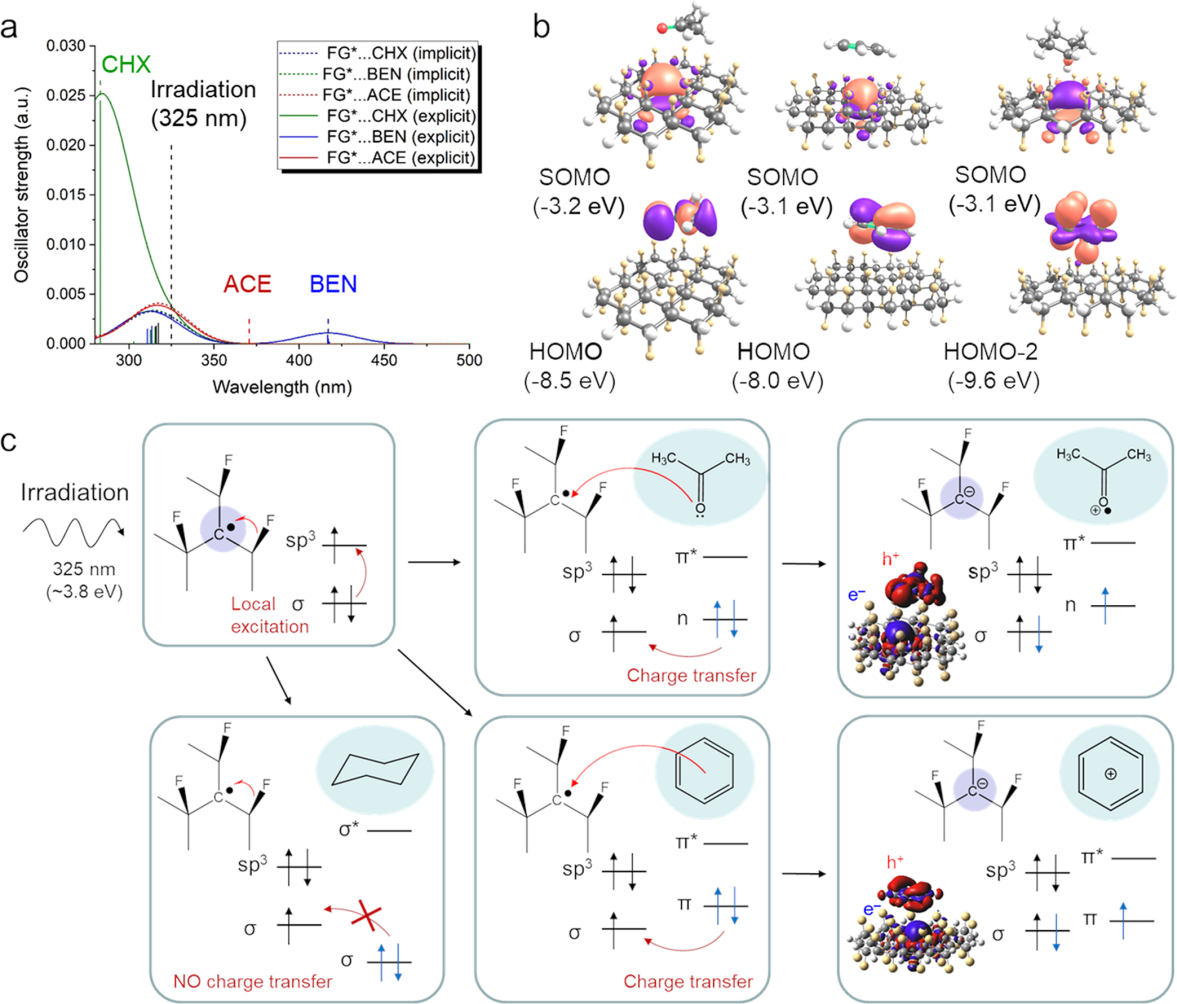
(a) Absorption spectra of defected FG in cyclohexane (CHX, green line), benzene (BEN, blue line), and acetone (ACE, red line) solutions computed without (short-dashed lines) and with (solid lines) an explicit solvent molecule in the vicinity of a radical defect. Black vertical dashed line denotes the wavelength of the applied UV light. Green, blue, and red vertical dashed lines denote the positions of the charge transfer (CT, S_0_ → S_1_) state in CHX, BEN, and ACE, respectively, (b) HOMO and lowest unoccupied molecular orbital (i.e., SOMO) of the solvated system in acetone, benzene, and cyclohexane (from left to right) corroborating the CT character of transitions shown in (panel a). (c) Proposed mechanism of the initial phase of the photoactivation of the FG-solvent interface.

The substantial impact of a solvent on the behavior of frozen FG solutions under UV-light radiation observed in the EPR experiments can be rationalized in terms of an intricate interplay of the excited states at the FG-solvent interface arising from a specific arrangement of electronic levels of FG defects and the solvent molecules. Pristine nondefective FG started to absorb radiation in the spectral region below 220 nm (∼5.6 eV),^20^ and its absorption was only mildly dependent on the solvent polarity (Figure S7). Although the interaction of pristine FG with an explicit acetone molecule gave rise to a new absorption peak corresponding to the S_0_ → S_2_ (*n*(ACE) → σ*(FG)) transition, the excitation energy (∼6.1 eV, Figure S8) was also significantly higher than that of the applied irradiation (3.8 eV). It should be noted that the S_0_ → S_1_ transition in pristine FG/acetone predominantly corresponds to local n → π* excitation of acetone and this character of the S_0_ → S_1_ transition was also corroborated by the amplitudes of the BSE eigenvectors obtained from the BSE@GW0 calculation of the periodic FG model. However, as already mentioned, the real samples of FG contained different types of defects including radical centers,^21^ which may have played an important role in the FG photoactivity. Indeed, our TD-DFT calculations show that the sole defective FG exhibited a weak absorption at ∼315 nm (3.9–4.0 eV) corresponding to the σ(C–F) → SOMO transition, which fitted the region of applied UV irradiation (Figure 3a). The position and the intensity of the peak depend only slightly on the solvent polarity, so this transition can hardly explain the dissimilar performance of FG under UV light in different environments. However, the computations for models involving an explicit solvent molecule in the vicinity of the radical defect revealed the emergence of charge transfer (CT) excited states (Figure 3b) with the transition energies (and intensities) being highly dependent on the electronic structure of the solvent. While the highest occupied molecular orbital (HOMO) energies of acetone and benzene made the CT state accessible via internal conversion of the local excitation (LE) at the FG defect in these solvents, the low-lying σ orbitals (e.g., HOMO–2) of cyclohexane caused that the CT state laid energetically above the LE state and thus was not reachable under the conditions used in the experiments. The CT states formed in acetone and benzene eventually relaxed to the ground state, but the very low oscillator strengths (Figure 3a), especially in the case of acetone, suggest that the radiative de-excitation was not probable, and the nonradiative pathways were hampered in the frozen state by low temperature and lattice confinement, dissimilar to liquid solutions. Although the proposed mechanism clearly distinguishes between the photoactive and inert solvents, it should be underlined that it only describes the first phase of the FG photoactivation and does not explain the proliferation of spin-active species. In fact, the electron transfer from acetone to SOMO localized in the FG defect site leads to the formation of a spin-inactive excess-electron site. However, as revealed by our DFT calculations, in the vicinity of such an anionic site, some C–F become susceptible toward the release of F^–^ anions (e.g., the C–F bond lengths next to a defect increased from 1.39 to1.45 Å upon the formation of an anion). The activation barrier for such a process was estimated to ca. 13.3 kcal/mol (Figure S9). While the release of a neighboring F^–^ preserves the closed-shell character of the system (path A in Figure S10), it can be anticipated that other sites (e.g., wrinkles, buckles, and edges) can be prone to the C–F bond cleavage as well in real FG samples, and such cleavage could lead to creation of new spin-active sites. Although the thorough exploration of such processes would be computationally highly demanding, as an example, we investigated an FG model containing a buckle nearby the anionic site (Figure S10). We found that for finite-size models the triplet structure (B) was only by 0.29 eV energetically less favorable than A. According to periodic model calculations, the structure B was even more stable than A (by ca. 0.31 eV, Figure S11). Let us note that the differences between the finite-size and periodic structures can arise from the larger flexibility of the former. Our calculations thus indicate that the formation of separated spin species (appearing as *S* = 1/2 species for more distant defects) might be an energetically feasible process. This could explain the multiplication of spin-active species as well as their backbone nature, as observed in EPR experiments. The generation of three spin-active species (one on an acetone molecule and two on the FG backbone) from a single radical site is also in line with the observed approximately three times higher spin concentration in irradiated FG in acetone compared to that measured under dark conditions. In addition, it can be anticipated that the polaron pairs formed at the interface may have split due to screening of the extra charge by the solvent. Such splitting may have allowed the holes to migrate into the solvent bulk (and/or to the FG-solvent interface), especially in highly polar acetone, which indeed featured much longer lifetimes of radical species than did the FG-benzene system. Whether or not the dissociation of an exciton to an electron–hole pair across the material interface results in free charges depends strongly on the dielectric constants of molecular solvents.^22^ The dielectric constant of acetone (ε = 20.70) is much higher than that of benzene (2.27) and hexane (2.02). The splitting of polaron pairs into free charges could thus explain the strong multiplication of polaron states in FG/acetone under continuous UV irradiation.

**Figure 4:**
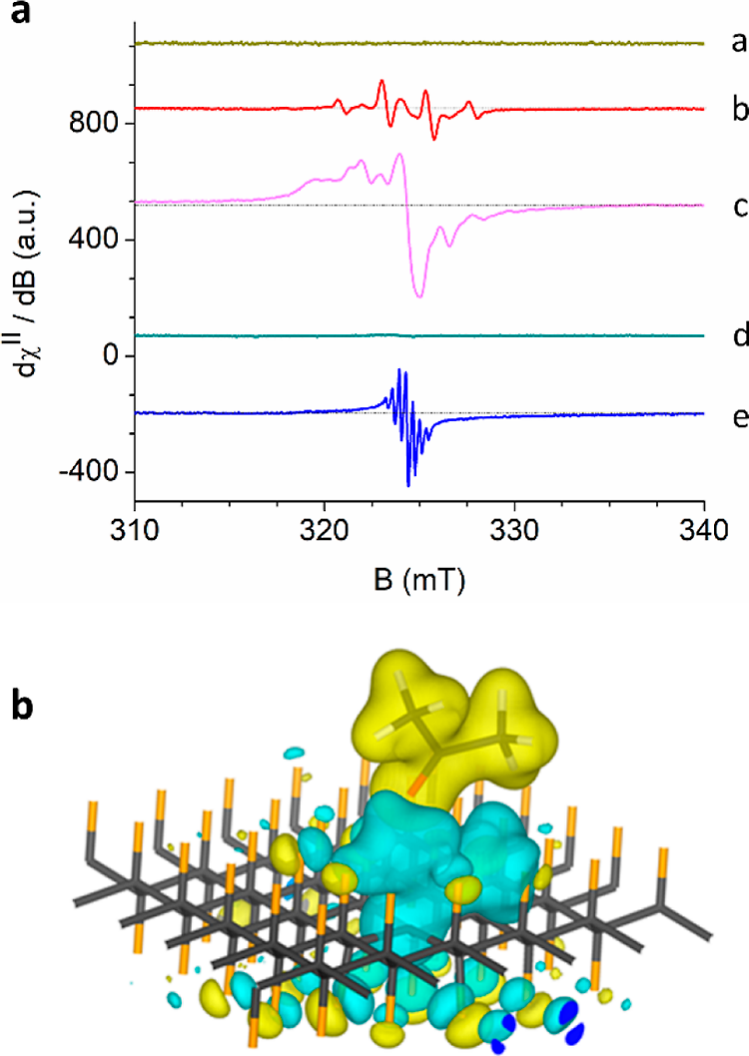
(a) X-band EPR spectra of FG powder freshly dispersed in oxygen-free acetone solvent and recorded at *T* = 90 K under dark conditions (trace a), FG/acetone recorded after 3 min under UV irradiation (@325 nm) (trace b), FG/acetone recorded after 7.5 min under UV irradiation followed by 10 min under dark conditions (trace c), FG/acetone after UV irradiation (7.5 min), thawed at room temperature and finally cooled back under dark conditions at *T* = 90 K (trace d), and FG/deuterated acetone (acetone-*d*_6_) recorded at *T* = 90 K after 7.5 min under UV irradiation followed by 10 min under dark conditions (trace e, bottom spectrum). (b) FG radical/acetone system; the charge density difference due to an extra electron (w.r.t. the charge density of the neutral system).

The presence of polaron states in the FG acetone system was corroborated by further EPR experiments. Starting from the silent EPR signal observed under dark conditions (Figure 4, trace a), upon in situ irradiation for 3 min followed by immediately cutting off the light, spectrum b was recorded within the selected 2 min of acquisition time (using 0.5 mT modulation width). The signal can be simulated with *S* = 1/2 for the Zeeman term (*g*_e_-tensor (*x*,*y*,*z*) = 2.0013, 2.0013, 2.0013) plus the contribution from the hyperfine interactions (gβ_e_BS_*z*_ + Σ(A_0_)S_*z*_I_*z*_) with three H atoms associated with the acetone molecule (1:3:3:1 pattern, *A*-tensor for ^1^H = 2.25, 2.25, 2.25 mT). The EPR spectrum simulation of the recorded pattern is given in the Supporting Information (Figure S12). After irradiation for 7.5 min followed by 10 min under dark conditions, spectrum b converted to trace c, which was largely asymmetric and much richer in hyperfine components. Spectrum c converted to spectrum d upon thawing to room temperature and recooling down to 90 K under dark conditions. No detectable residual or stable spins remained in the FG system after thawing.

In order to understand if these resonance components resulted solely from ^1^H nuclei from the acetone molecules or whether ^19^F nuclei from FG contributed to the recorded signal, we performed the same experiments using deuterated acetone solvent (acetone-*d*6) under identical experimental conditions except for the smaller modulation width of 0.05 mT used to record the resonance signal (Figure 4, trace e). A clear septet was observed, corresponding to spin density residing on six fluorine nuclei (*g*_e_-tensor (*x*,*y*,*z*) = 2.0005, 2.0005, 2.0005; *A*-tensor for ^19^F = 0.34, 0.34, 0.34 mT) overlapped to a second broad resonance component. Therefore, the central (derivative) signal detected in the spectrum (c) around *g* = 2.0005 arose from the e^–^ couple counterpart of the polaron pair system that was located on the FG backbone. The EPR spectrum simulation of the recorded pattern and additional LEPR spectra using an in situ acquisition sequence such as light-off, light-on, and light-off (2.5, 7.5, and 10 min, respectively) is given in the Supporting Information (Figure S13, Figure S14). The side signal was generated by a hole located on the acetone molecule interacting with FG. The fact that the EPR signal in the acetone-*d*_6_ system was well resolved in the ^19^F hyperfine components indicates that the electron–hole pairs were strongly bound together by the Coulomb interaction. The strong Coulomb interaction occurred due to the low screening characteristic of FG^19,23^ and of two-dimensional materials in general.^24^ Thus, the magnetically active photoexcited state can be considered a quasiparticle system, encompassing net 1/2 spin moment. This interpretation was corroborated by additional periodic DFT calculations for the FG radical/acetone system. When an electron was added to the system, the negative charge strongly localized at the defect site (Figure 4b). The atoms in the vicinity of the radical site relaxed their positions; i.e., the polaron was formed. The polaron was as small as two adjacent carbon p_*z*_ orbitals of the F radical defect. The atomic displacements were highly localized, and only the first shell of F atoms around the polaron center exhibited non-negligible distortions from their initial positions. The bond between the radical and three adjacent carbon atoms is shortened by 0.05 Å, while the C–F distance from the radical to the nearest F atom is increased by 0.08 Å. The C–F bond length of these three F atoms is increased by 0.07 Å due to the presence of the polaron. The relaxation energy due to polaron formation was 0.634 eV. These signatures are fingerprints of a Holstein-type polaron. The acetone molecule interacted with the polaron since the charge density accumulated at the interface between the molecule and the radical defect (Figure 4b and Figure S14) and promoted polaron formation, as the relaxation energy was 0.42 eV without acetone. Notably, when an electron was extracted from the system, forming a hole, the resulting positive charge became localized on the acetone molecule (Figure S16). Presumably, the acetone frozen matrix surrounding FG may have served as a hole transport layer,^25^ allowing further excitation of electrons into FG and formation of other quasiparticles such as trions. Kwon et al. demonstrated that when a polaron was localized with the exciton at the chemically engineered charge defect centers, brightly fluorescent trions were produced.^26^

## Conclusions

In conclusion, our findings underscore the crucial role of the solvent environment in initiating the significant generation of spin-active states in the 2D graphene derivative upon UV irradiation. For FG freshly dispersed in benzene and acetone, the EPR spectra changed dramatically upon irradiation; a very strong asymmetric resonance signal was detected in acetone solution and a weak one but still discernible in benzene. The presence of the solvent was crucial, as there was no difference between the EPR spectra in the dark and under UV light for the neat FG powder and for the blank samples containing only the acetone or the benzene solvent. As for the acetone solvent, the dynamics of the photoexcitation processes showed significant reorganization during the light-on to light-off time window due to changes in the spatial configuration of the acetone molecules interacting with the fluorographene sheets. The spin systems generated by UV light can be interpreted as bound polaron pairs (h^+^, solvent/e^–^, FG sheets), which has been supported by theoretical calculations showing polaron formation in the radical defects of the FG promoted by acetone. Our work demonstrates that besides the well-established targeted engineering of defects in materials aimed at producing polarons, trions, qubits, etc., additional effort should be devoted to understanding their mutual interactions with the environment, which could be enhanced by finding the best solvent (or solvent mixture) for the desired process. The observation of the solvent tunable polaron formation in the lightweight and the easily accessible nanomaterial opens up a wide range of applications including, e.g., not only molecular sensing or magneto-optical devices but also hole-transport layers in perovskite solar cells and N–V defects in diamond, which holds potential for qubit realization in quantum computing.

## Experimental Section

The periodic DFT calculations were performed using the projector-augmented wave method implemented in the Vienna ab initio simulation package (VASP) suite.^27^ The potentials used in the present work were the latest GW potentials distributed with VASP (vasp.6.3). The primitive cell of FG was created by adopting the chair conformation^28^ and the in-plane lattice parameter *a* of 1.29 Å.^29^ A supercell approach was used to model the surface of FG including the radical defect (fluorine vacancy); we adopted the 6 × 6 supercell (72 carbon and 72 fluorine atoms) and modeled the radical defect by removing single fluorine atom followed by structural relaxation. The energy cutoff for the plane-wave expansion was set to 400 eV, and the 3 × 3 × 1 *k*-point grid was used to sample the Brillouin zone of the supercell. The geometry of the radical defect and of the physisorbed acetone was attained by using the optimized van der Waals DFT functional (optB86b-vdW),^30^ which included nonlocal correlation effects and provided very accurate results for various graphene–molecule complexes.^31^ The periodically repeated sheets were separated by at least 10 Å of vacuum. The differential charge densities were plotted using the VESTA suite.^32^ The GW calculation was performed on top of the Kohn–Sham orbitals. The Green’s function was iterated self-consistently while keeping the screened potential W at its initial shape (this method is usually denoted as GW_0_). In GW calculation, we kept the 3 × 3 × 1 *k*-point grid (corresponding to the 9 × 9 × 1 *k*-point grid in the unit cell) and the energy cutoff of 400 eV. We added a large number of empty bands so GW calculations were performed using at least 1500 unoccupied bands, which is essential for their convergency.^19^ The Bethe–Salpeter equation was solved on top of the GW_0_ calculation using the same set of computational parameters. It should be noted that a GW/BSE calculation may need a very dense *k*-point mesh and high energy cutoff to fully converge,^33^ which is extremely computationally demanding for the large supercell used in this work. Based on previous study for FG^19^ and our tests for the acetone molecule, we estimate that our calculation is converged to within a few meV. In addition, the TD-DFT was used for the calculations involving the large 6 × 6 supercell. The test calculation showed that the TF-DFT reproduced well both the FG optical and electronic band gaps when the hybrid functional PBE0^34^ was used as the kernel (Figure S17) and thus the TD-DFT could be used for larger-scale calculations.

The structures of finite size model systems were optimized employing the ωB97X-D functional^35^ in combination with the 6-31++G(d,p) atomic basis set.^36^ In all cases, vibrational analysis was performed and the absence of imaginary frequencies was checked to confirm the character of the stationary points on the potential energy surface. Bulk solvent effects (apart from an explicit solvent molecule) were accounted for by using implicit solvation model based on density (SMD).^37^ Electronic transitions were explored using the TD-DFT employing the CAM-B3LYP functional^38^ with the same basis set. All calculations were performed using the Gaussian16 program.^39^

Detailed chemical and spectroscopic characterization of the commercial FG material employed in this study is given in the Supporting Information (graphite fluorinated polymer, Sigma-Aldrich, CAS number: 51311-17-2, linear formula: (CF*x*)_*n*_, *x* ∼ 1.1). Continuous wave electron paramagnetic resonance spectra and light induced spectra were recorded on a JEOL JES-X-320 spectrometer (JEOL, Tokyo, Japan) operating at the X-band frequency (∼9.0–9.1 GHz) equipped with a variable-temperature controller (He, N_2_) ES-CT470 apparatus. Highly pure quartz tubes were employed (Suprasil, Wilmad, ≤0.5 OD) as sample holders, and accuracy on the experimentally determined *g*-values were obtained by using Mn(II)/MgO standard (JEOL standard). The cavity quality factor (*Q*) was kept above 6500 in all measurements. Filling factors were kept the same (200 μL) in all experiments. The EPR and LEPR spectra were acquired under non power saturating conditions, upon careful control of the applied microwave power during signal acquisition. In situ light excitation EPR experiments (CW-LEPR) were performed by deploying a HeCd laser source operating at 325 nm (max output power of 200 mW) from Kimmon Koha Co. Ltd. (Tokyo, Japan). The UV-light was shined directly onto the sample, kept frozen inside the cavity EPR resonator, and by using a dedicated optical fiber fitted through the resonator optical window. The in situ monitoring of the UV light-off to light-on process was operated by a light on–off shutter mechanism.

## Supporting Information Available

The experimental raw data (ASCII format) associated to the X-band EPR spectra given in Figures 1, 2, and 4a, main text; chemical and spectroscopic characterization of commercial as well as of newly synthesized FG nanosheets (Fourier transform infrared, XPS, XRD characterization, and SEM images of the pristine and small FG sheets); results of DFT modeling (xyz coordinates for DFT calculations using local models; and structures and output files for periodic DFT models) (ZIP)
Chemical and spectroscopic characterization of commercial as well as of newly synthesized FG nanosheets; raw data from EPR experiments; Fourier transform infrared, XPS, XRD characterization, and SEM images of the pristine and small FG sheets; results of DFT modeling; *xyz* coordinates for DFT calculations using local models; and structures and output files for periodic DFT models (PDF)
