# Supplementary figures and images for: Solvent Controlled Generation of Spin Active Polarons in Two-Dimensional Material under UV Light Irradiation
Source: J Am Chem Soc. 2024 May 2;146(22):15010–8. doi: 10.1021/jacs.3c13296 (PMC11157526; doi:10.1021/jacs.3c13296)

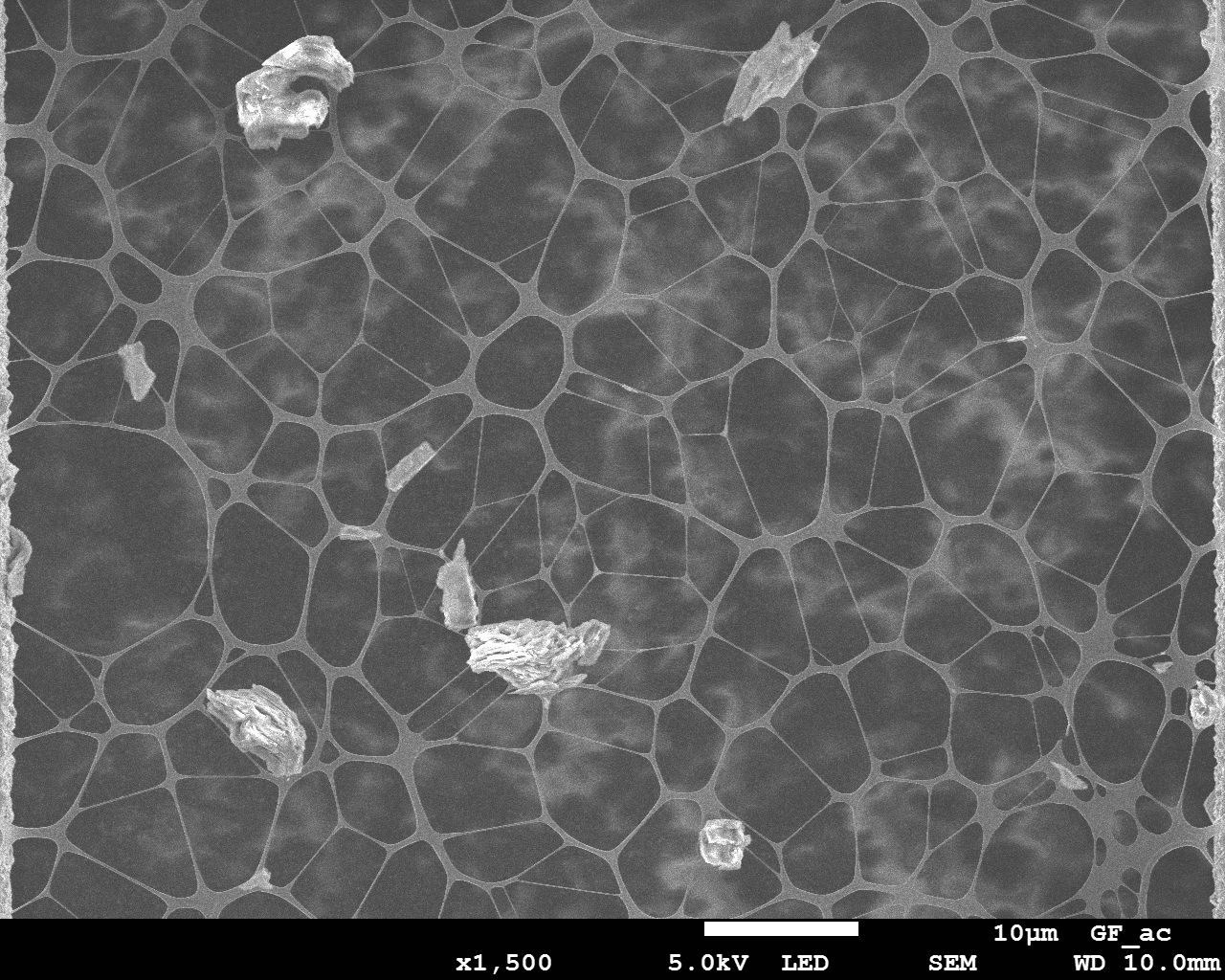

Supplement: Supplementary file 1 — ja3c13296_si_001.zip [file ja3c13296_si_001.zip › Data_archive/SEM/SEM FG size/GF_ac_im001.jpg]

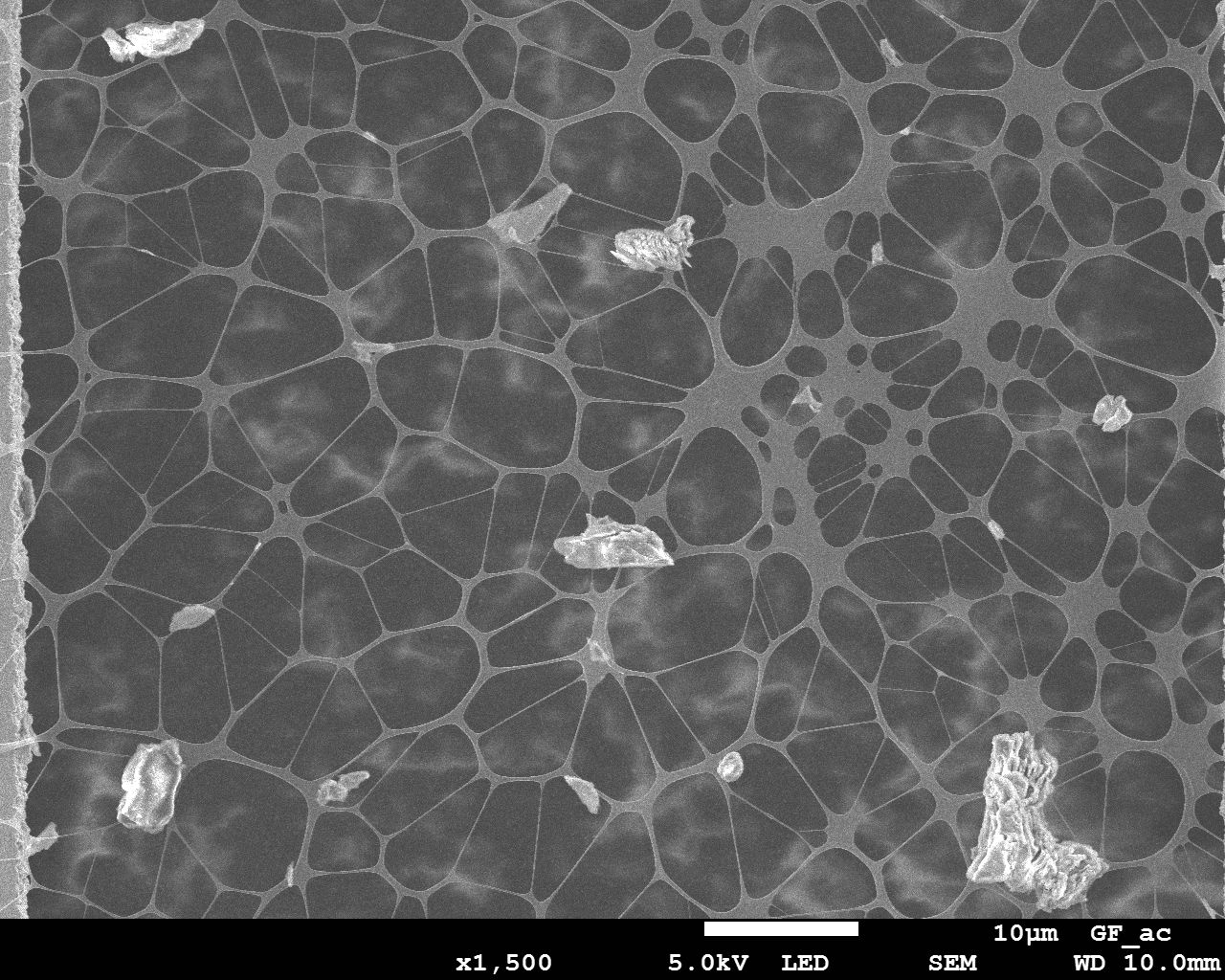

Supplement: Supplementary file 1 — ja3c13296_si_001.zip [file ja3c13296_si_001.zip › Data_archive/SEM/SEM FG size/GF_ac_im002.jpg]

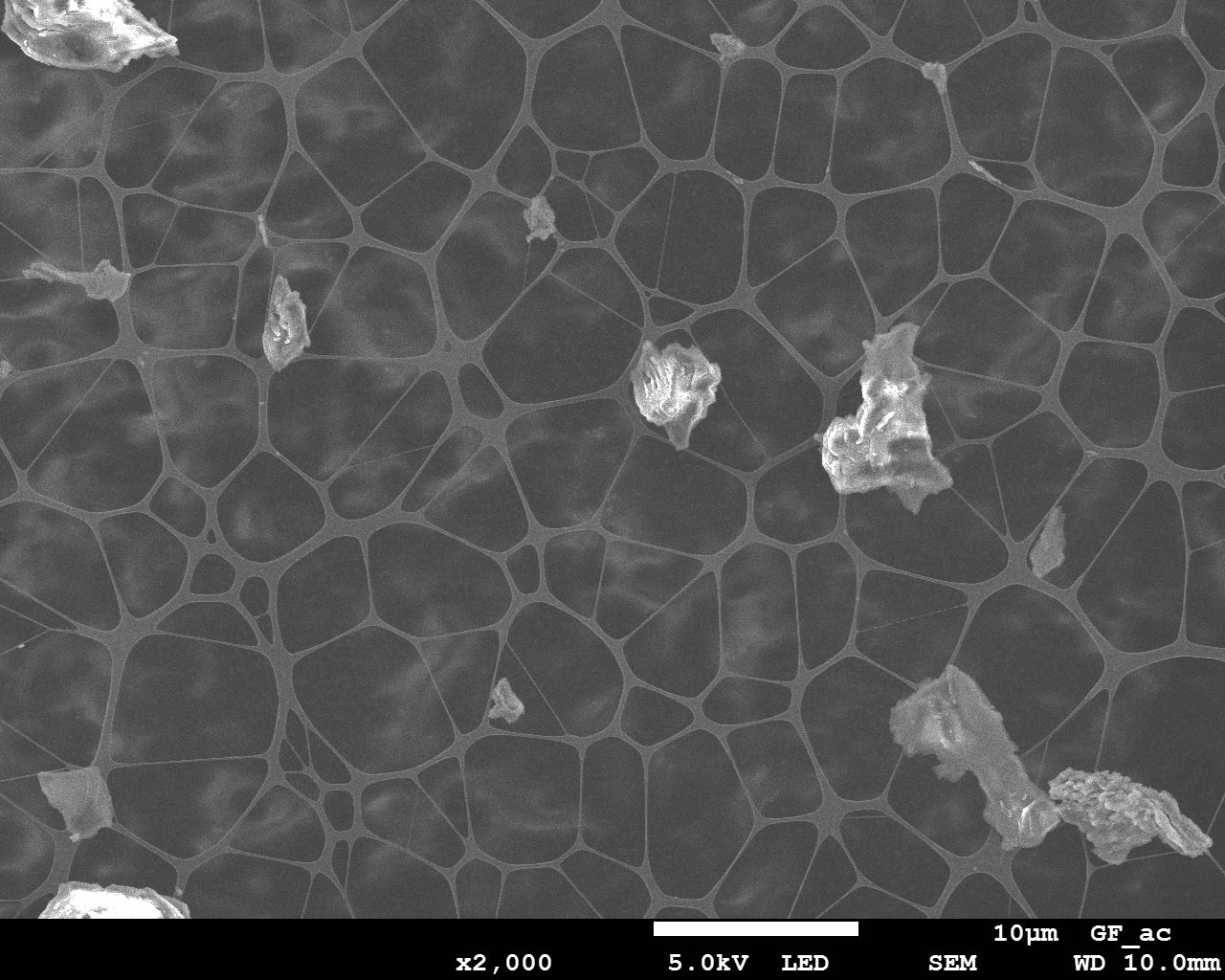

Supplement: Supplementary file 1 — ja3c13296_si_001.zip [file ja3c13296_si_001.zip › Data_archive/SEM/SEM FG size/GF_ac_im003.jpg]

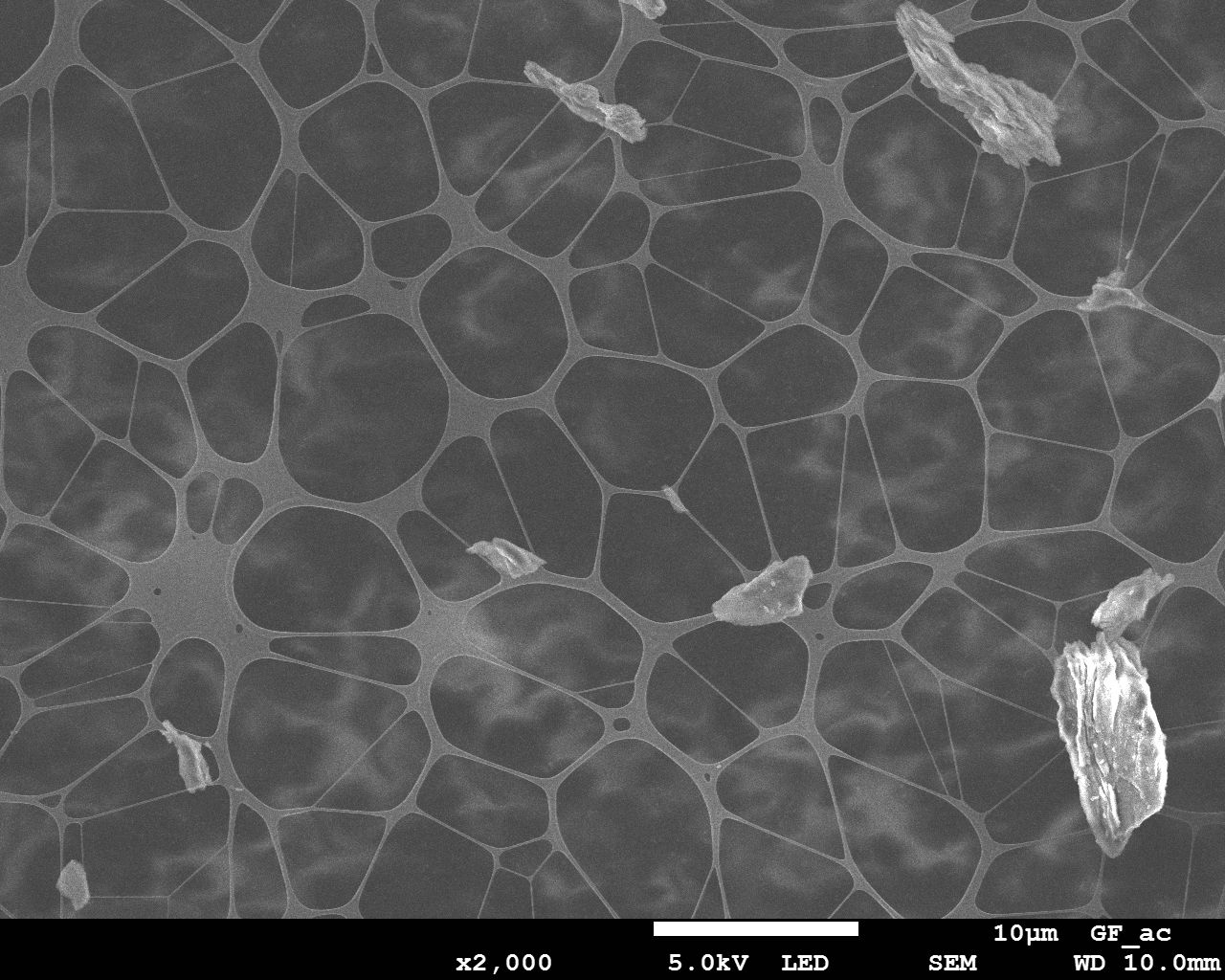

Supplement: Supplementary file 1 — ja3c13296_si_001.zip [file ja3c13296_si_001.zip › Data_archive/SEM/SEM FG size/GF_ac_im004.jpg]

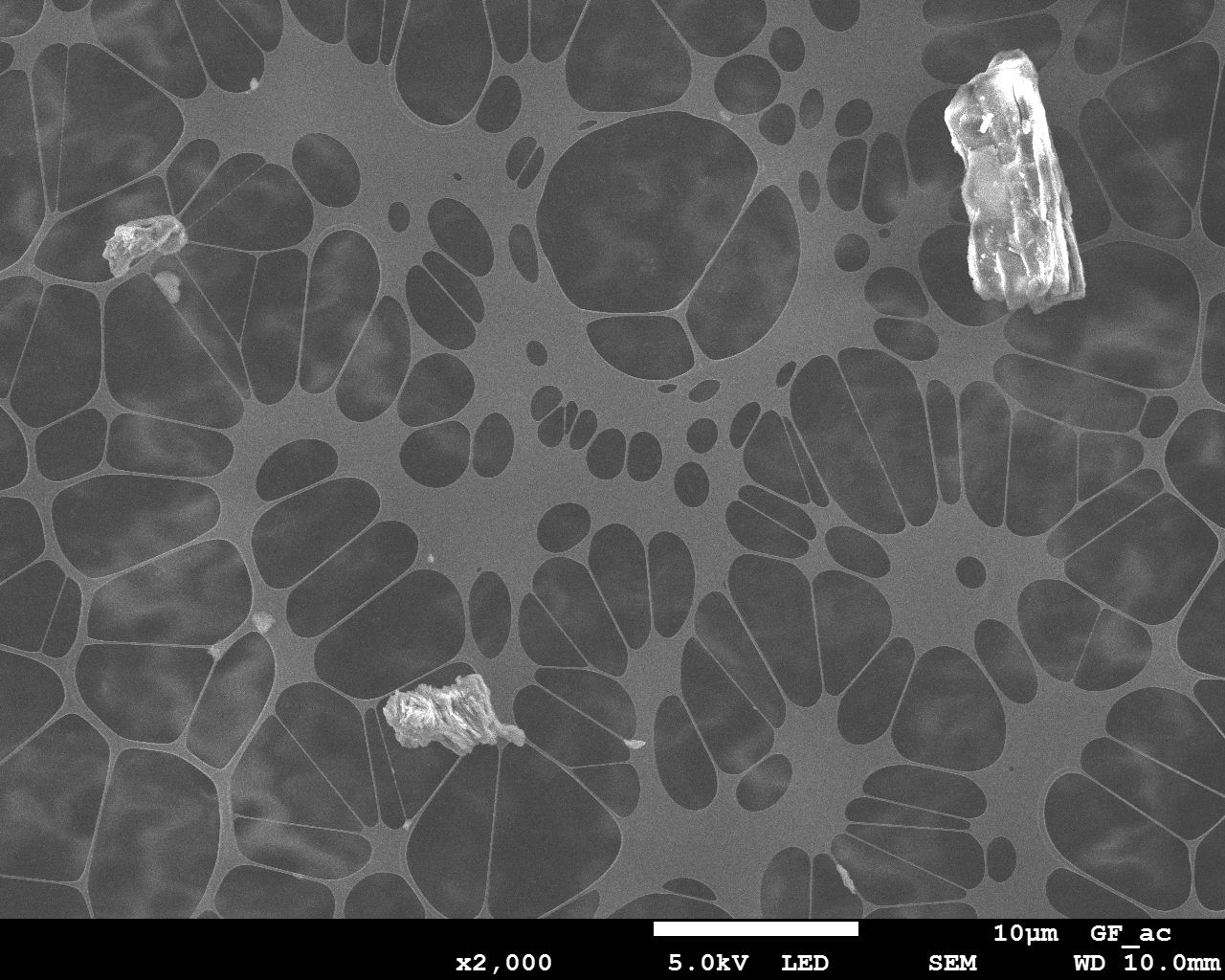

Supplement: Supplementary file 1 — ja3c13296_si_001.zip [file ja3c13296_si_001.zip › Data_archive/SEM/SEM FG size/GF_ac_im005.jpg]

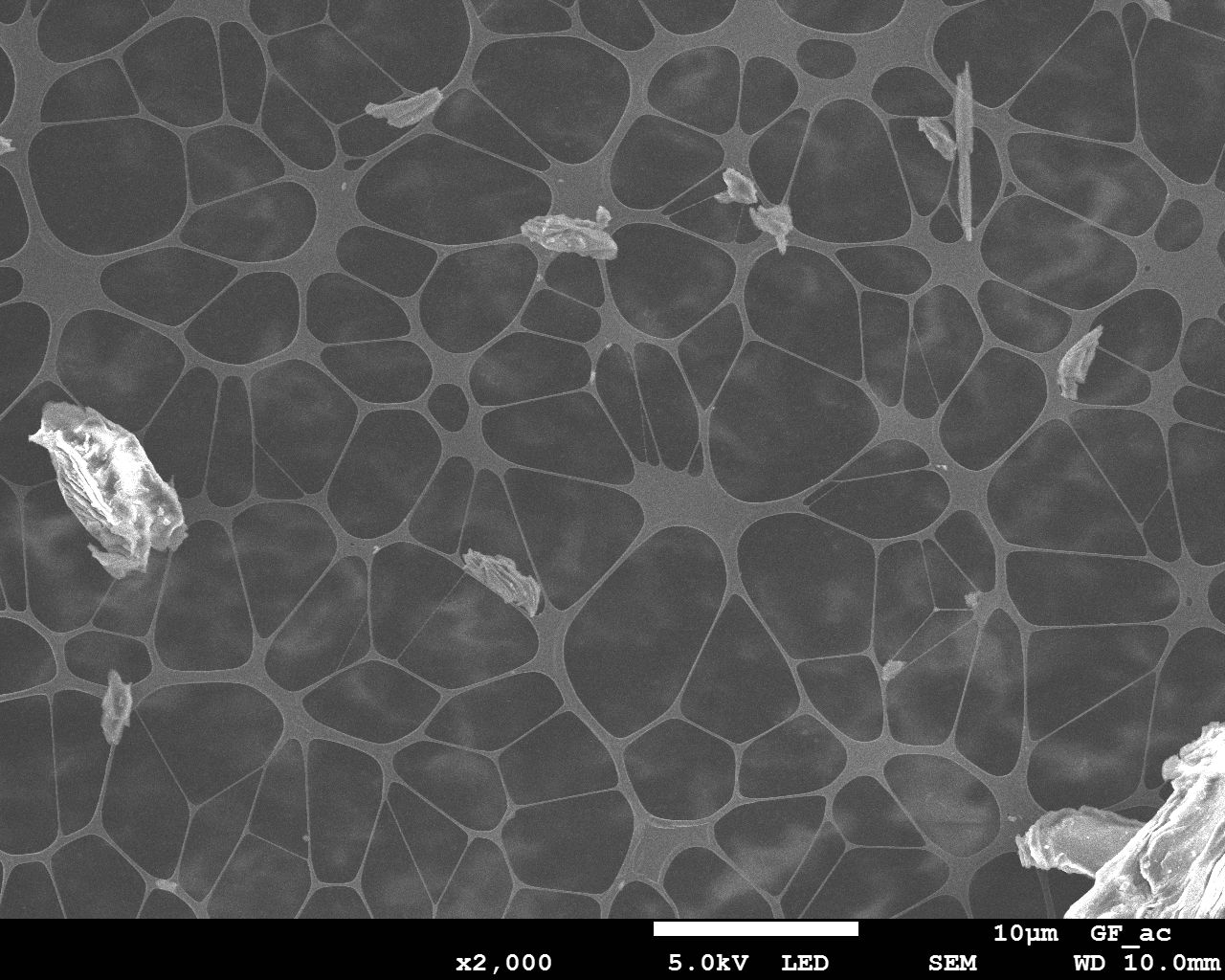

Supplement: Supplementary file 1 — ja3c13296_si_001.zip [file ja3c13296_si_001.zip › Data_archive/SEM/SEM FG size/GF_ac_im006.jpg]

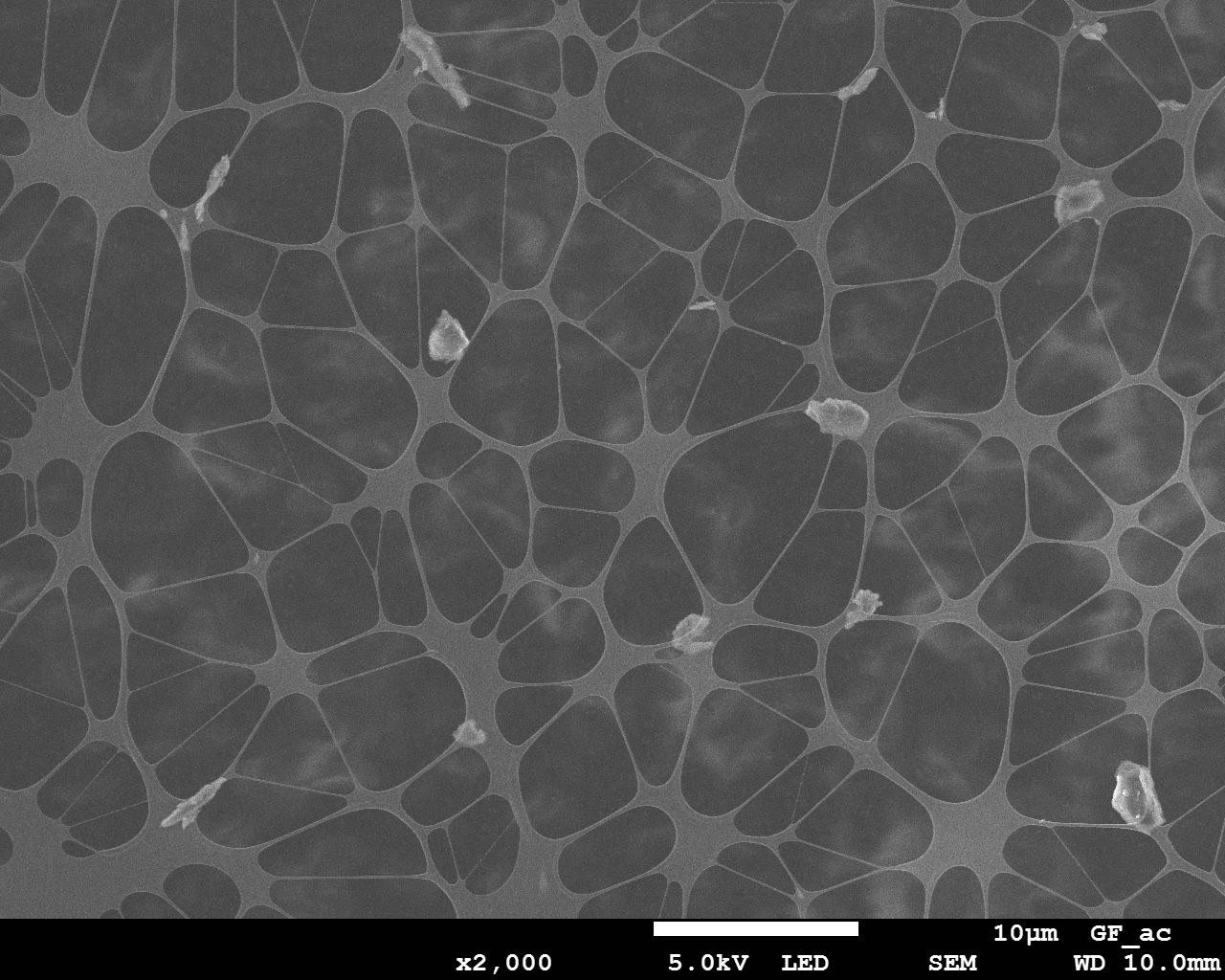

Supplement: Supplementary file 1 — ja3c13296_si_001.zip [file ja3c13296_si_001.zip › Data_archive/SEM/SEM FG size/GF_ac_im007.jpg]

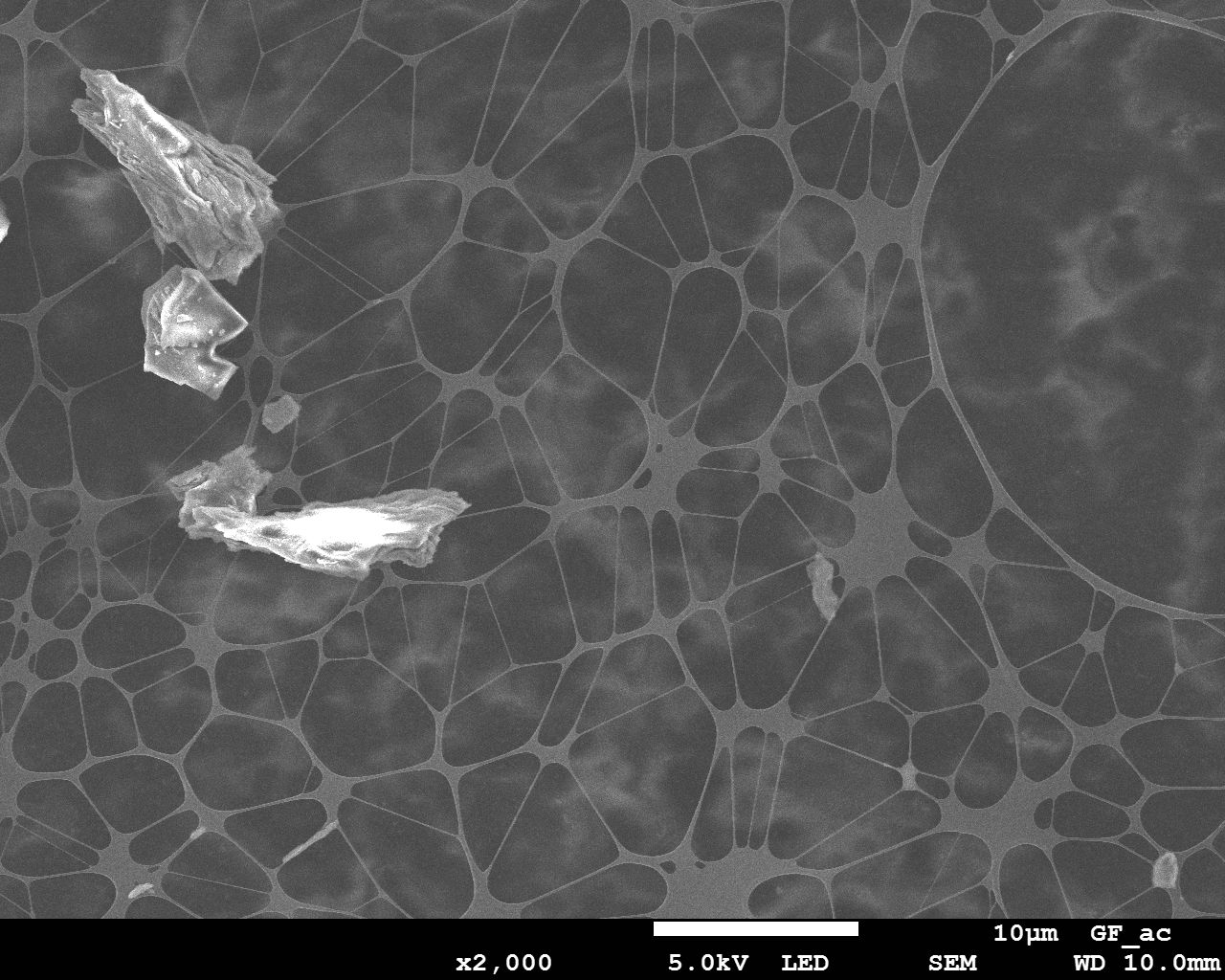

Supplement: Supplementary file 1 — ja3c13296_si_001.zip [file ja3c13296_si_001.zip › Data_archive/SEM/SEM FG size/GF_ac_im009.jpg]

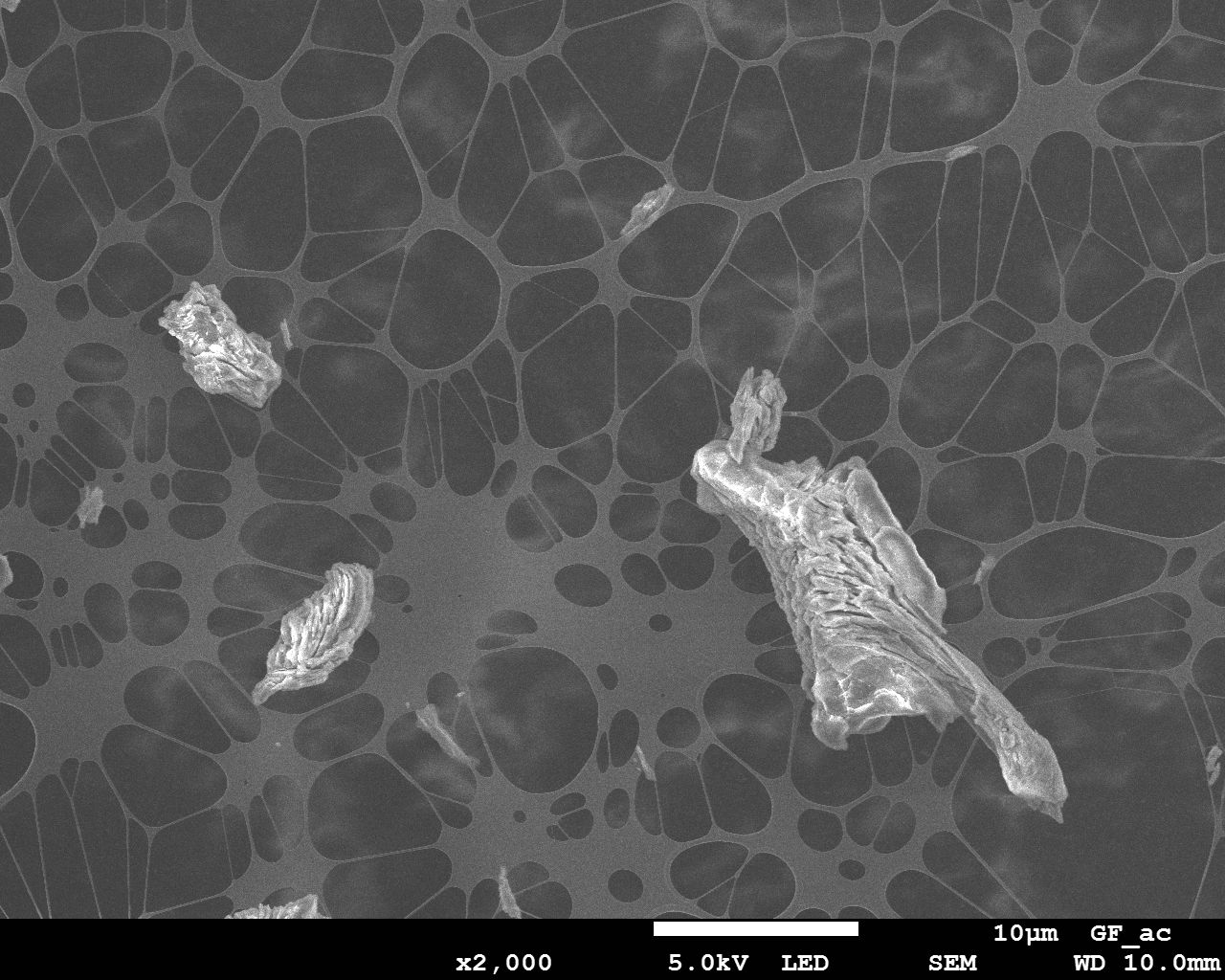

Supplement: Supplementary file 1 — ja3c13296_si_001.zip [file ja3c13296_si_001.zip › Data_archive/SEM/SEM FG size/GF_ac_im010.jpg]

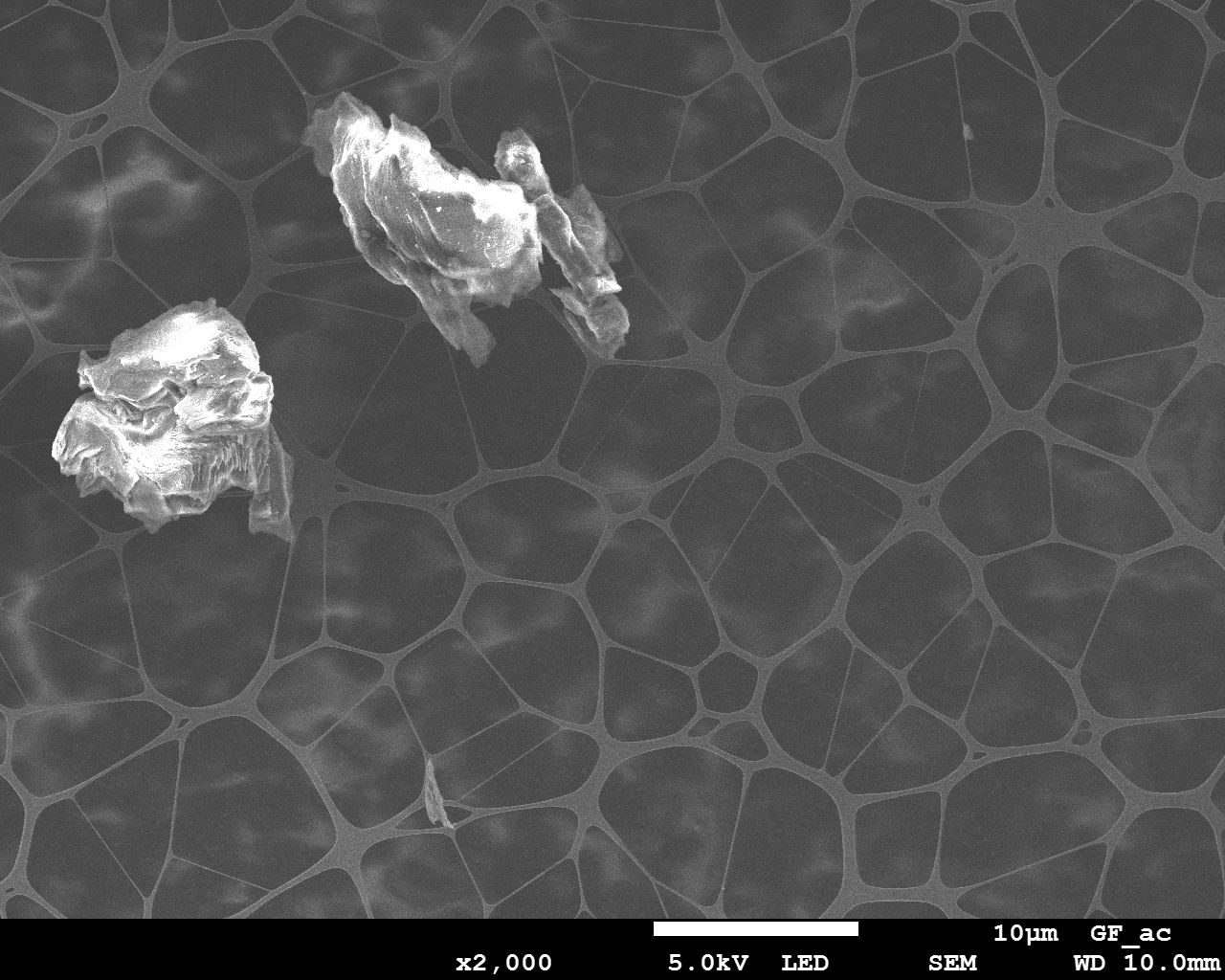

Supplement: Supplementary file 1 — ja3c13296_si_001.zip [file ja3c13296_si_001.zip › Data_archive/SEM/SEM FG size/GF_ac_im011.jpg]

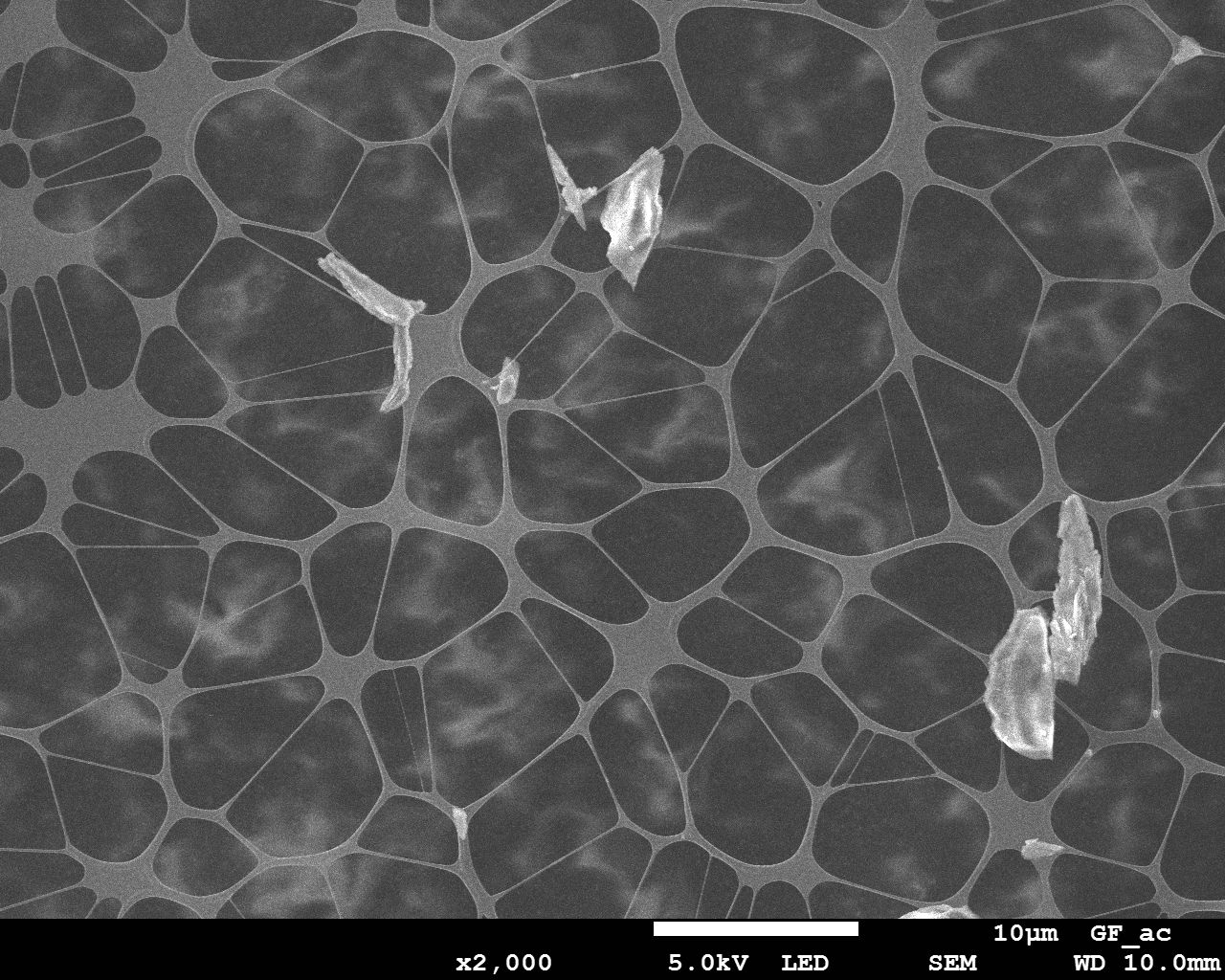

Supplement: Supplementary file 1 — ja3c13296_si_001.zip [file ja3c13296_si_001.zip › Data_archive/SEM/SEM FG size/GF_ac_im012.jpg]

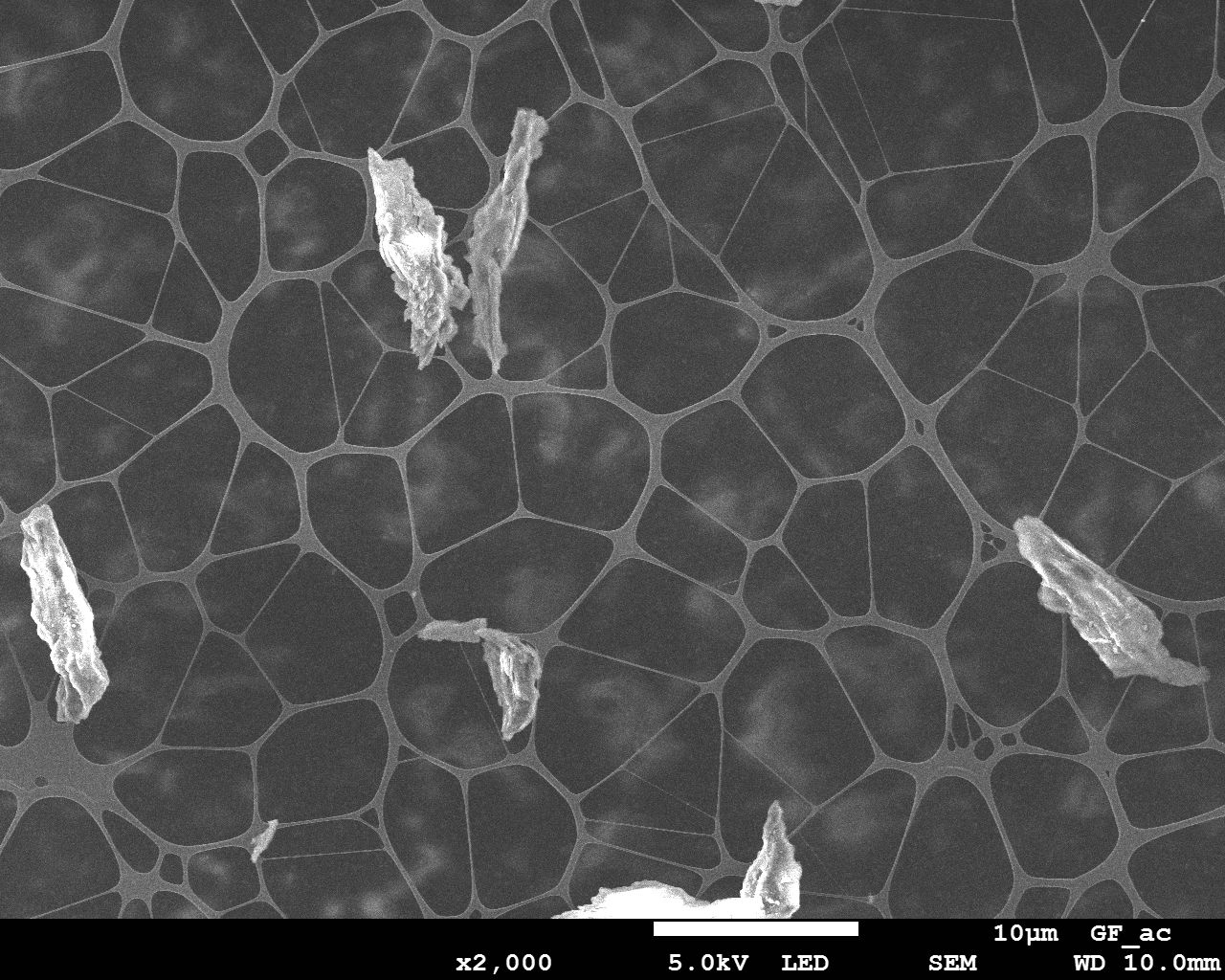

Supplement: Supplementary file 1 — ja3c13296_si_001.zip [file ja3c13296_si_001.zip › Data_archive/SEM/SEM FG size/GF_ac_im013.jpg]

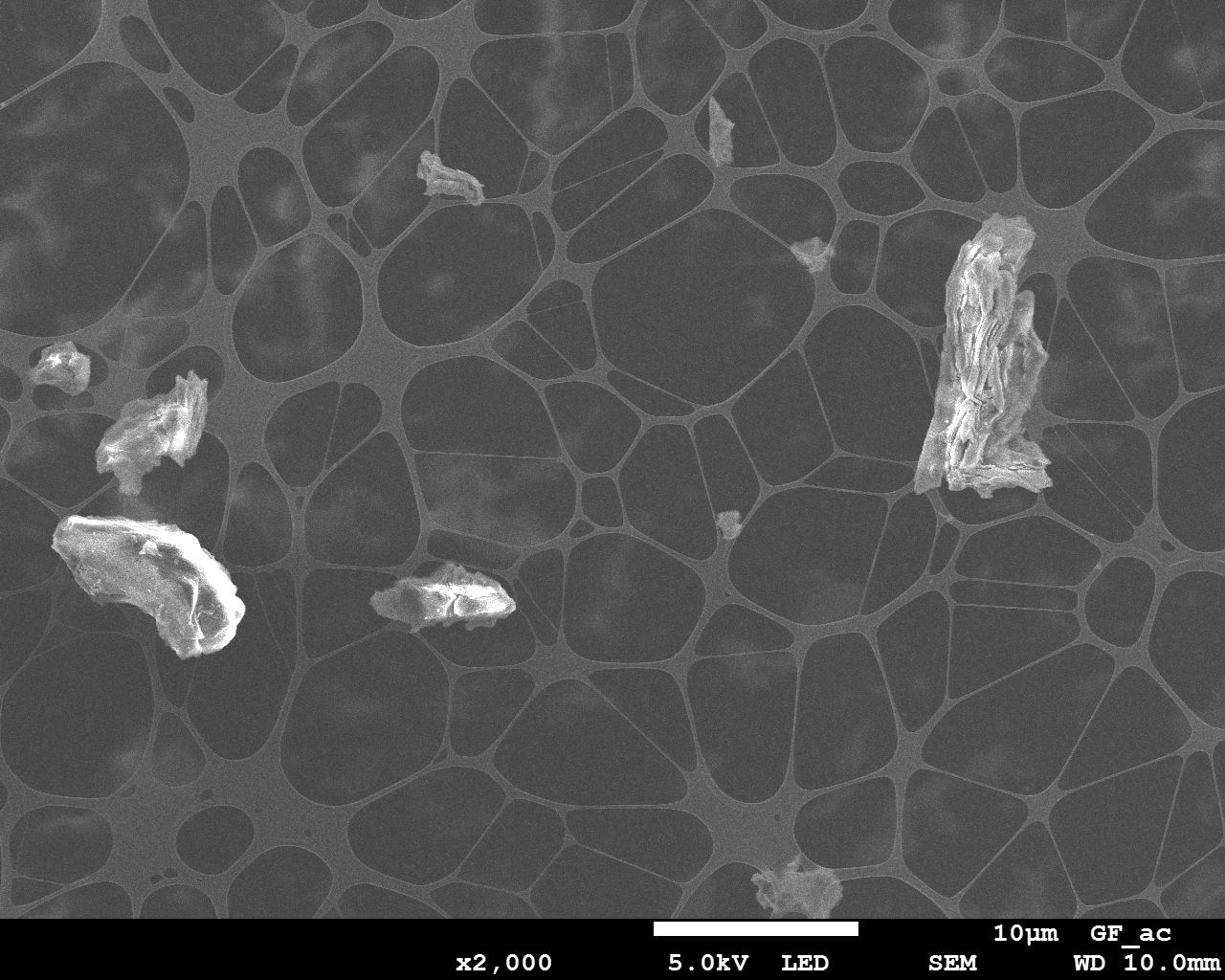

Supplement: Supplementary file 1 — ja3c13296_si_001.zip [file ja3c13296_si_001.zip › Data_archive/SEM/SEM FG size/GF_ac_im014.jpg]

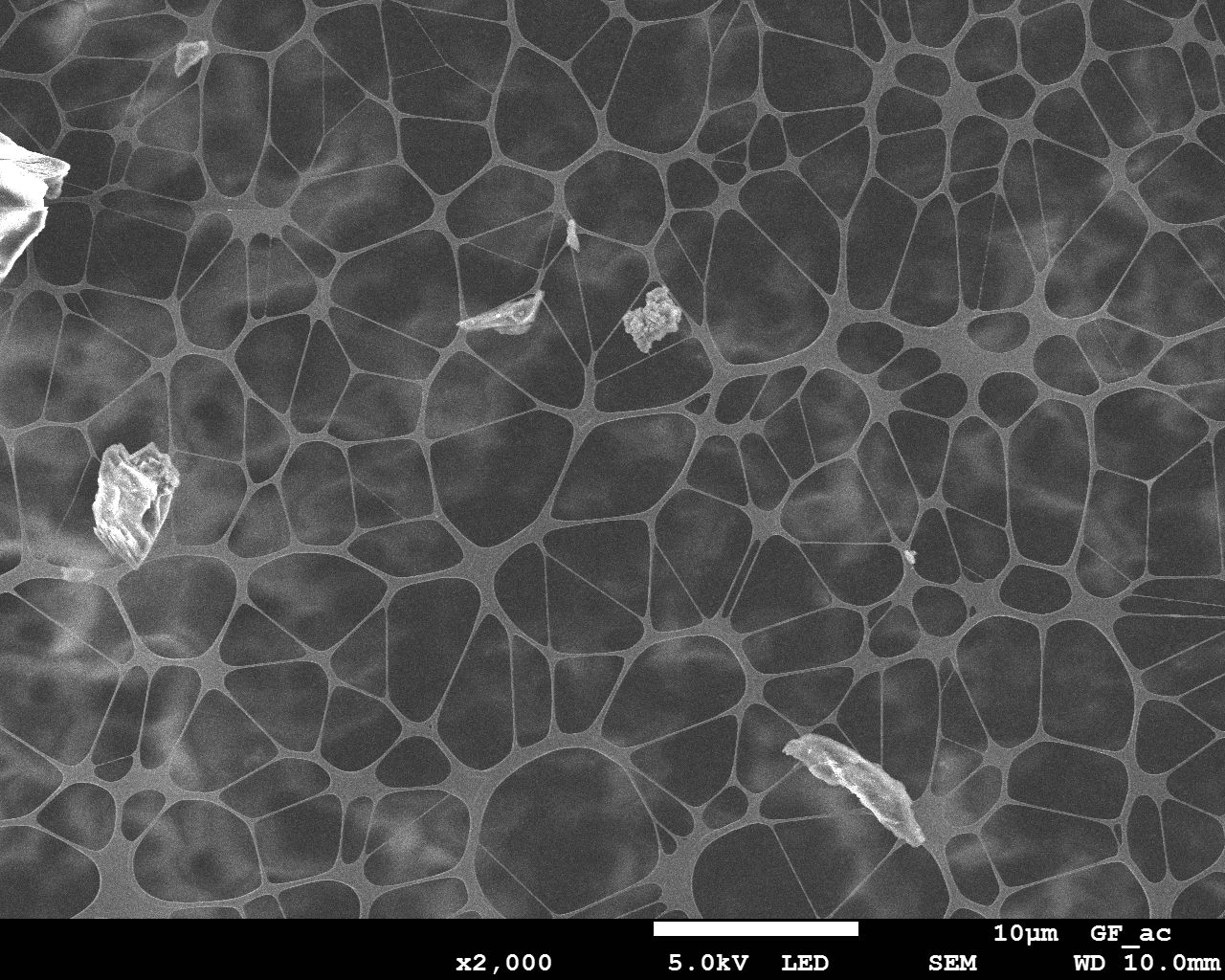

Supplement: Supplementary file 1 — ja3c13296_si_001.zip [file ja3c13296_si_001.zip › Data_archive/SEM/SEM FG size/GF_ac_im015.jpg]

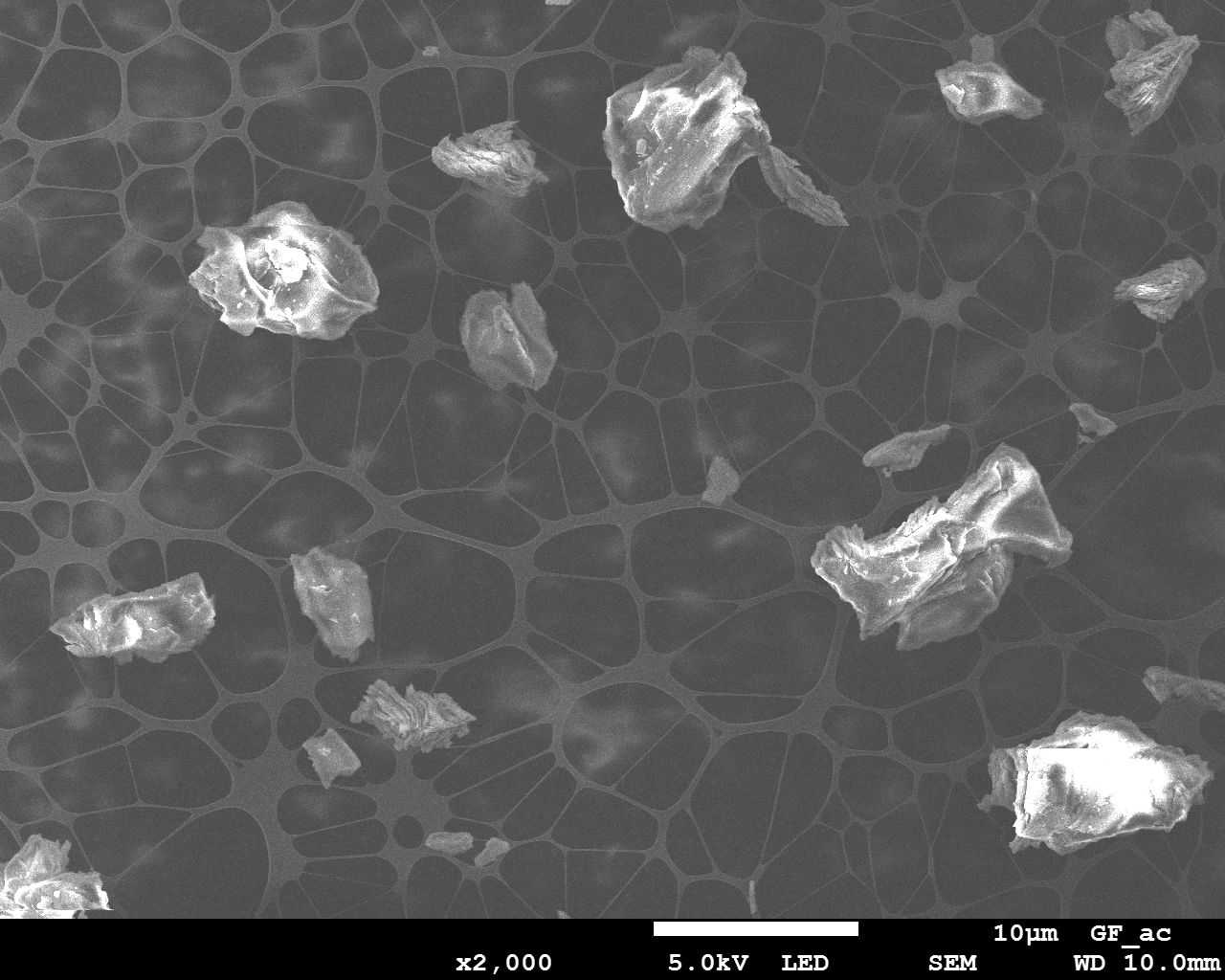

Supplement: Supplementary file 1 — ja3c13296_si_001.zip [file ja3c13296_si_001.zip › Data_archive/SEM/SEM FG size/GF_ac_im016.jpg]

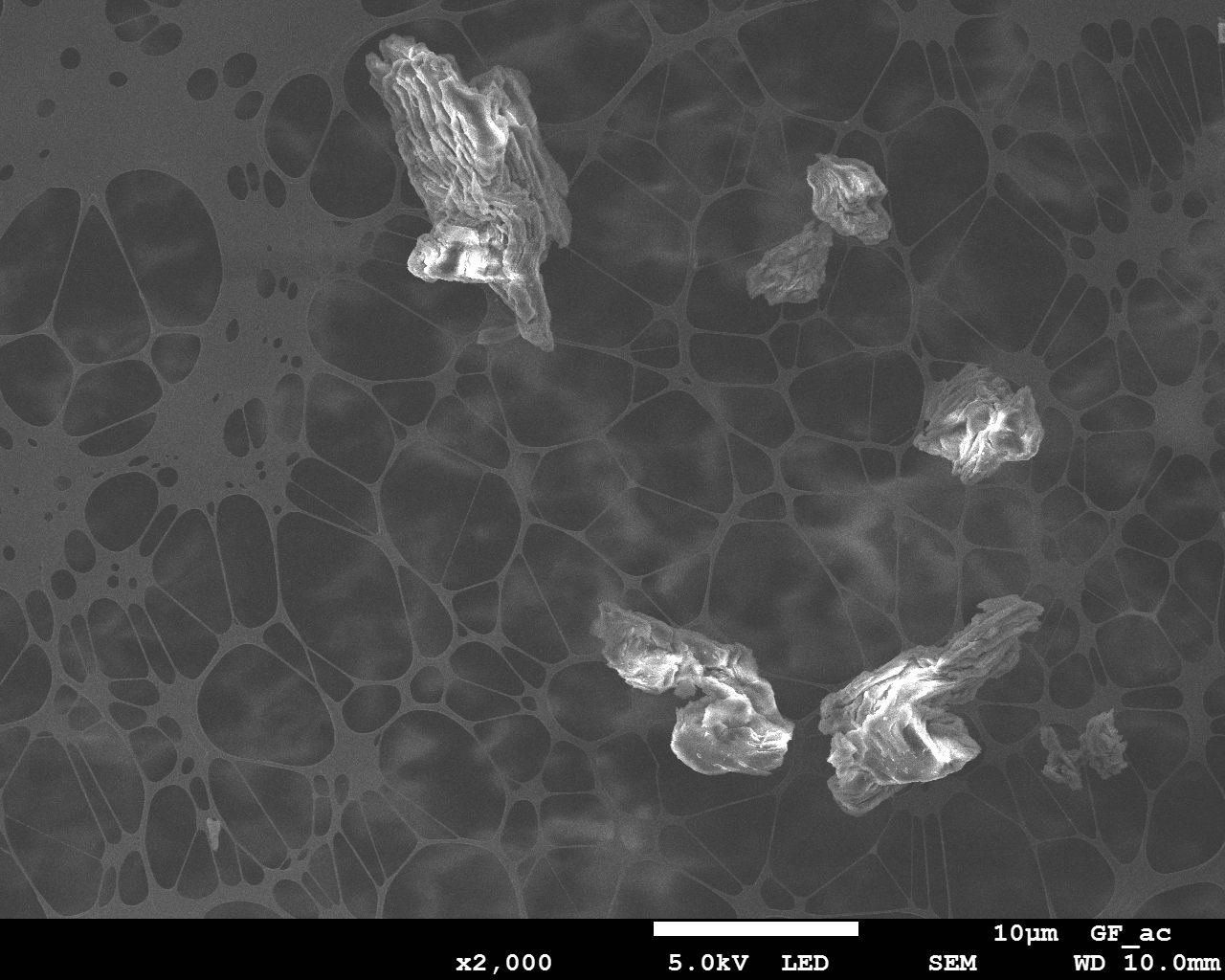

Supplement: Supplementary file 1 — ja3c13296_si_001.zip [file ja3c13296_si_001.zip › Data_archive/SEM/SEM FG size/GF_ac_im018.jpg]

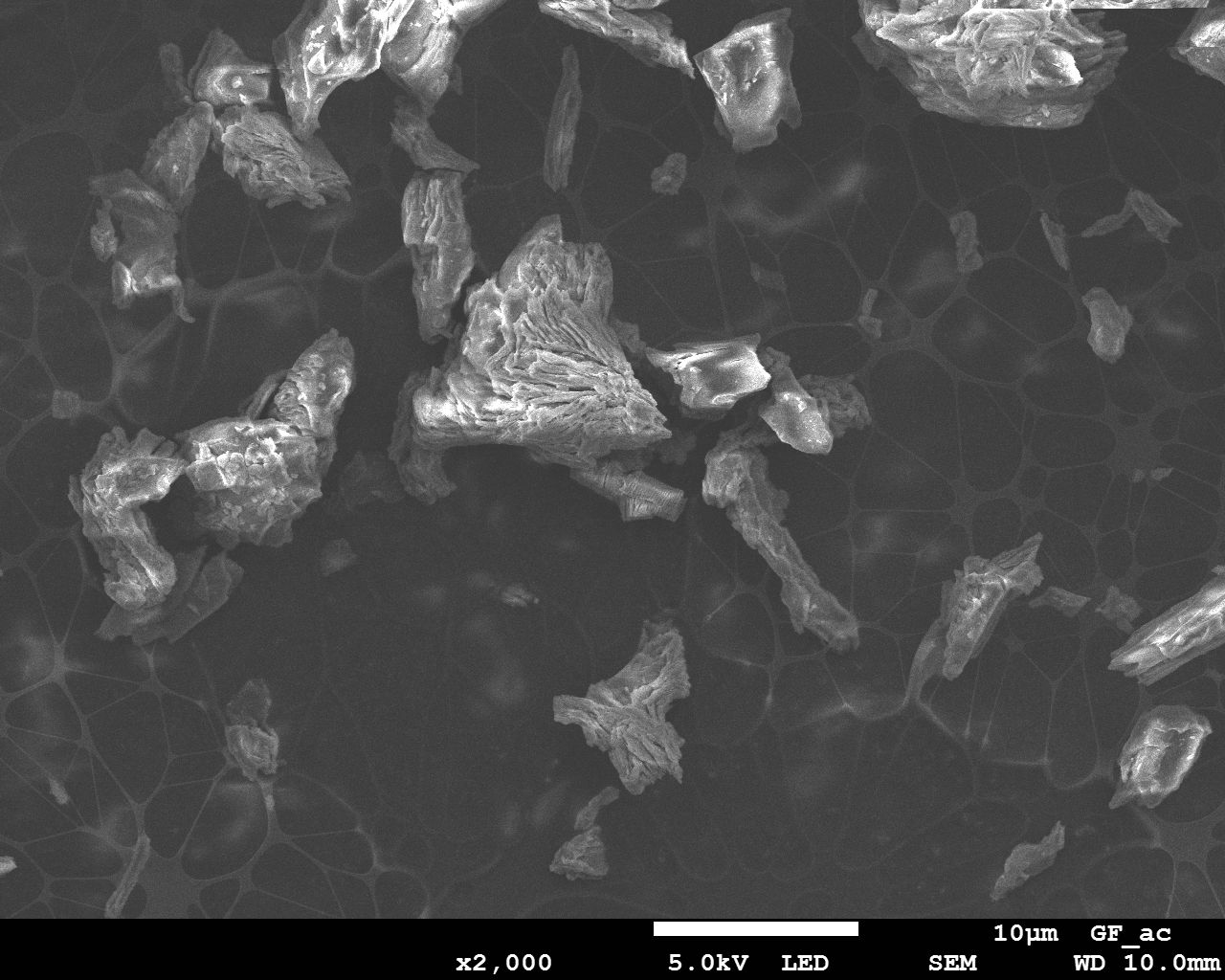

Supplement: Supplementary file 1 — ja3c13296_si_001.zip [file ja3c13296_si_001.zip › Data_archive/SEM/SEM FG size/GF_ac_im019.jpg]

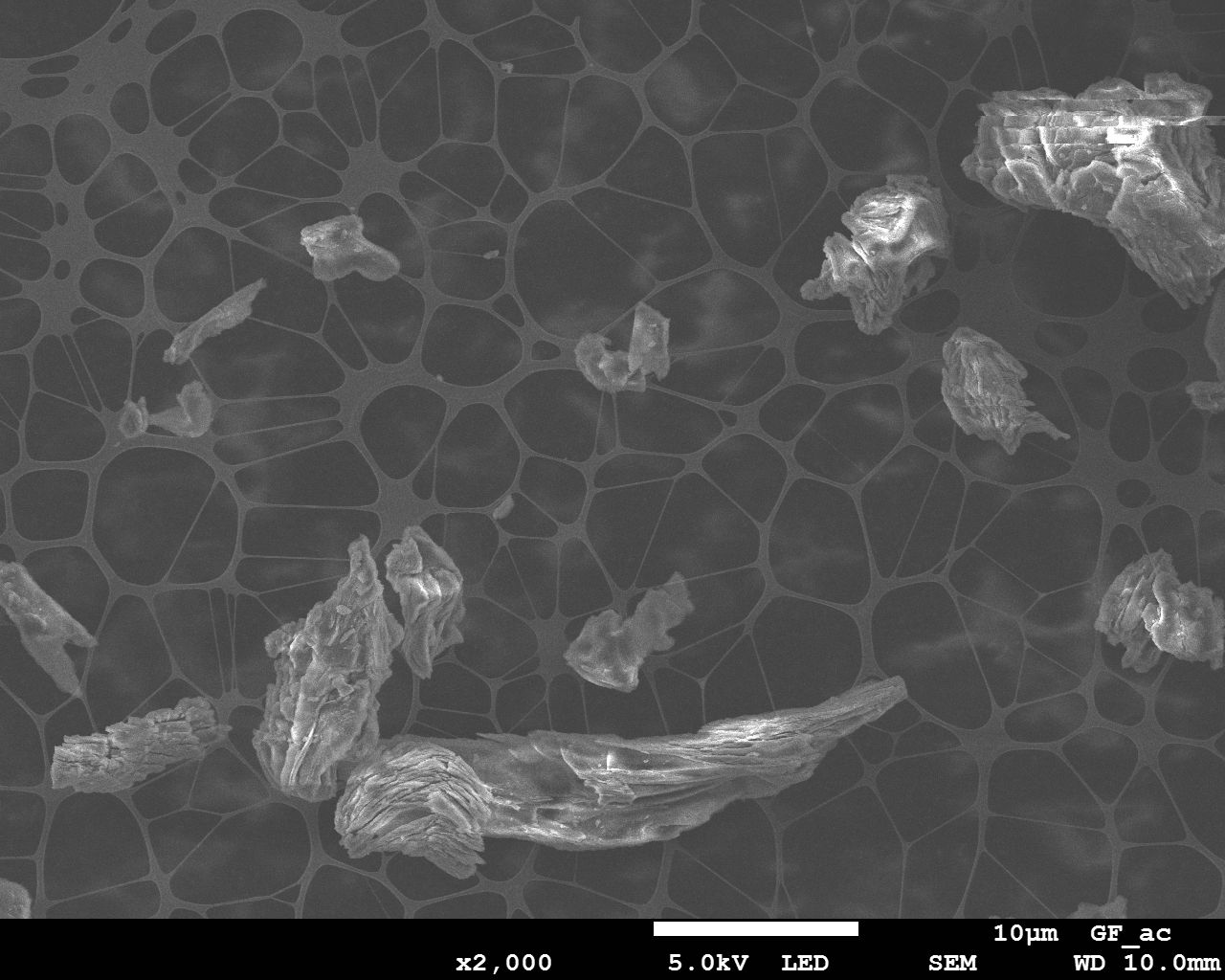

Supplement: Supplementary file 1 — ja3c13296_si_001.zip [file ja3c13296_si_001.zip › Data_archive/SEM/SEM FG size/GF_ac_im020.jpg]

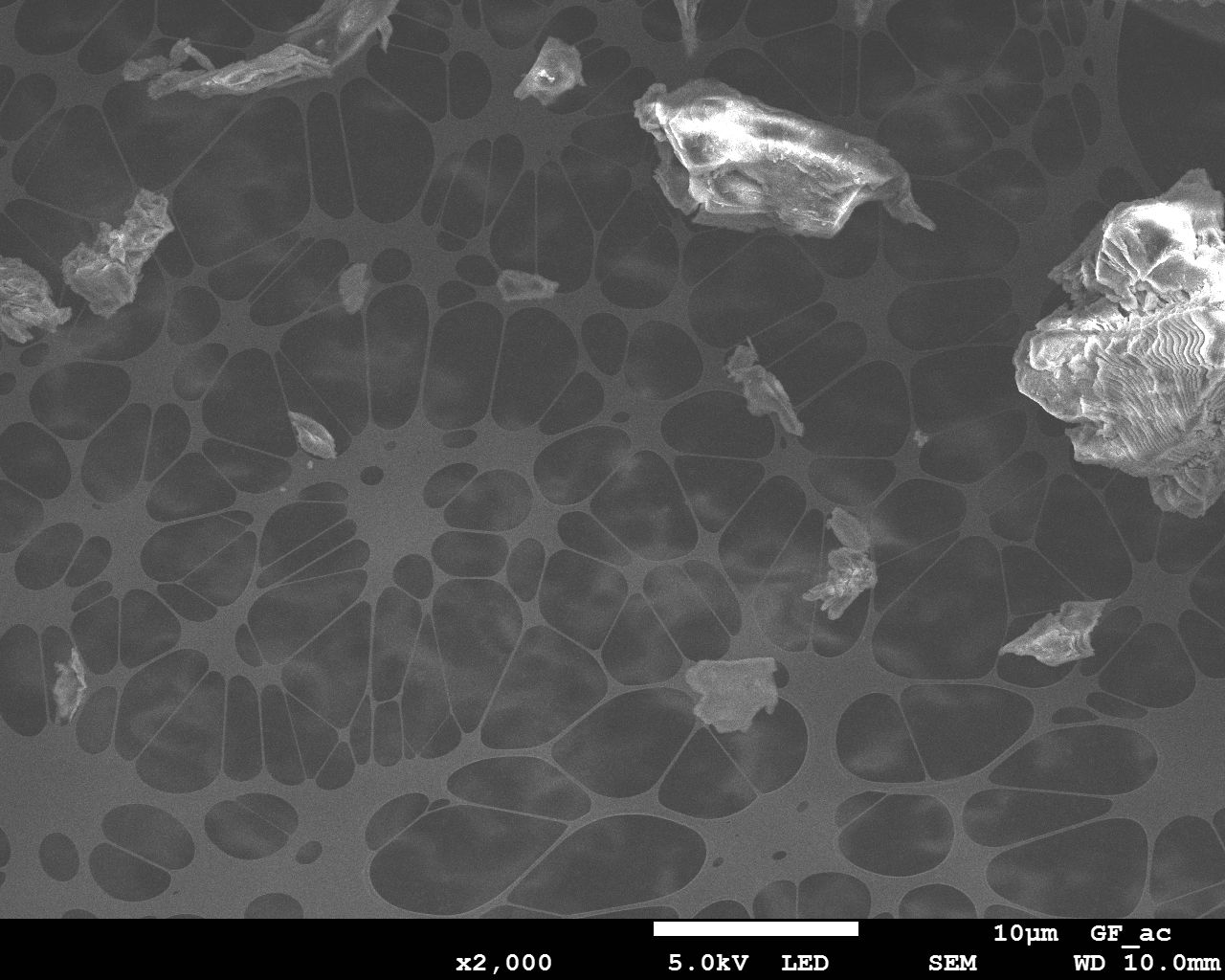

Supplement: Supplementary file 1 — ja3c13296_si_001.zip [file ja3c13296_si_001.zip › Data_archive/SEM/SEM FG size/GF_ac_im021.jpg]

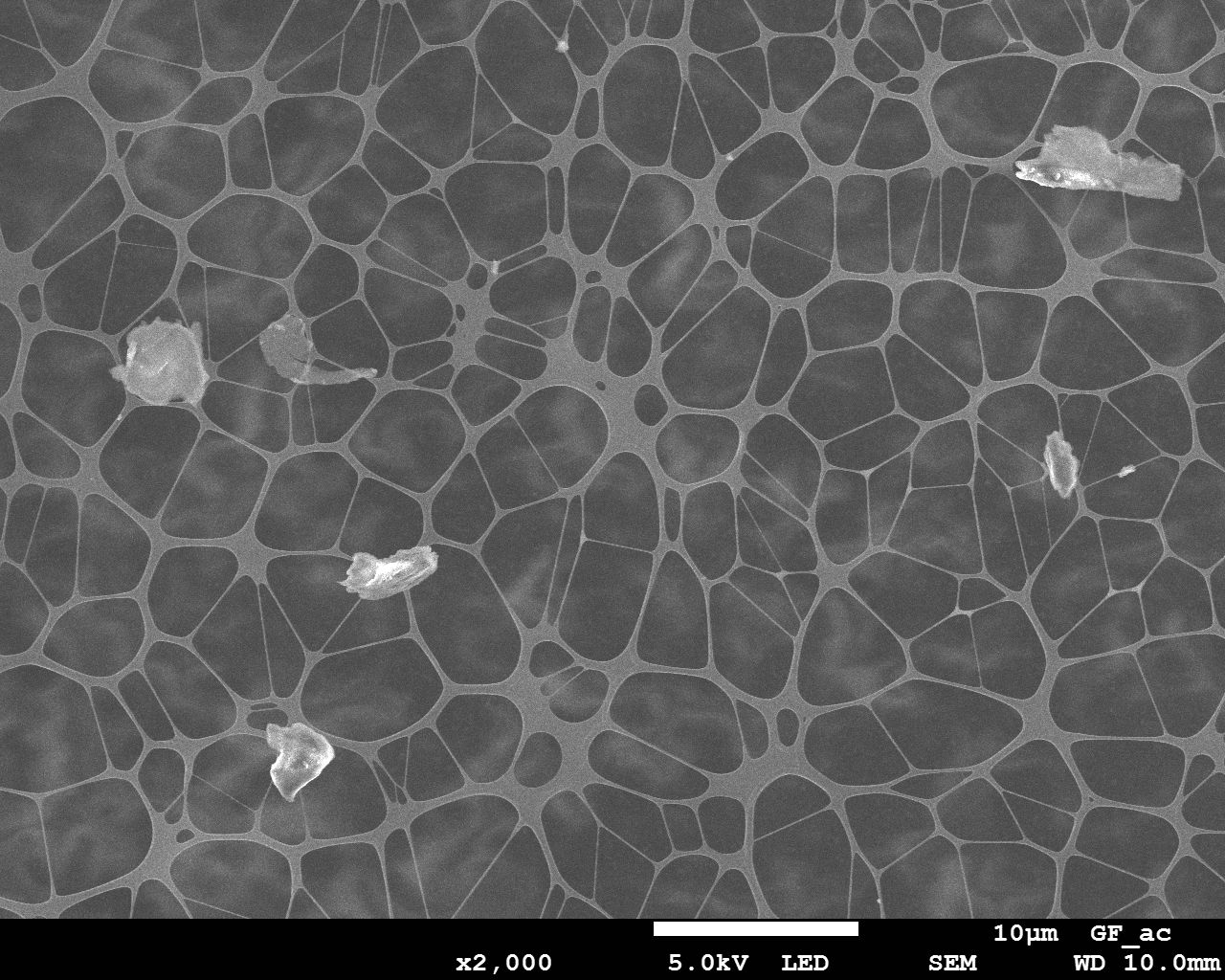

Supplement: Supplementary file 1 — ja3c13296_si_001.zip [file ja3c13296_si_001.zip › Data_archive/SEM/SEM FG size/GF_ac_im022.jpg]

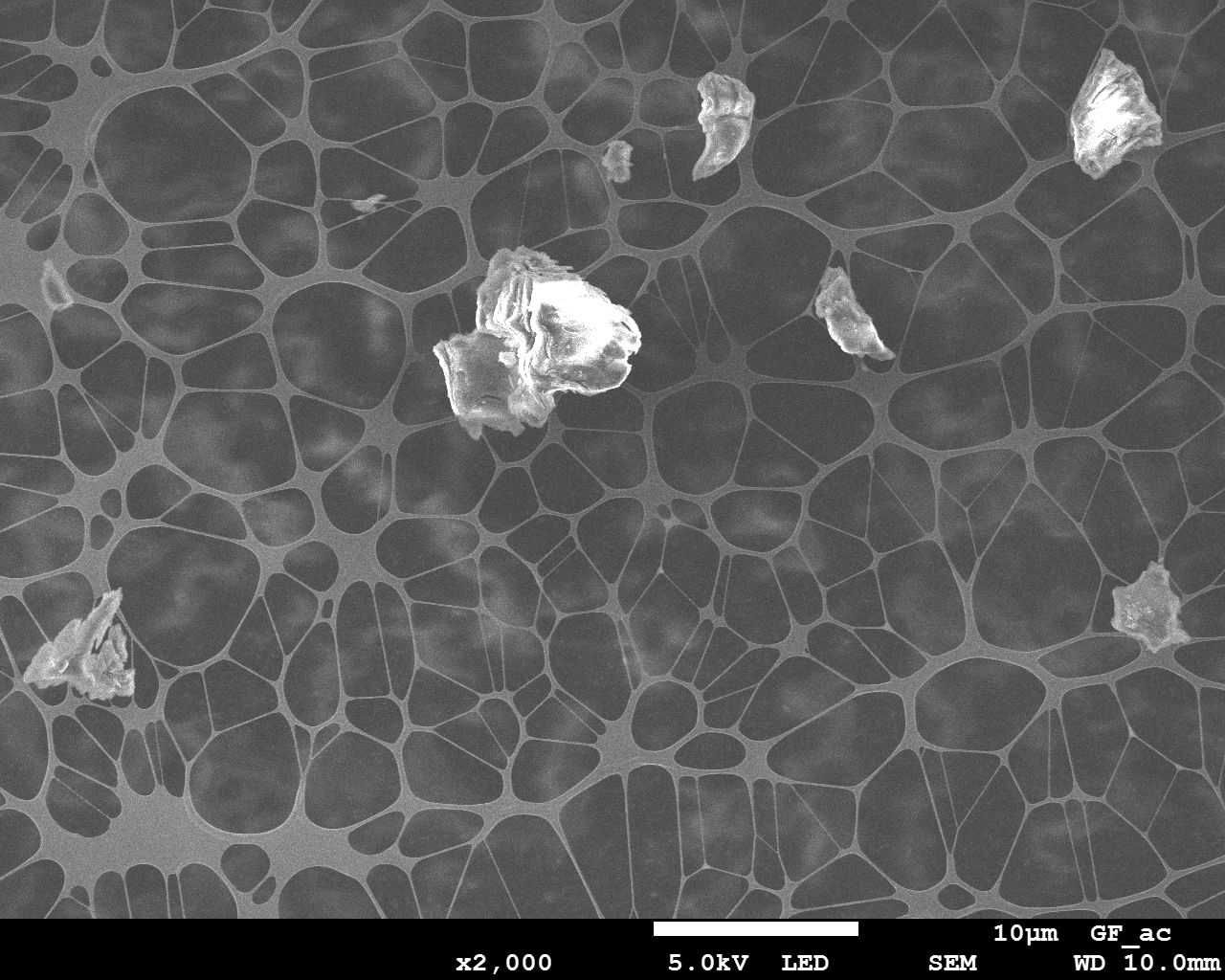

Supplement: Supplementary file 1 — ja3c13296_si_001.zip [file ja3c13296_si_001.zip › Data_archive/SEM/SEM FG size/GF_ac_im023.jpg]

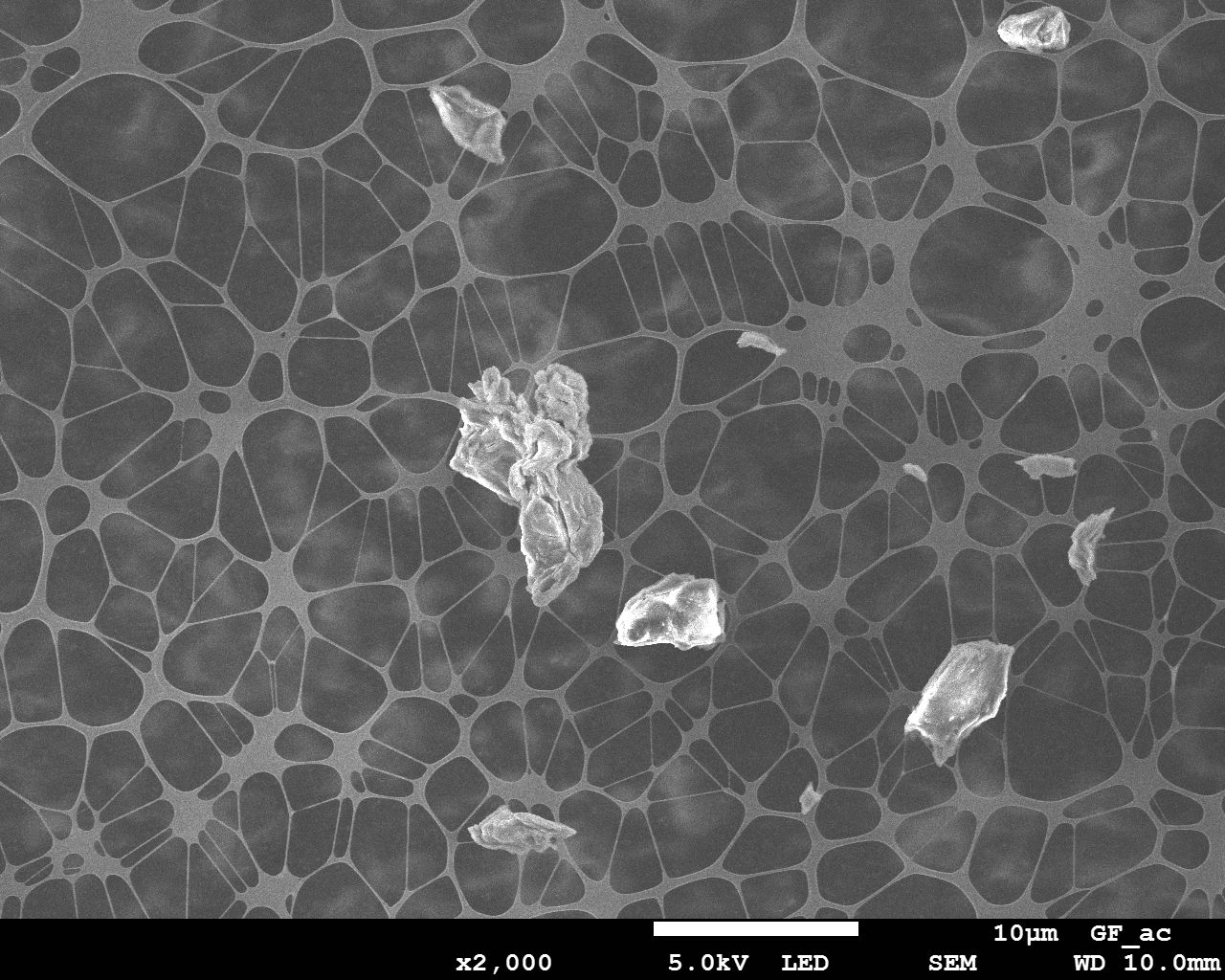

Supplement: Supplementary file 1 — ja3c13296_si_001.zip [file ja3c13296_si_001.zip › Data_archive/SEM/SEM FG size/GF_ac_im024.jpg]

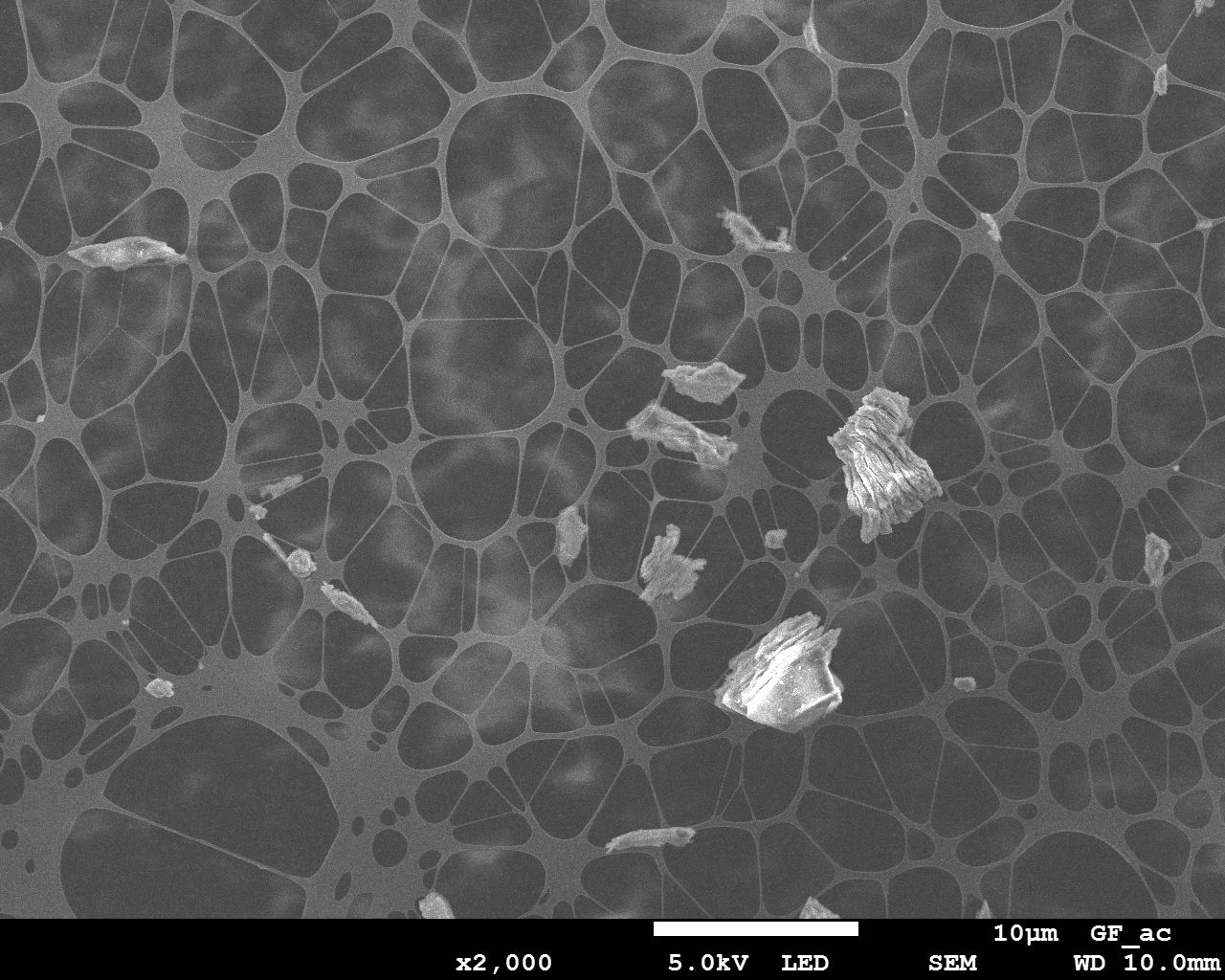

Supplement: Supplementary file 1 — ja3c13296_si_001.zip [file ja3c13296_si_001.zip › Data_archive/SEM/SEM FG size/GF_ac_im025.jpg]

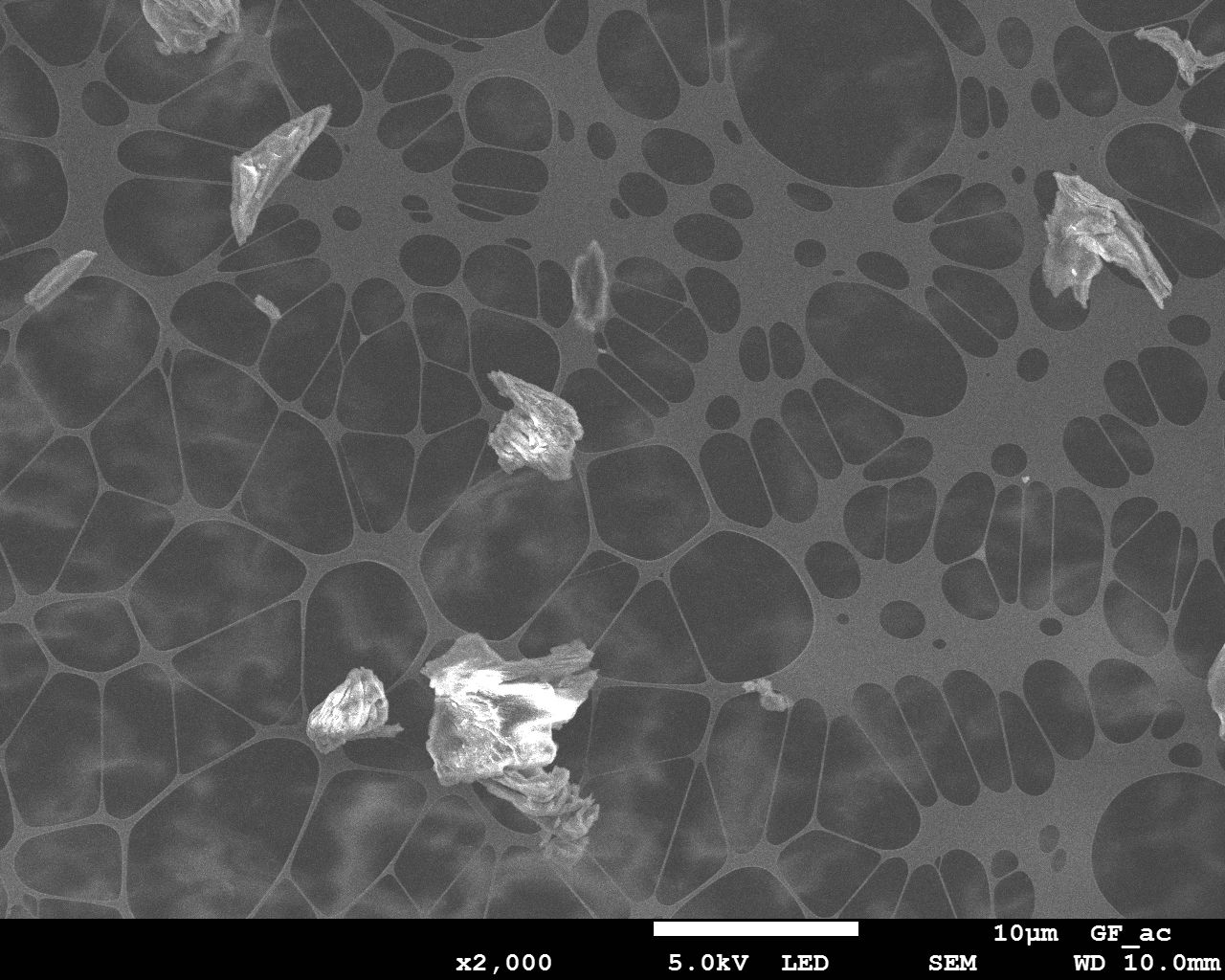

Supplement: Supplementary file 1 — ja3c13296_si_001.zip [file ja3c13296_si_001.zip › Data_archive/SEM/SEM FG size/GF_ac_im026.jpg]

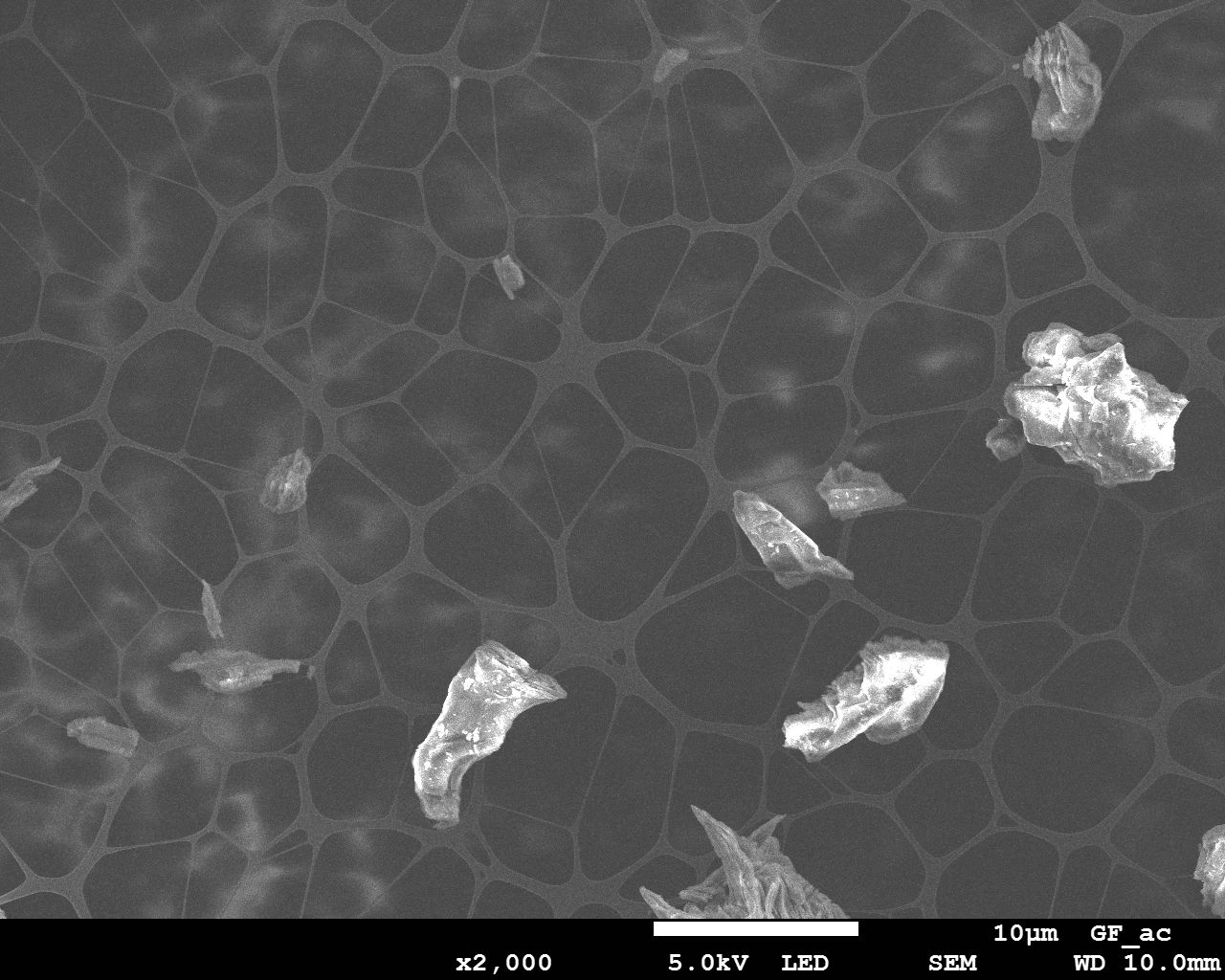

Supplement: Supplementary file 1 — ja3c13296_si_001.zip [file ja3c13296_si_001.zip › Data_archive/SEM/SEM FG size/GF_ac_im027.jpg]

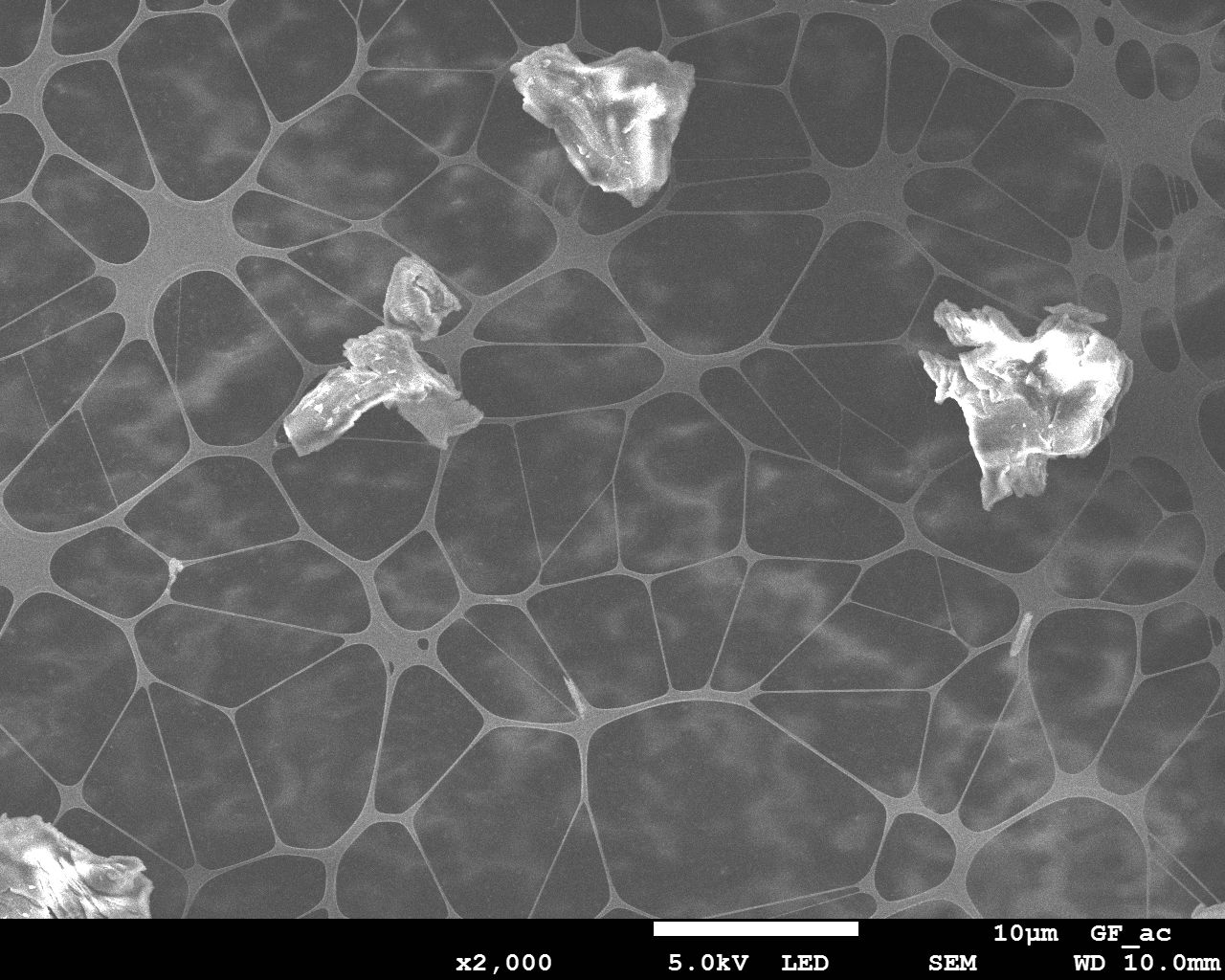

Supplement: Supplementary file 1 — ja3c13296_si_001.zip [file ja3c13296_si_001.zip › Data_archive/SEM/SEM FG size/GF_ac_im028.jpg]

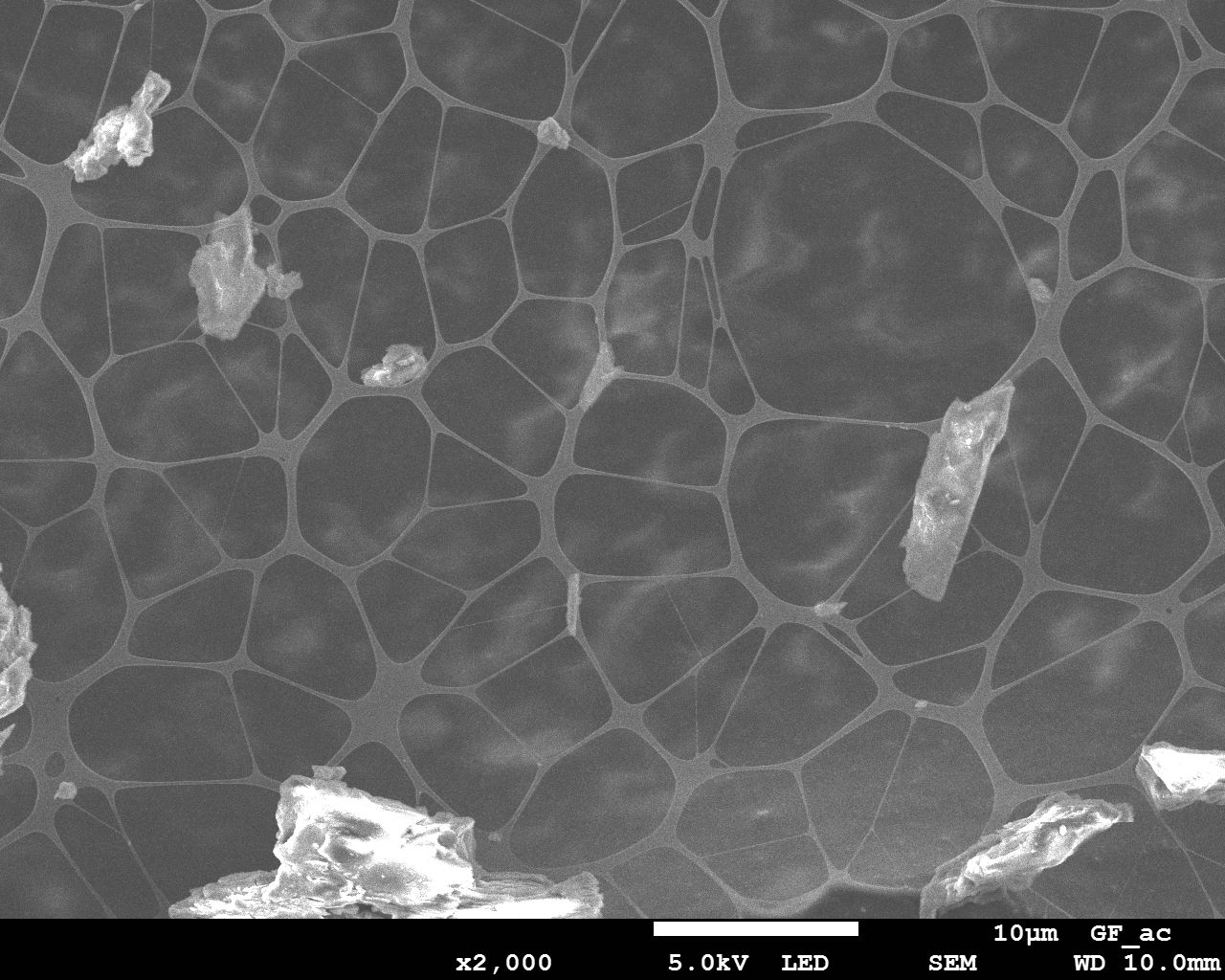

Supplement: Supplementary file 1 — ja3c13296_si_001.zip [file ja3c13296_si_001.zip › Data_archive/SEM/SEM FG size/GF_ac_im029.jpg]

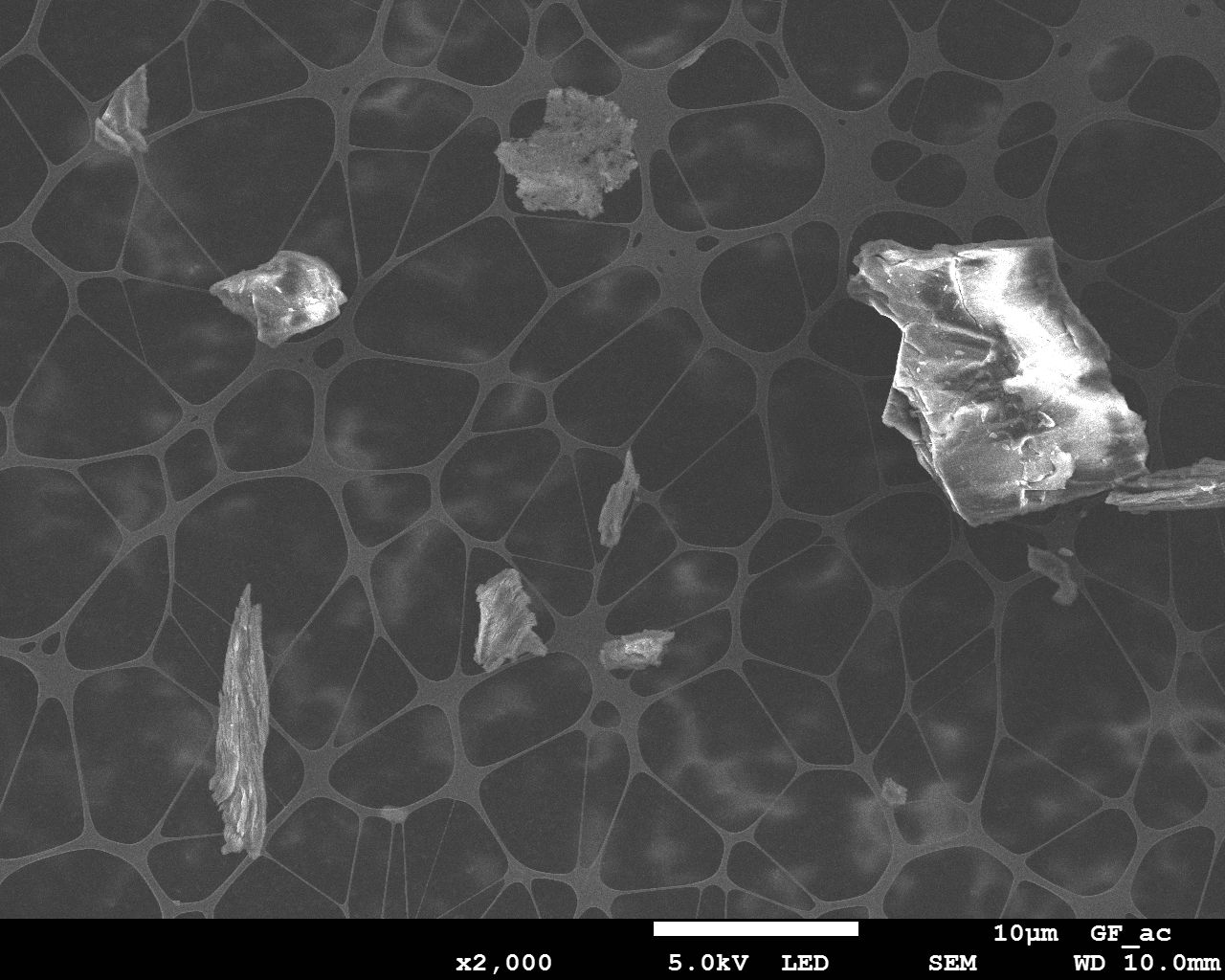

Supplement: Supplementary file 1 — ja3c13296_si_001.zip [file ja3c13296_si_001.zip › Data_archive/SEM/SEM FG size/GF_ac_im030.jpg]

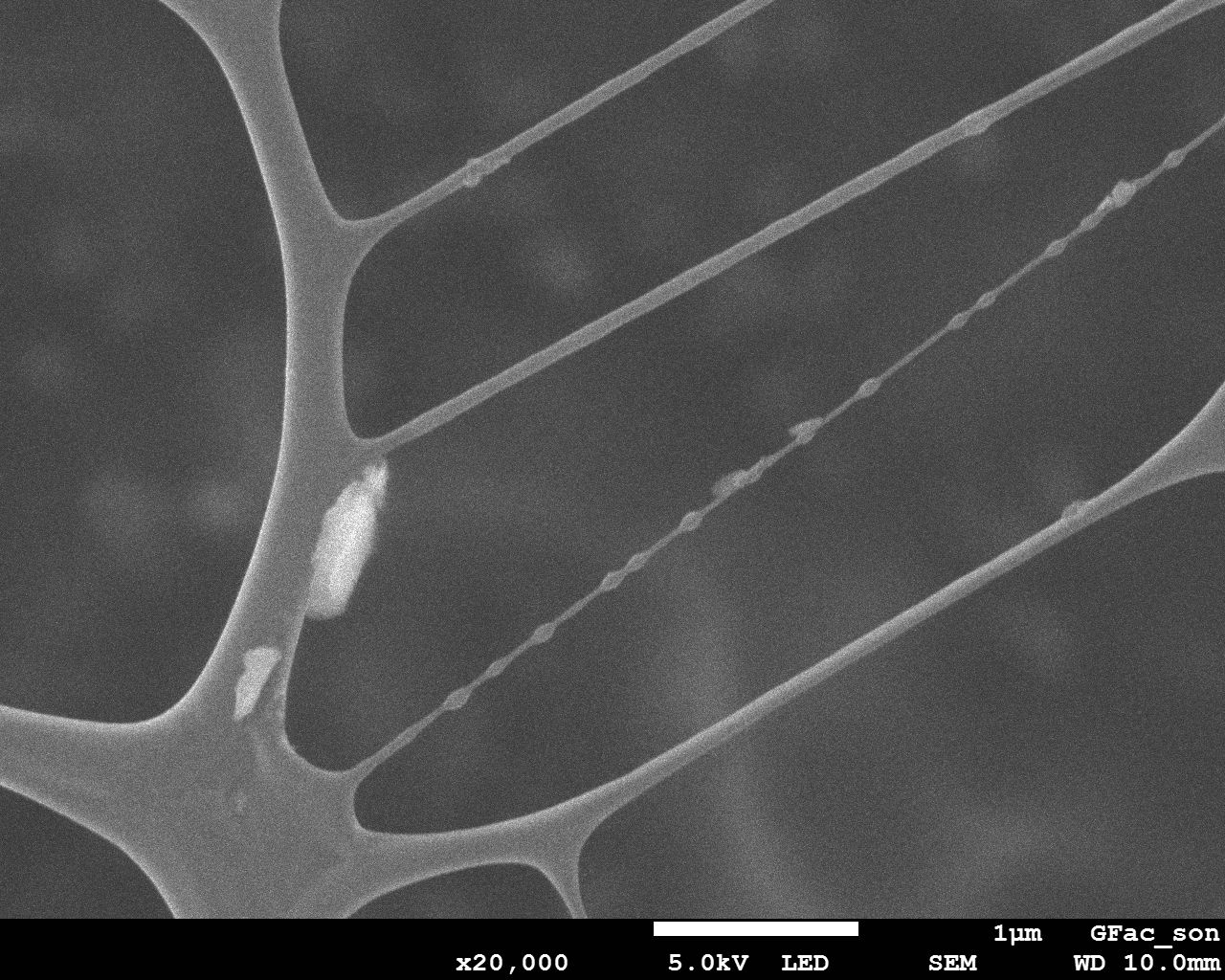

Supplement: Supplementary file 1 — ja3c13296_si_001.zip [file ja3c13296_si_001.zip › Data_archive/SEM/SEM small FG size/GF_ac_son_im001.jpg]

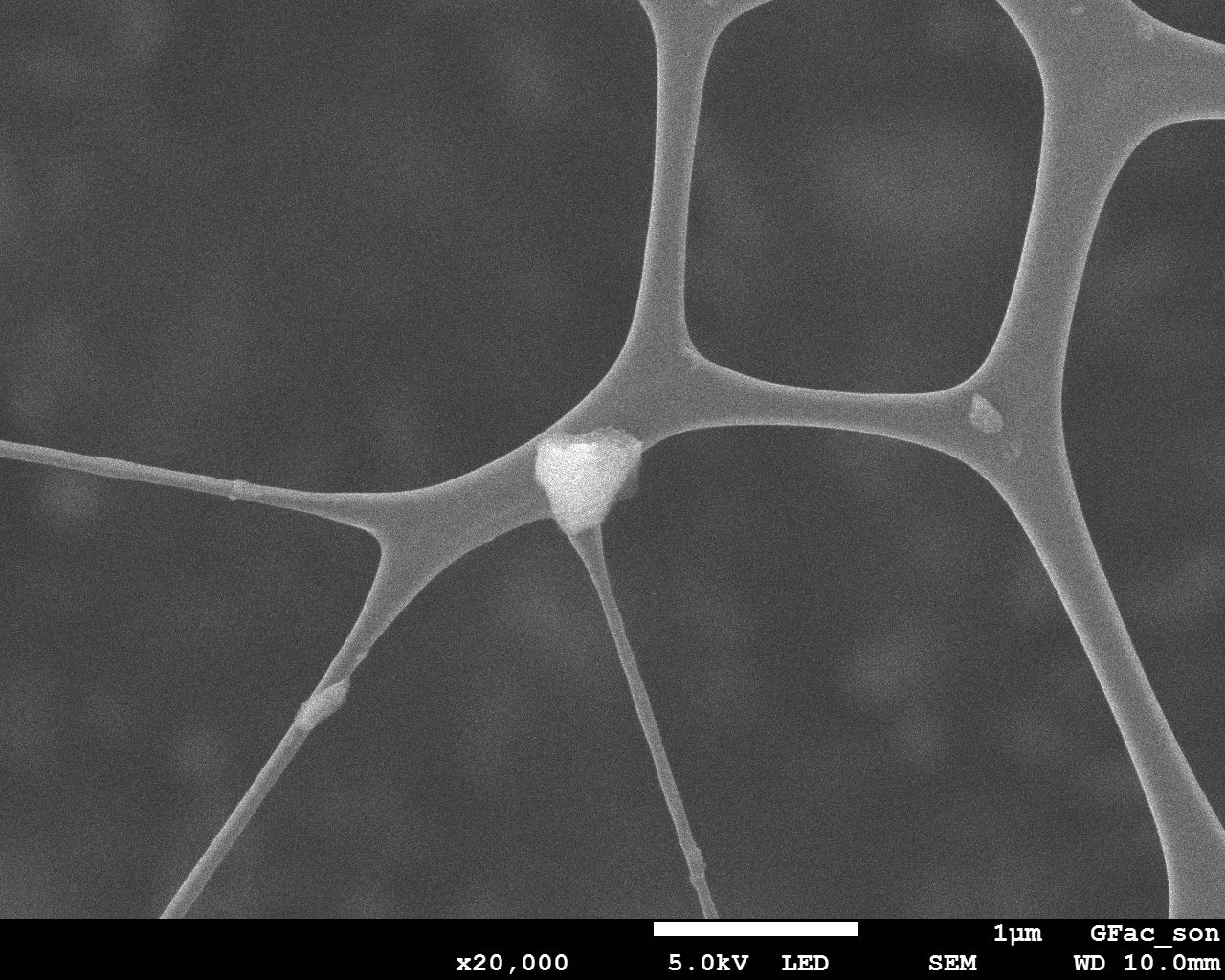

Supplement: Supplementary file 1 — ja3c13296_si_001.zip [file ja3c13296_si_001.zip › Data_archive/SEM/SEM small FG size/GF_ac_son_im003.jpg]

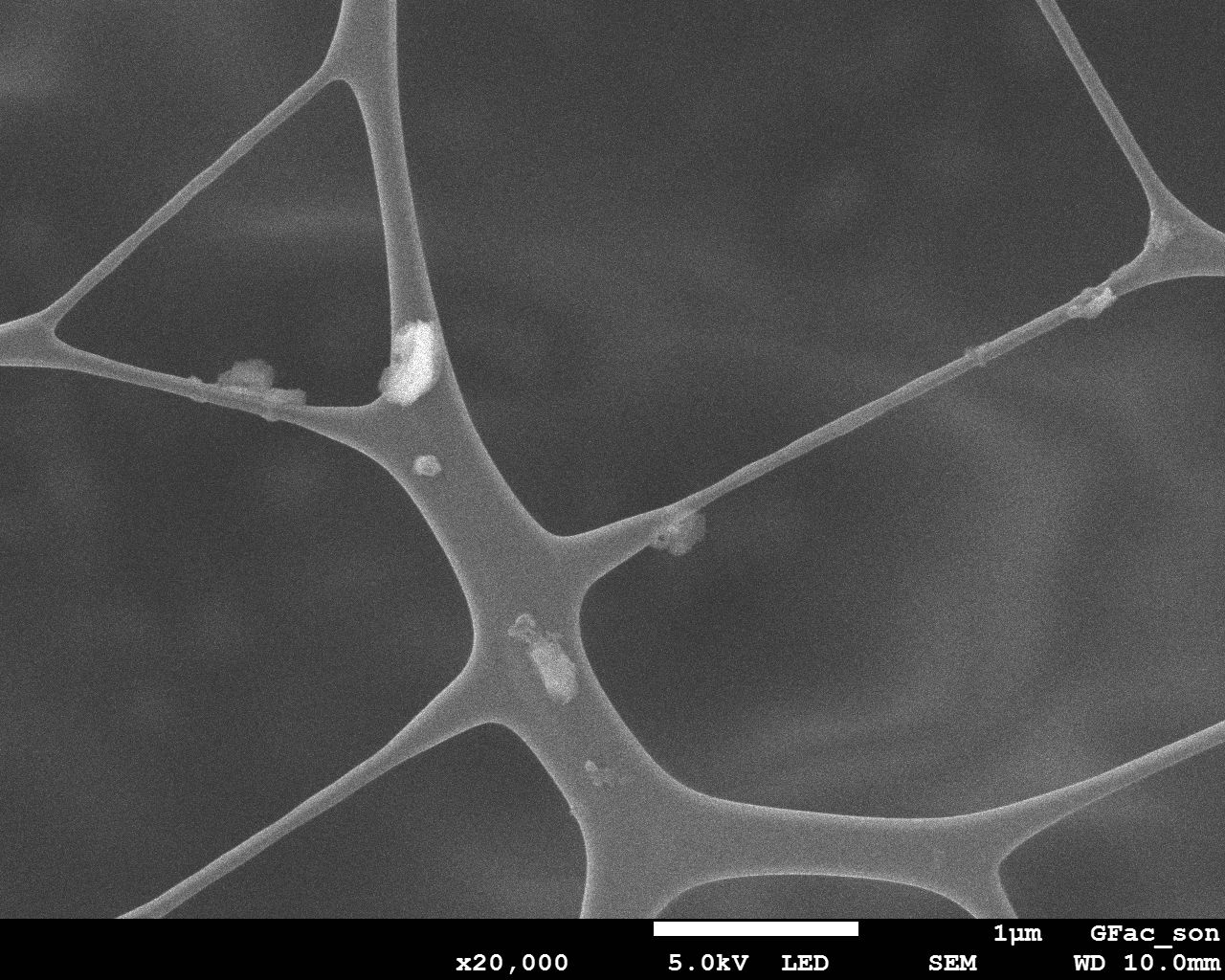

Supplement: Supplementary file 1 — ja3c13296_si_001.zip [file ja3c13296_si_001.zip › Data_archive/SEM/SEM small FG size/GF_ac_son_im004.jpg]

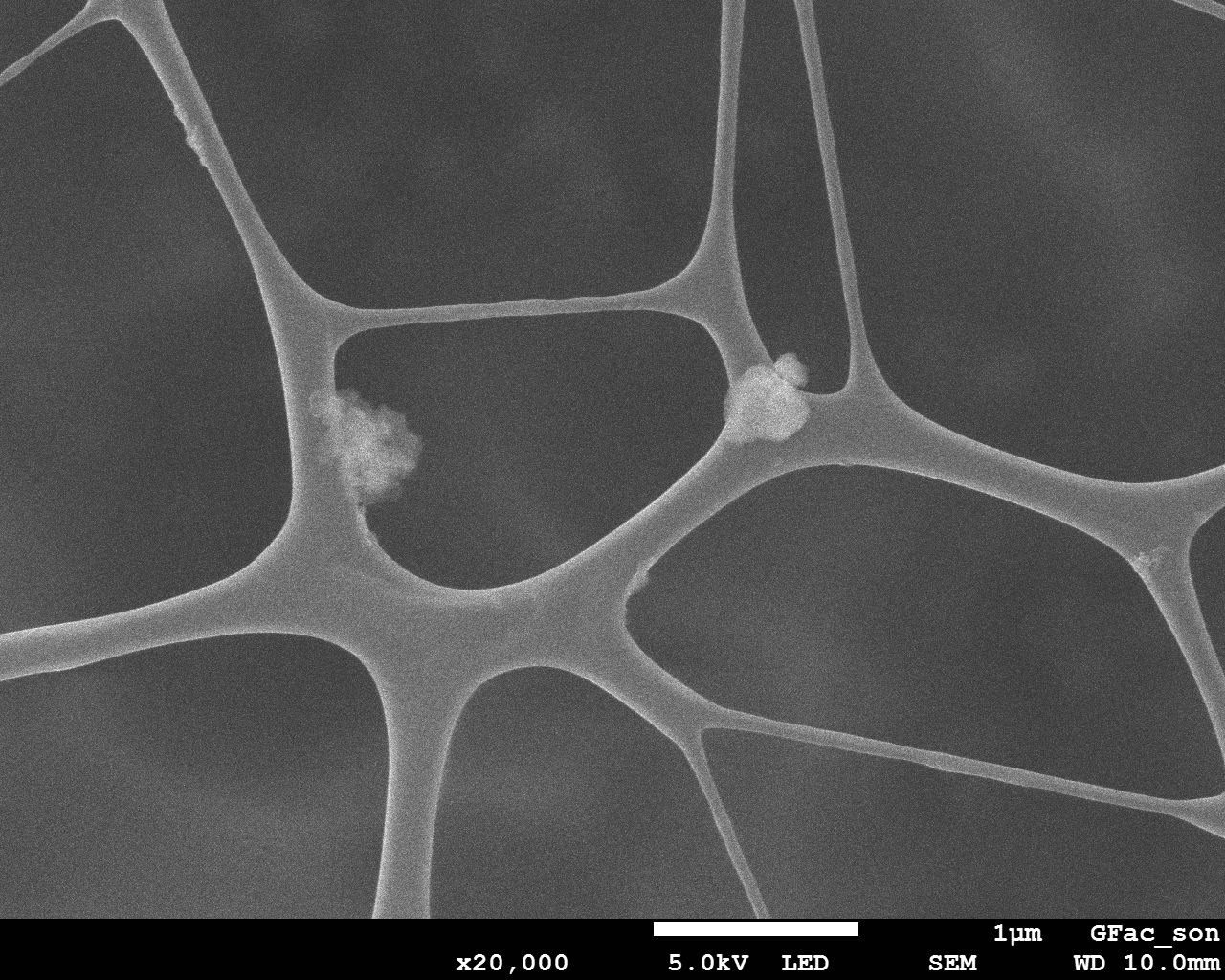

Supplement: Supplementary file 1 — ja3c13296_si_001.zip [file ja3c13296_si_001.zip › Data_archive/SEM/SEM small FG size/GF_ac_son_im006.jpg]

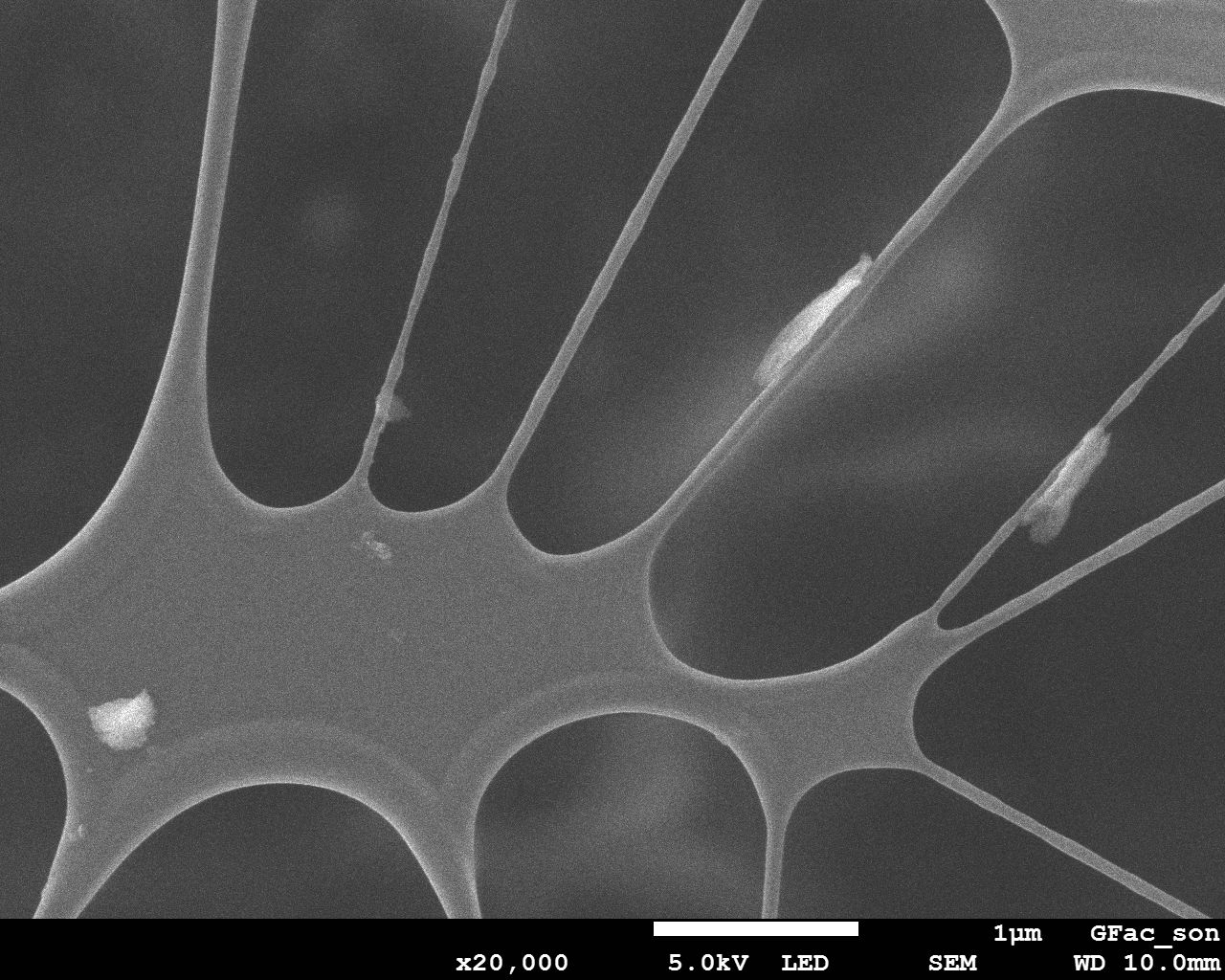

Supplement: Supplementary file 1 — ja3c13296_si_001.zip [file ja3c13296_si_001.zip › Data_archive/SEM/SEM small FG size/GF_ac_son_im007.jpg]

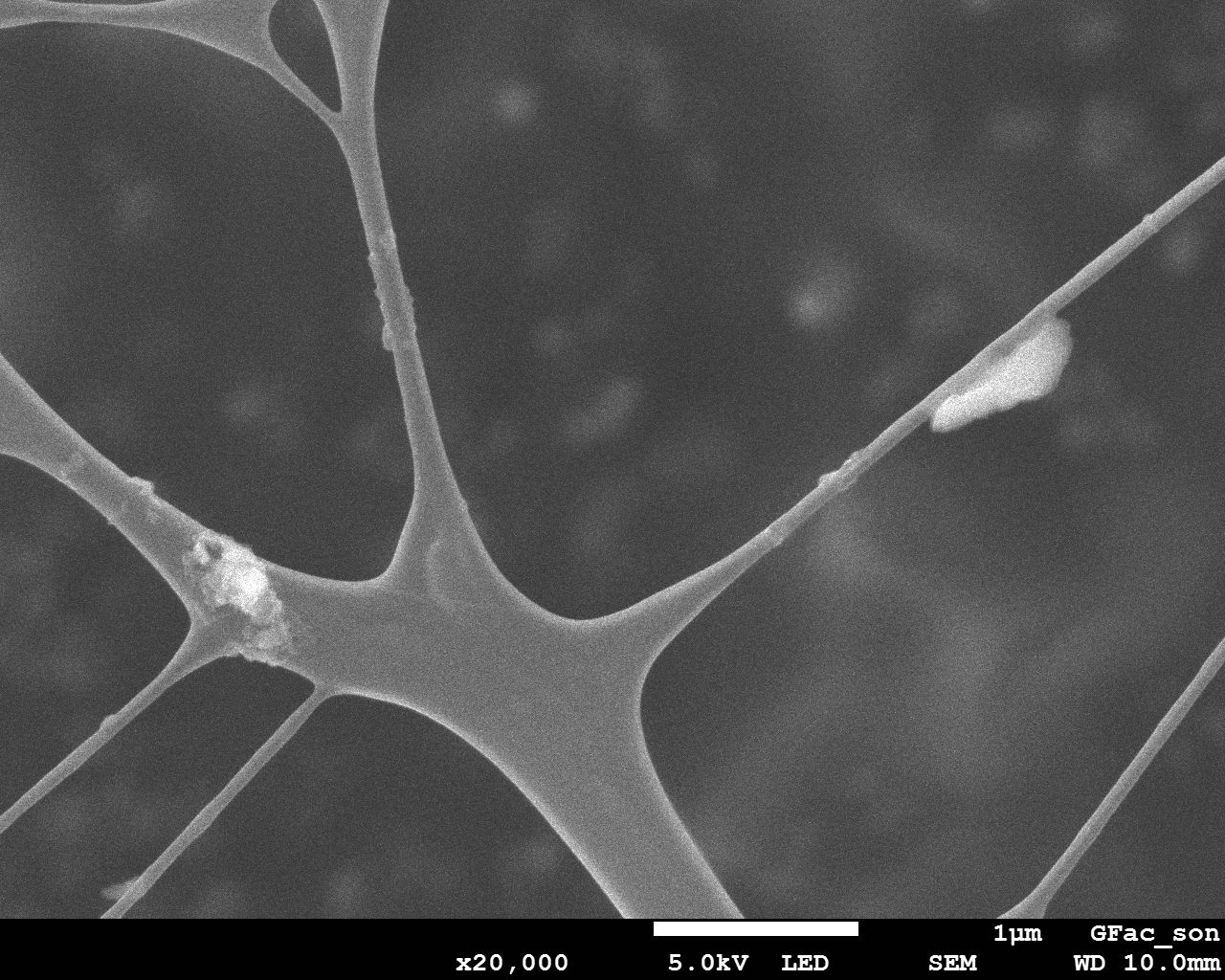

Supplement: Supplementary file 1 — ja3c13296_si_001.zip [file ja3c13296_si_001.zip › Data_archive/SEM/SEM small FG size/GF_ac_son_im008.jpg]

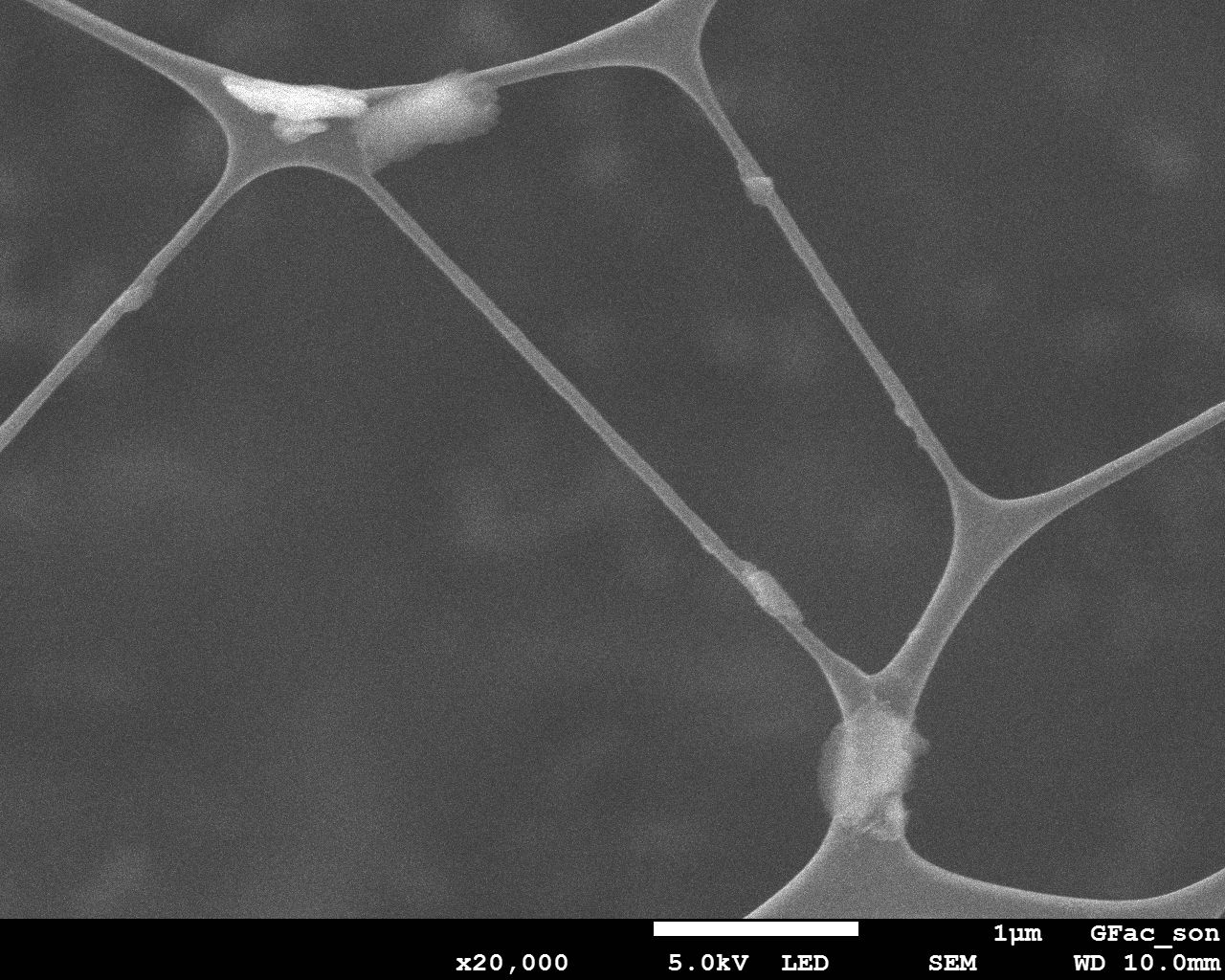

Supplement: Supplementary file 1 — ja3c13296_si_001.zip [file ja3c13296_si_001.zip › Data_archive/SEM/SEM small FG size/GF_ac_son_im010.jpg]

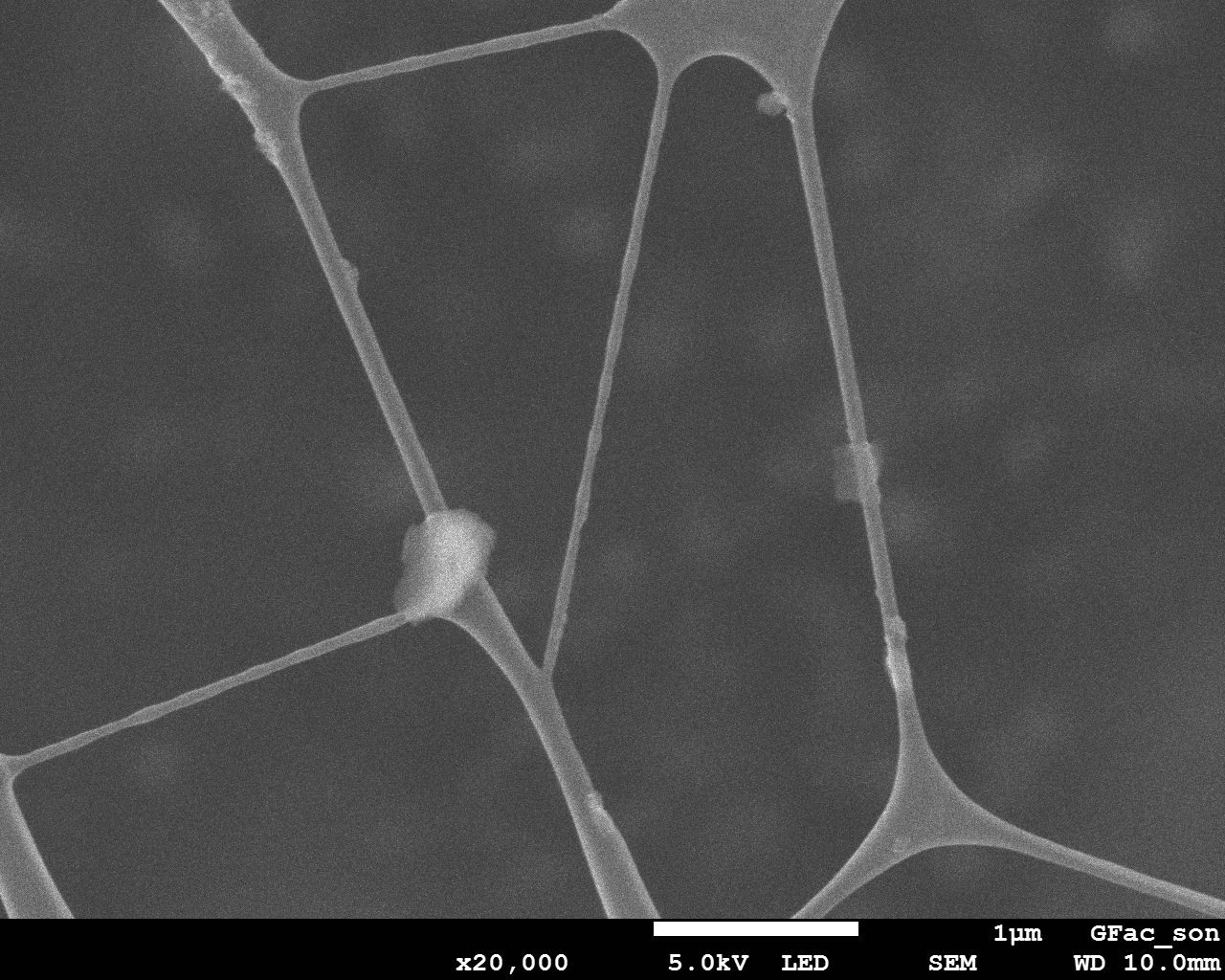

Supplement: Supplementary file 1 — ja3c13296_si_001.zip [file ja3c13296_si_001.zip › Data_archive/SEM/SEM small FG size/GF_ac_son_im011.jpg]

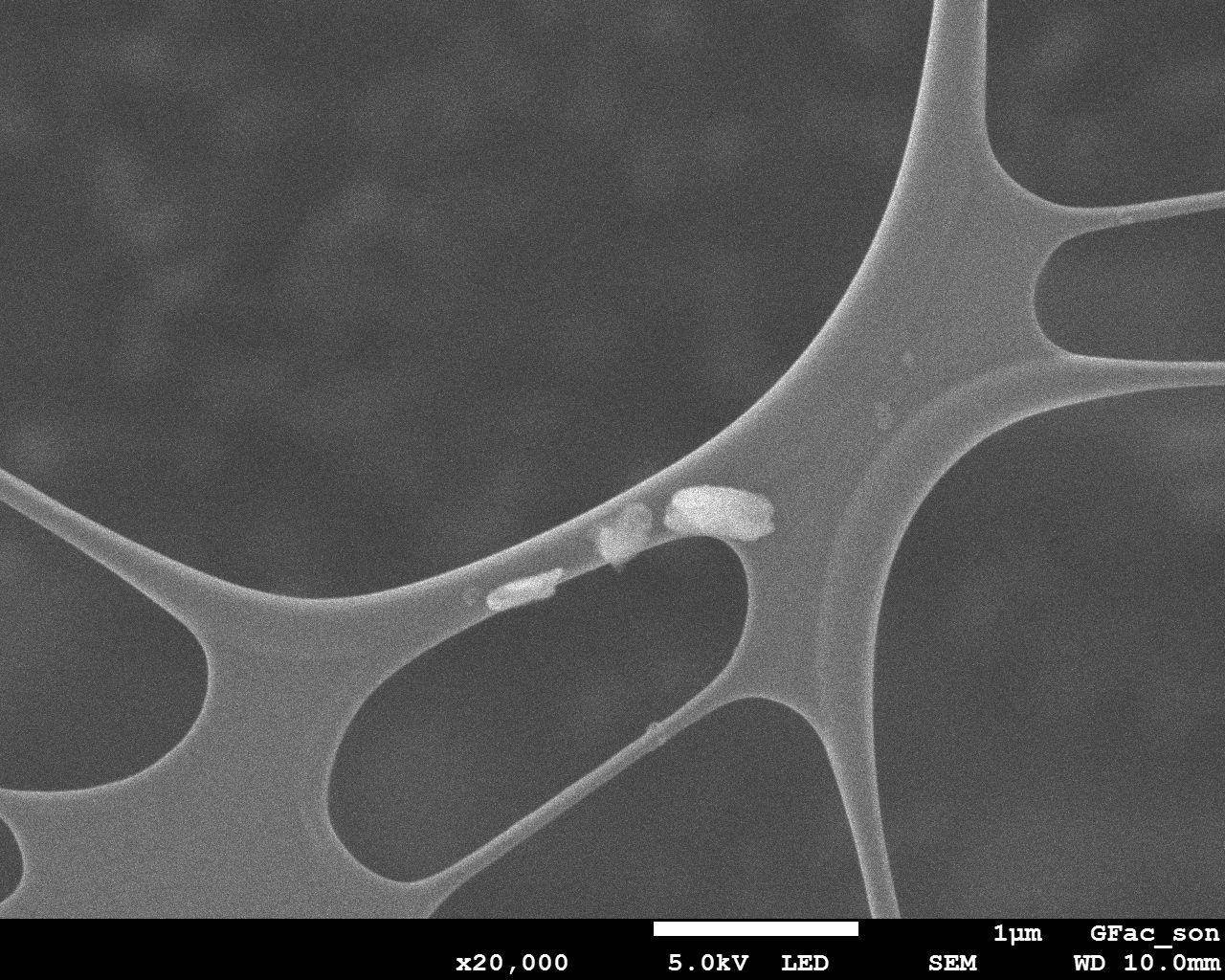

Supplement: Supplementary file 1 — ja3c13296_si_001.zip [file ja3c13296_si_001.zip › Data_archive/SEM/SEM small FG size/GF_ac_son_im012.jpg]

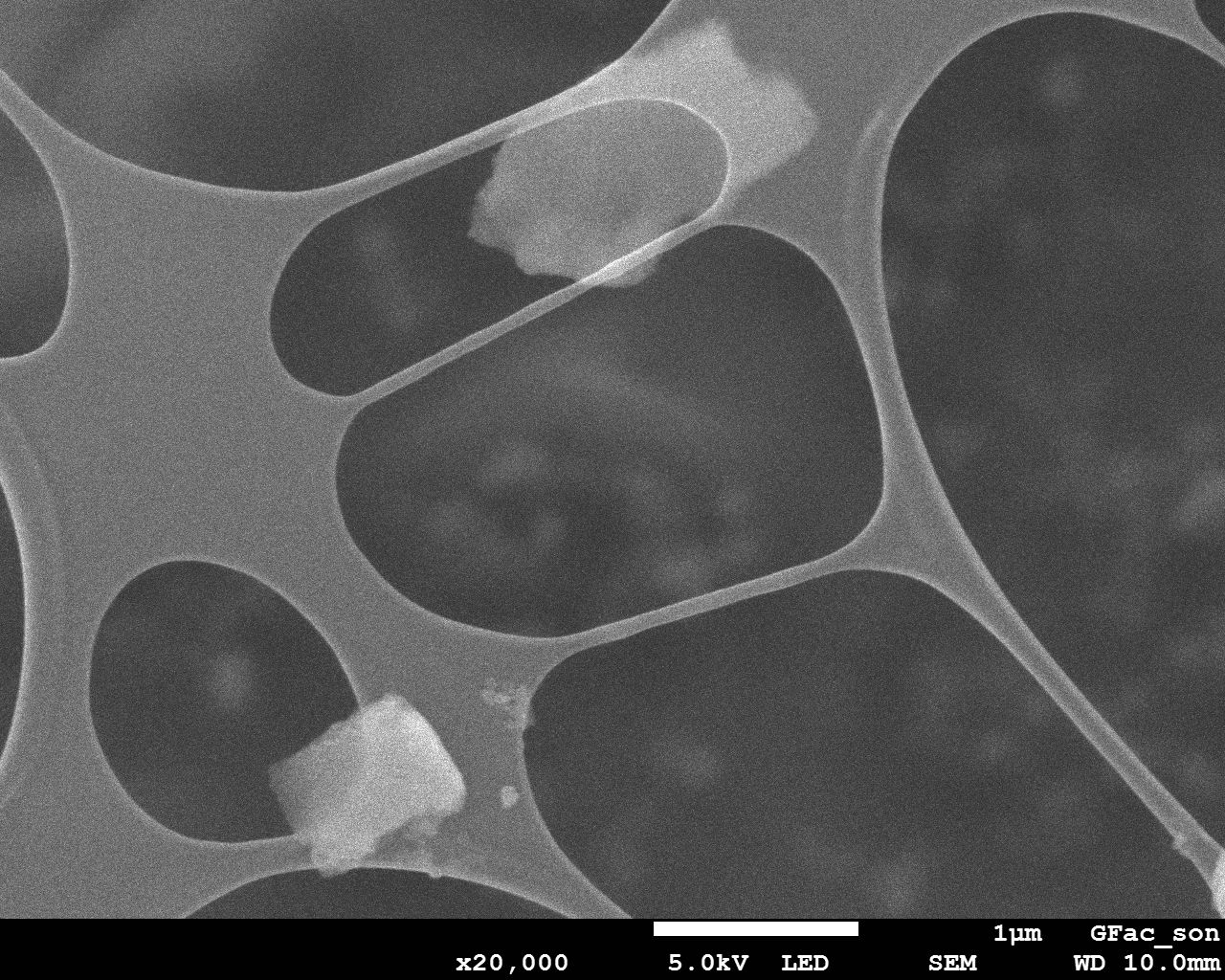

Supplement: Supplementary file 1 — ja3c13296_si_001.zip [file ja3c13296_si_001.zip › Data_archive/SEM/SEM small FG size/GF_ac_son_im013.jpg]

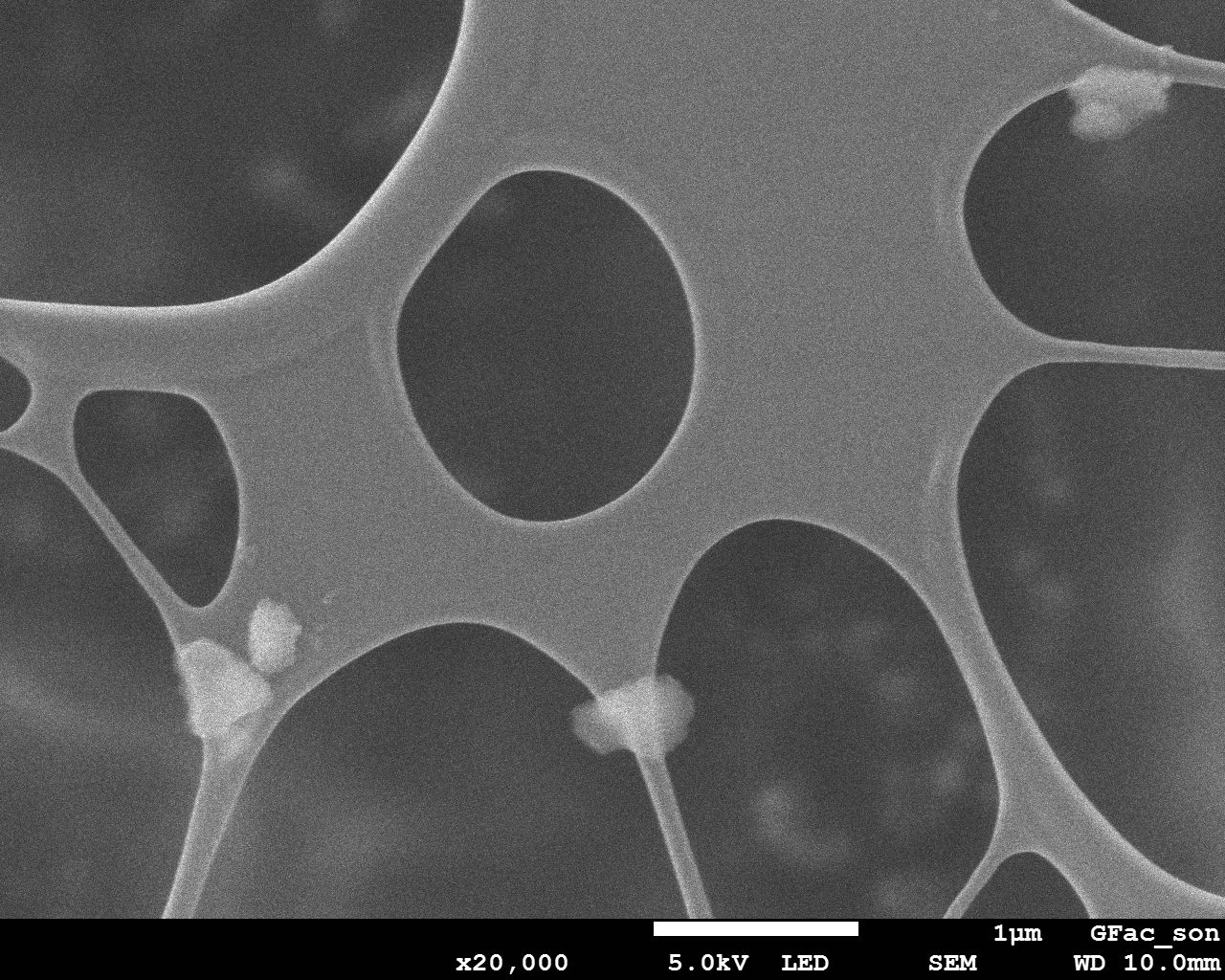

Supplement: Supplementary file 1 — ja3c13296_si_001.zip [file ja3c13296_si_001.zip › Data_archive/SEM/SEM small FG size/GF_ac_son_im014.jpg]

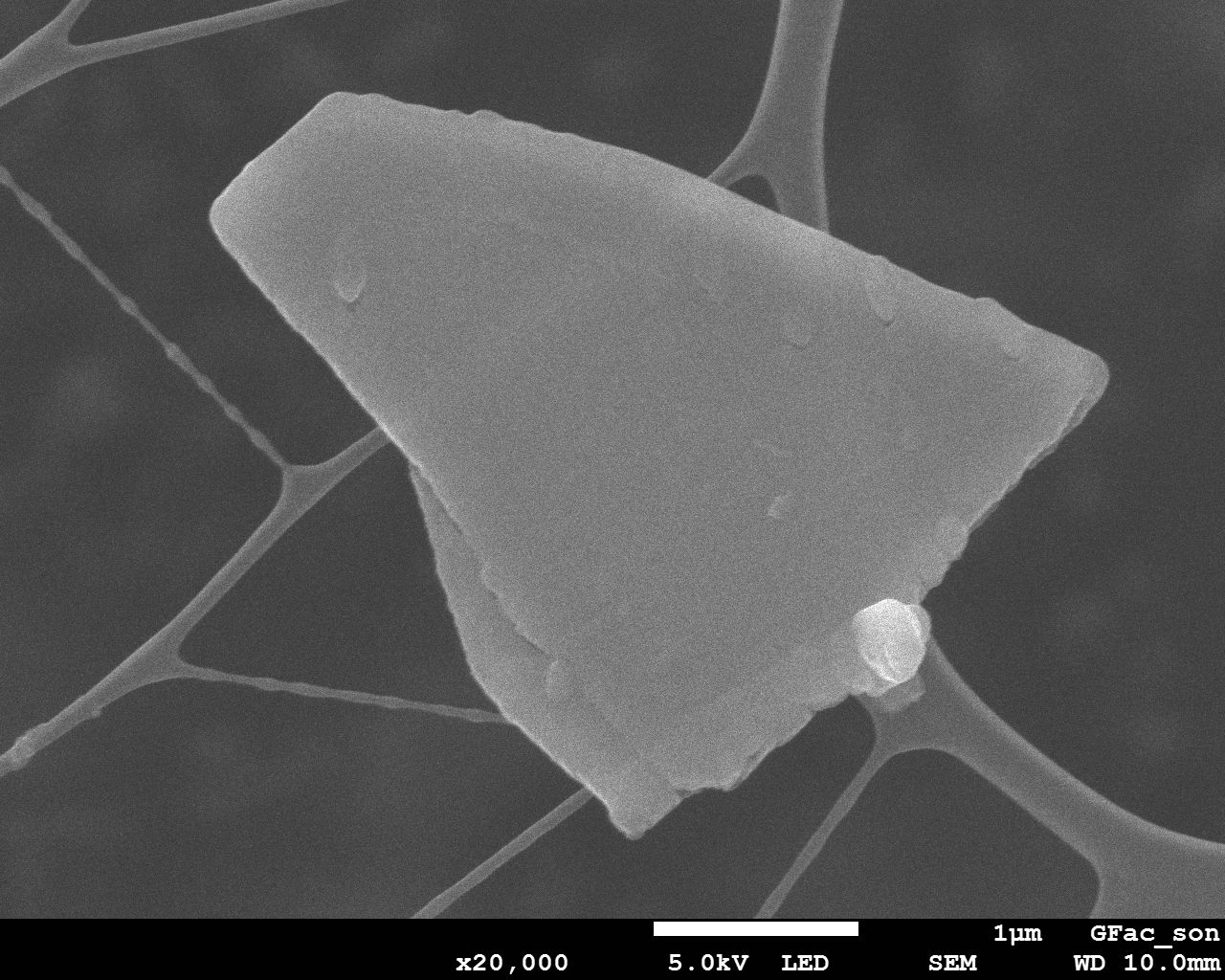

Supplement: Supplementary file 1 — ja3c13296_si_001.zip [file ja3c13296_si_001.zip › Data_archive/SEM/SEM small FG size/GF_ac_son_im015.jpg]

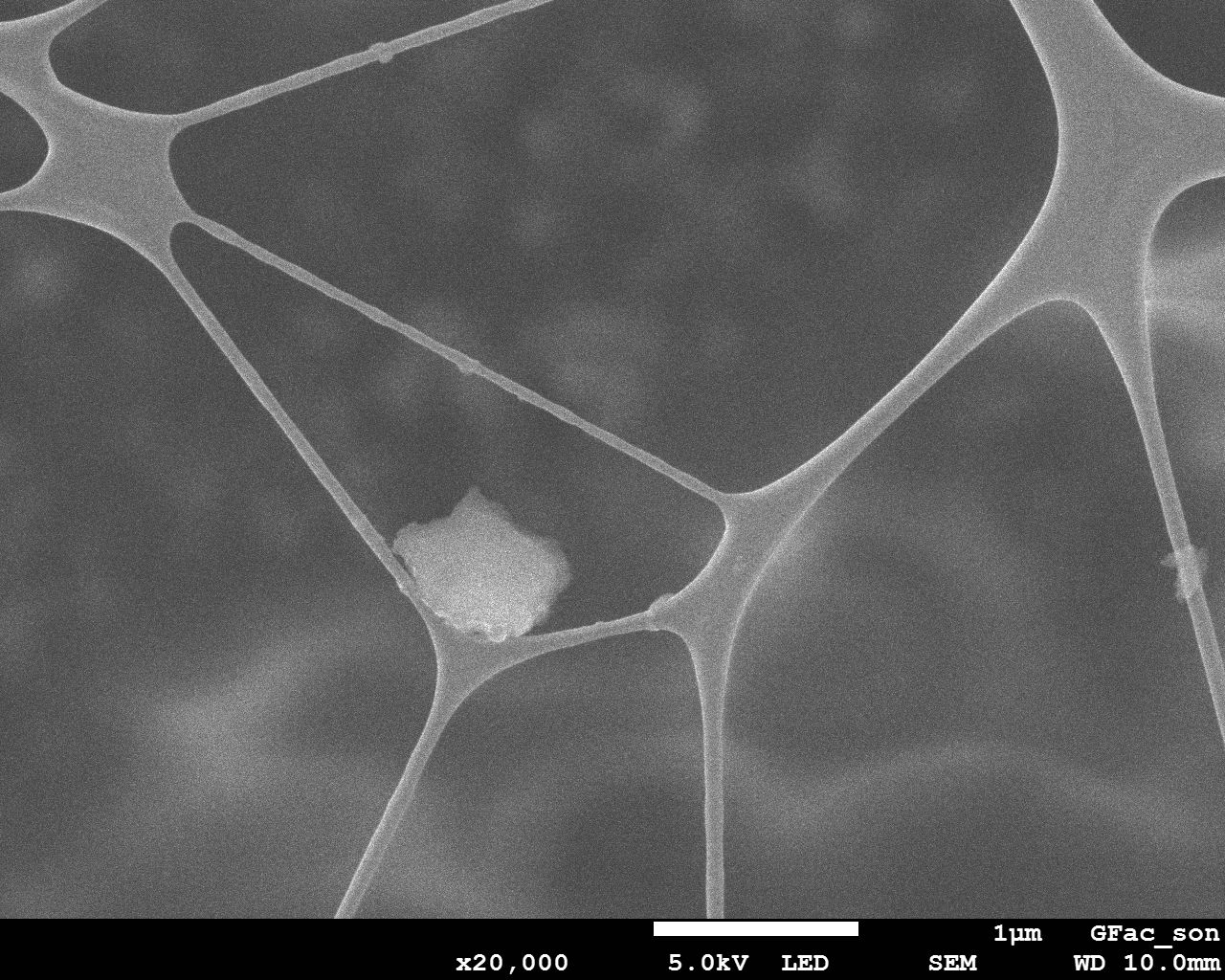

Supplement: Supplementary file 1 — ja3c13296_si_001.zip [file ja3c13296_si_001.zip › Data_archive/SEM/SEM small FG size/GF_ac_son_im016.jpg]

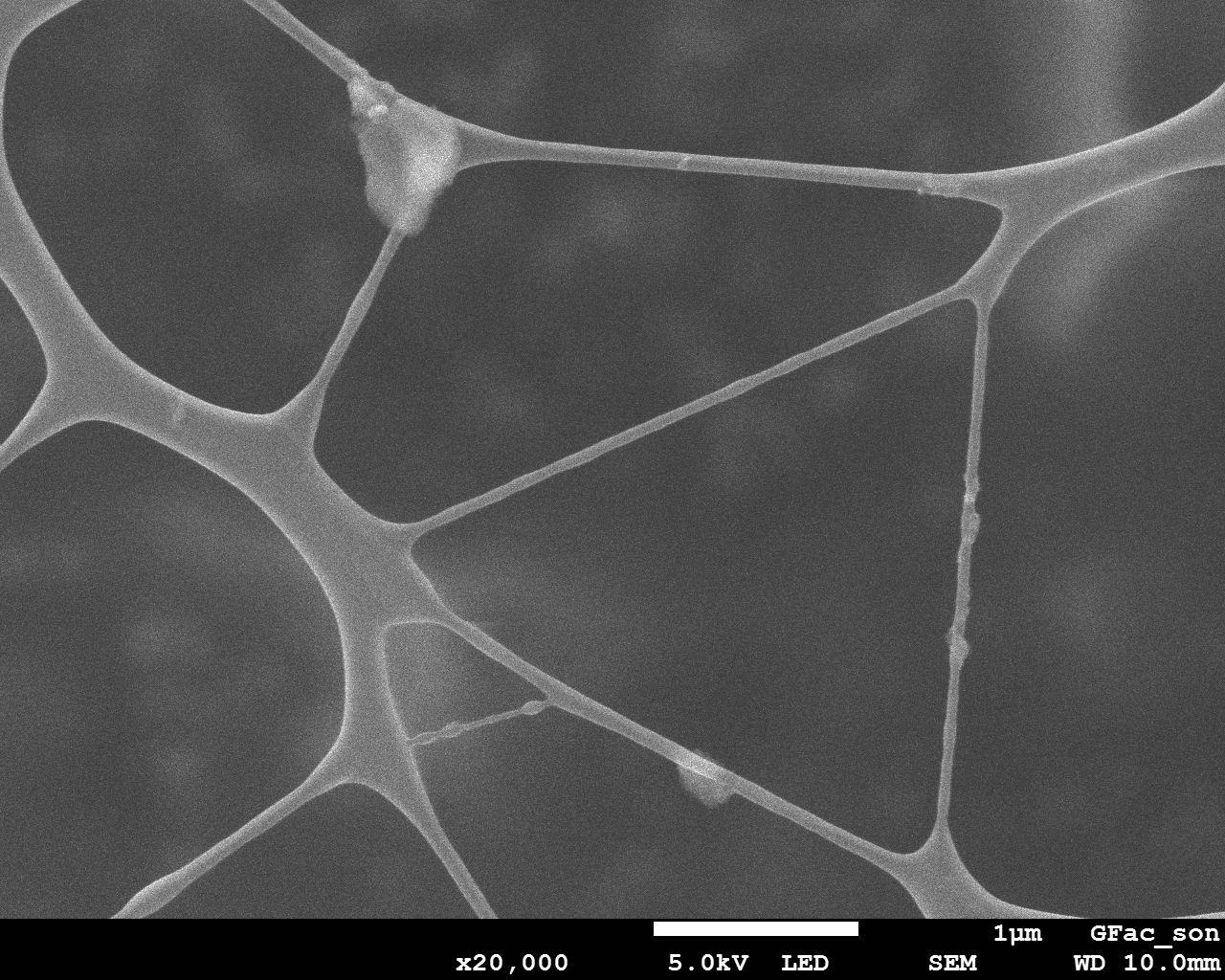

Supplement: Supplementary file 1 — ja3c13296_si_001.zip [file ja3c13296_si_001.zip › Data_archive/SEM/SEM small FG size/GF_ac_son_im017.jpg]

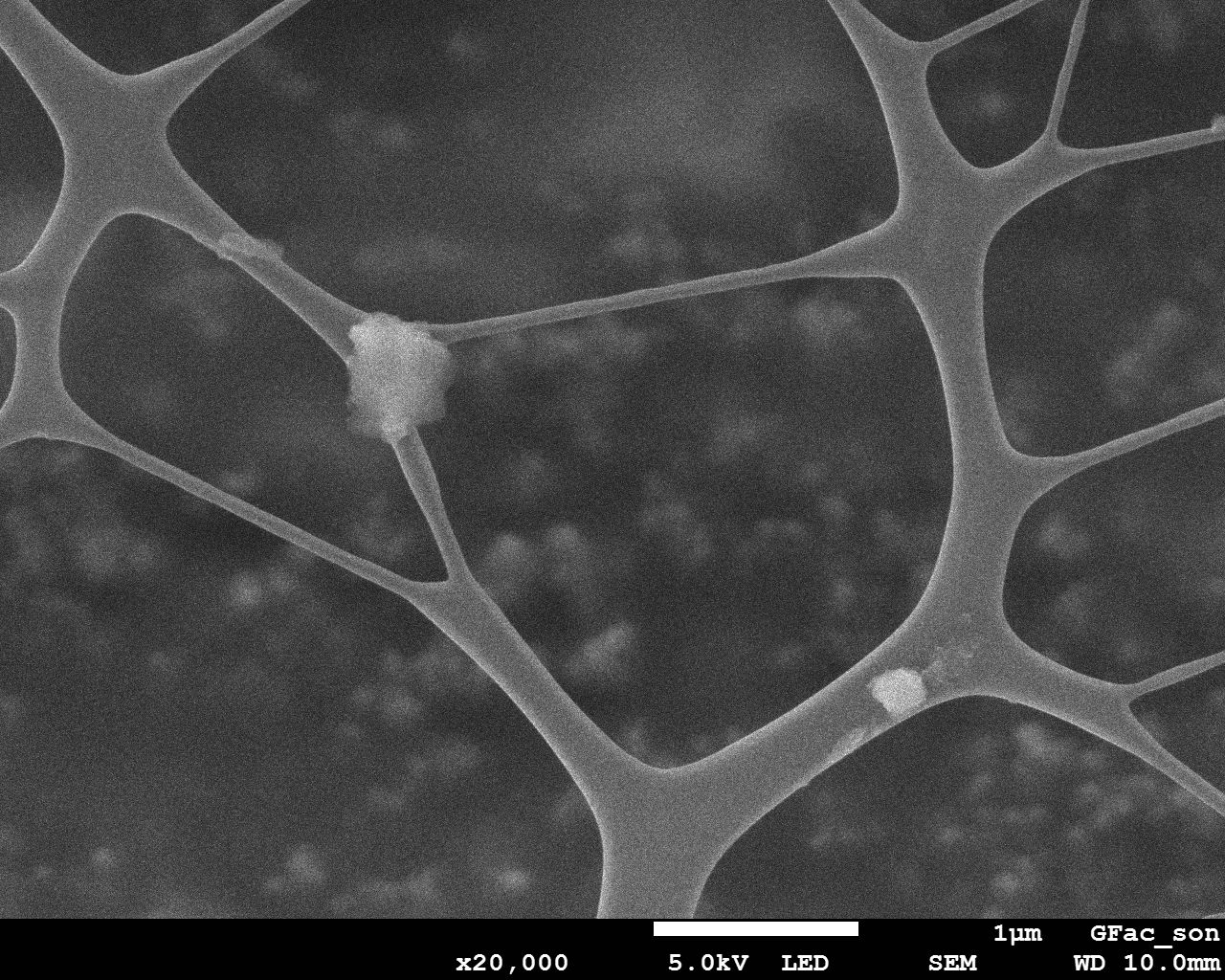

Supplement: Supplementary file 1 — ja3c13296_si_001.zip [file ja3c13296_si_001.zip › Data_archive/SEM/SEM small FG size/GF_ac_son_im018.jpg]

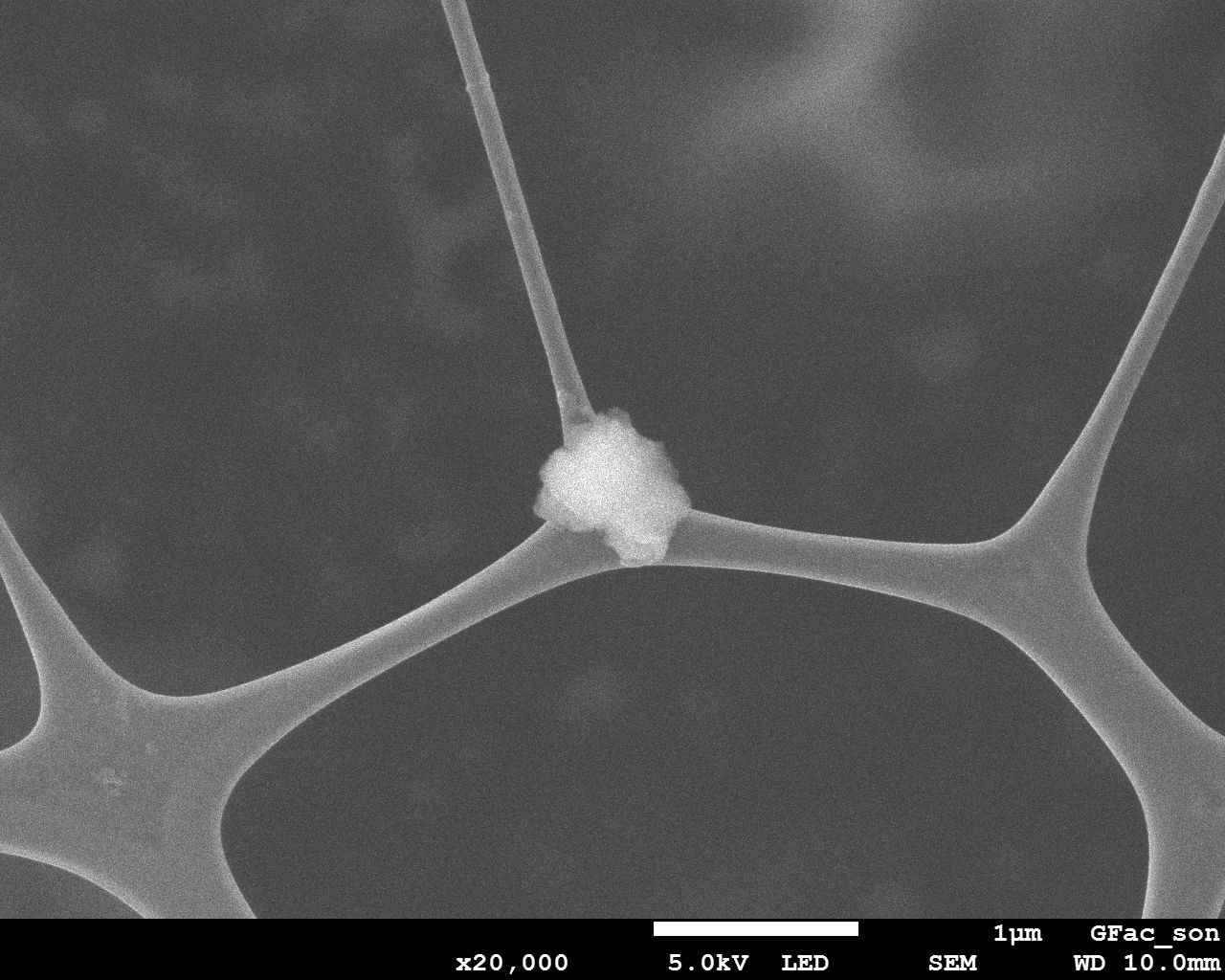

Supplement: Supplementary file 1 — ja3c13296_si_001.zip [file ja3c13296_si_001.zip › Data_archive/SEM/SEM small FG size/GF_ac_son_im019.jpg]

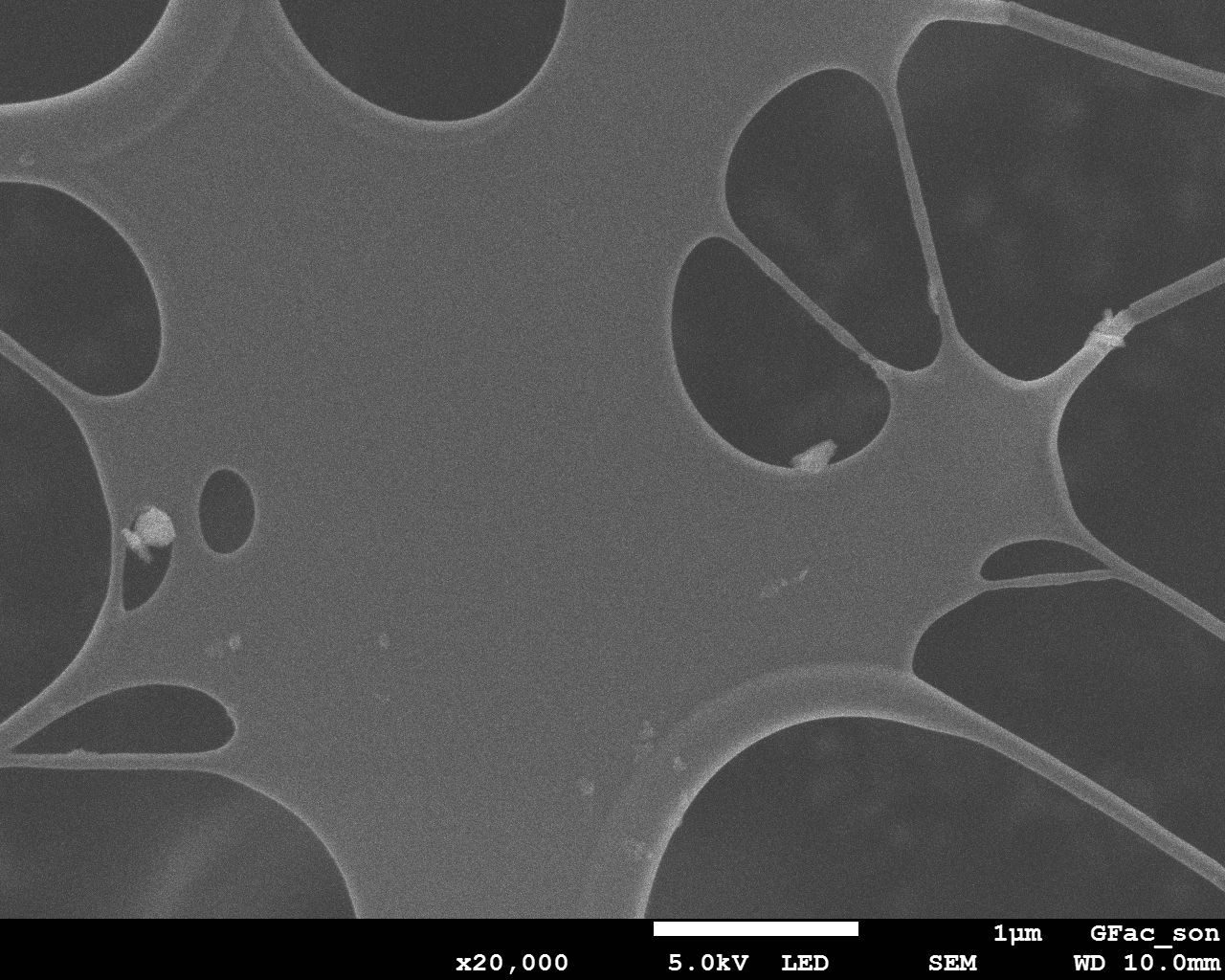

Supplement: Supplementary file 1 — ja3c13296_si_001.zip [file ja3c13296_si_001.zip › Data_archive/SEM/SEM small FG size/GF_ac_son_im020.jpg]

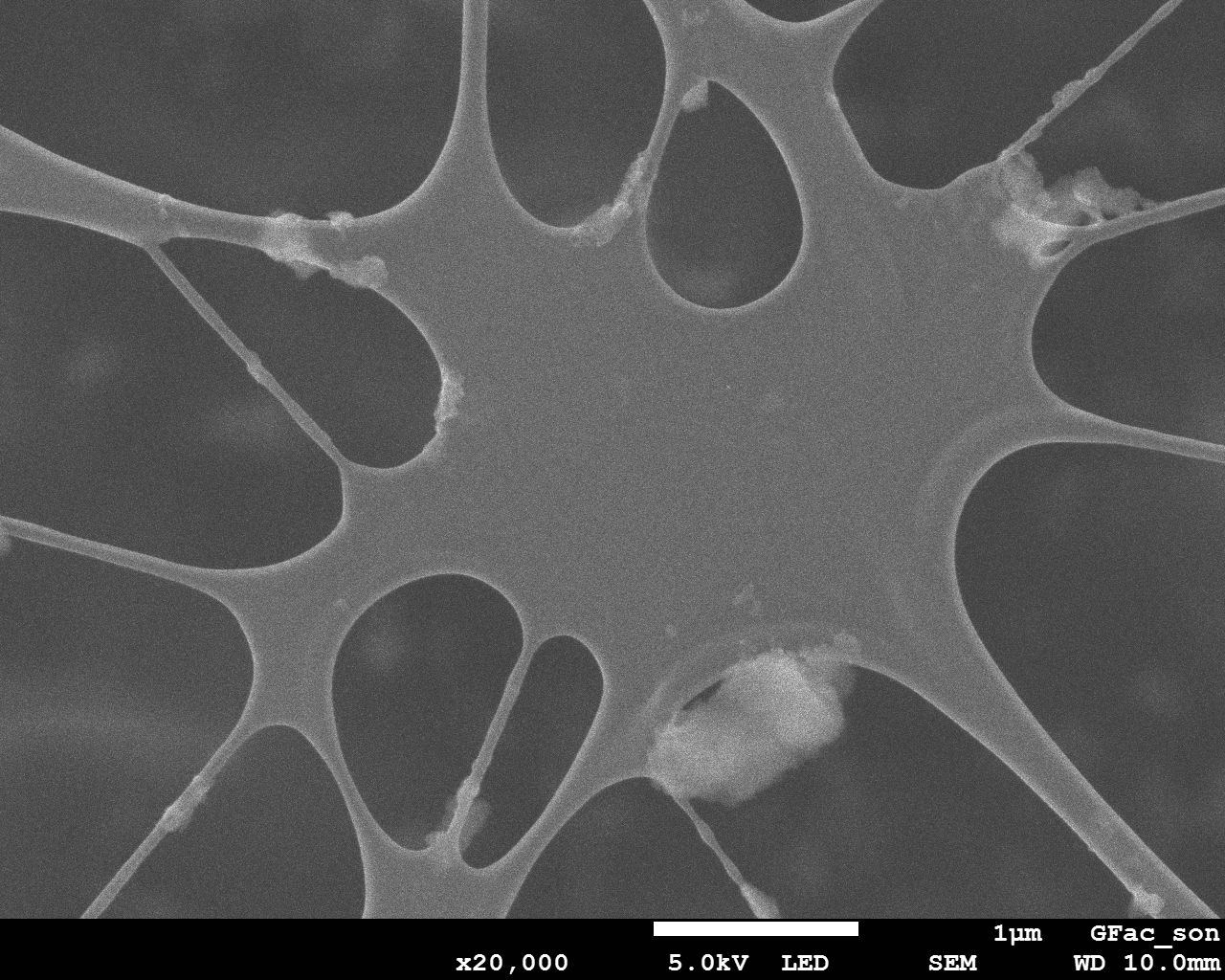

Supplement: Supplementary file 1 — ja3c13296_si_001.zip [file ja3c13296_si_001.zip › Data_archive/SEM/SEM small FG size/GF_ac_son_im021.jpg]

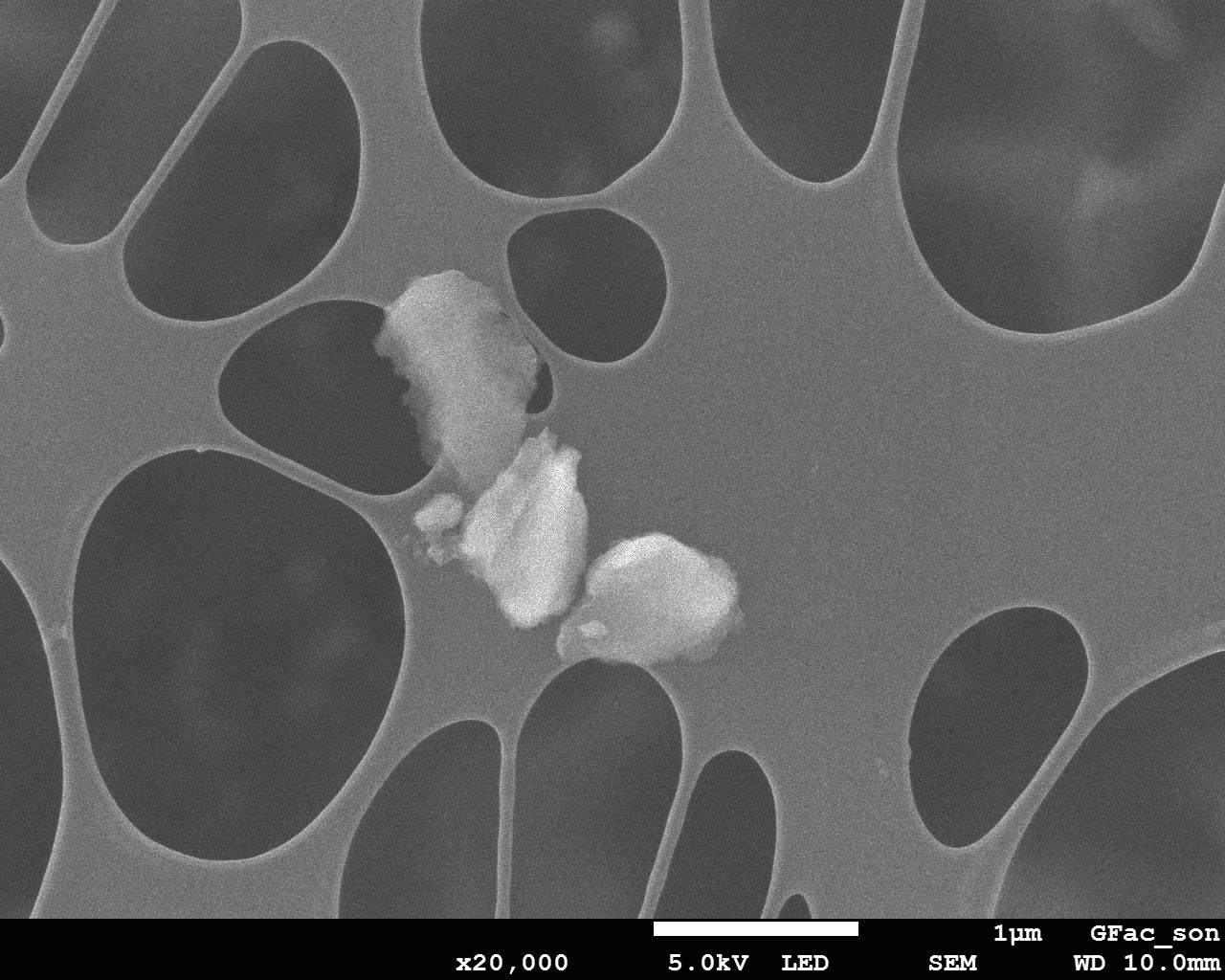

Supplement: Supplementary file 1 — ja3c13296_si_001.zip [file ja3c13296_si_001.zip › Data_archive/SEM/SEM small FG size/GF_ac_son_im022.jpg]

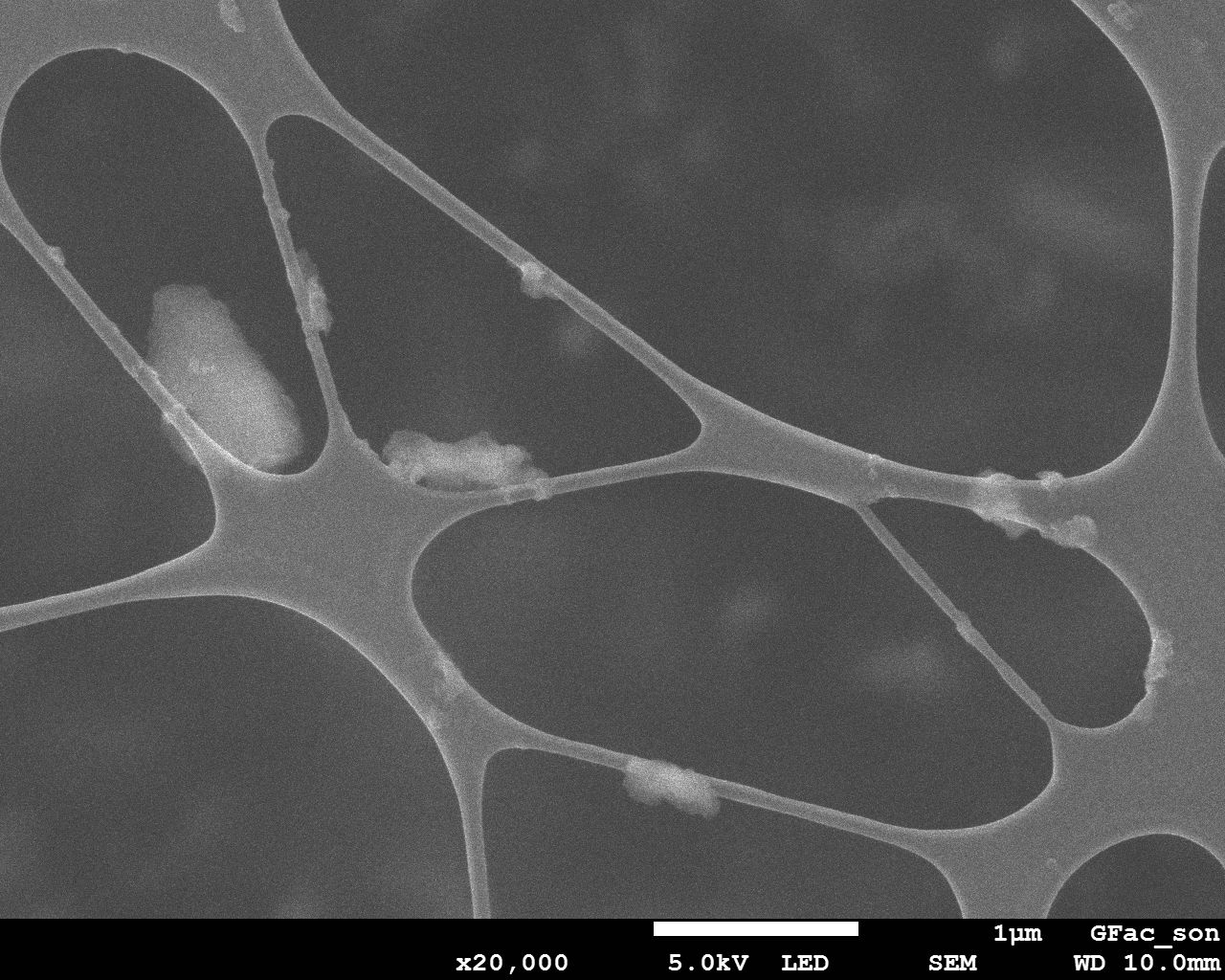

Supplement: Supplementary file 1 — ja3c13296_si_001.zip [file ja3c13296_si_001.zip › Data_archive/SEM/SEM small FG size/GF_ac_son_im023.jpg]

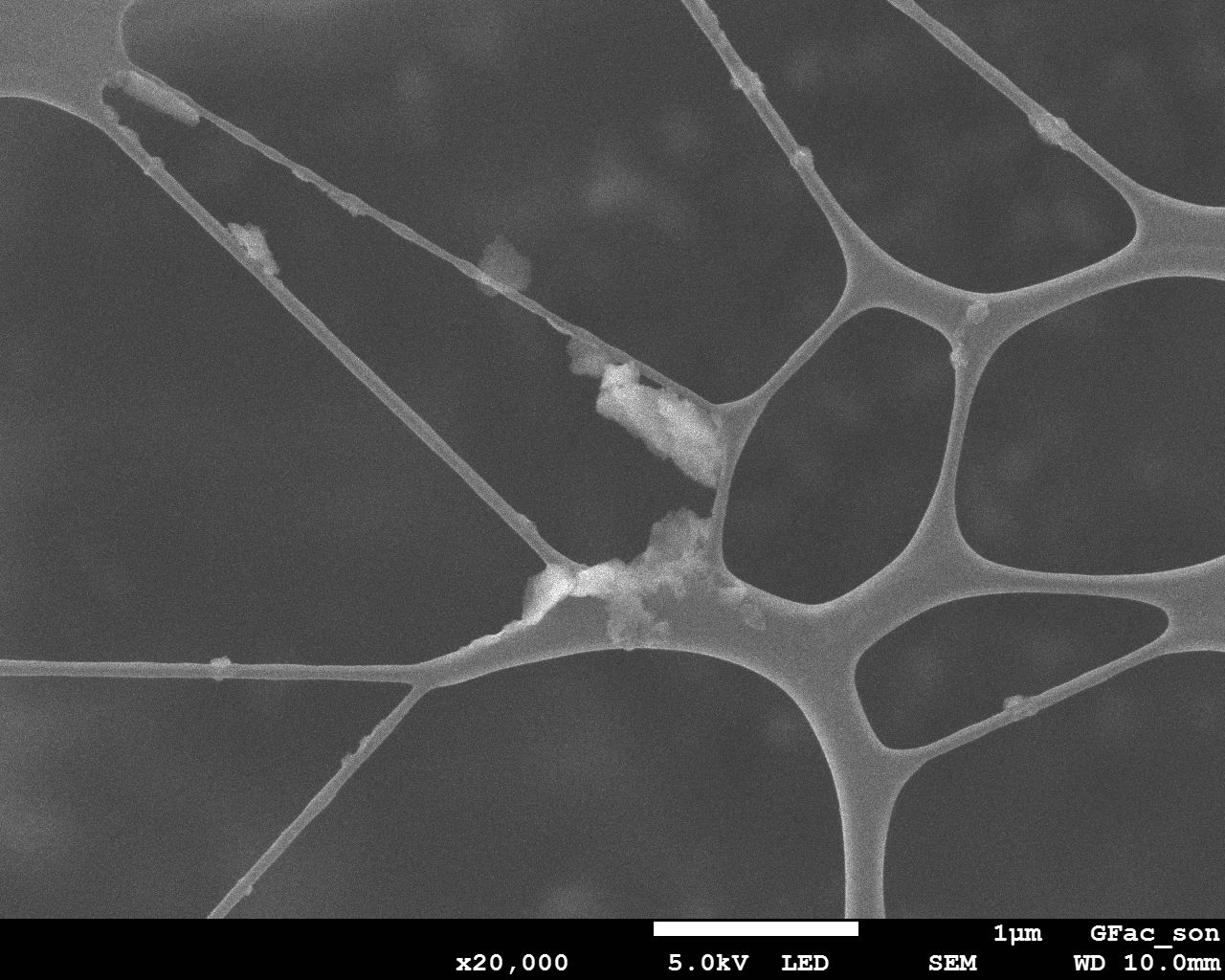

Supplement: Supplementary file 1 — ja3c13296_si_001.zip [file ja3c13296_si_001.zip › Data_archive/SEM/SEM small FG size/GF_ac_son_im024.jpg]

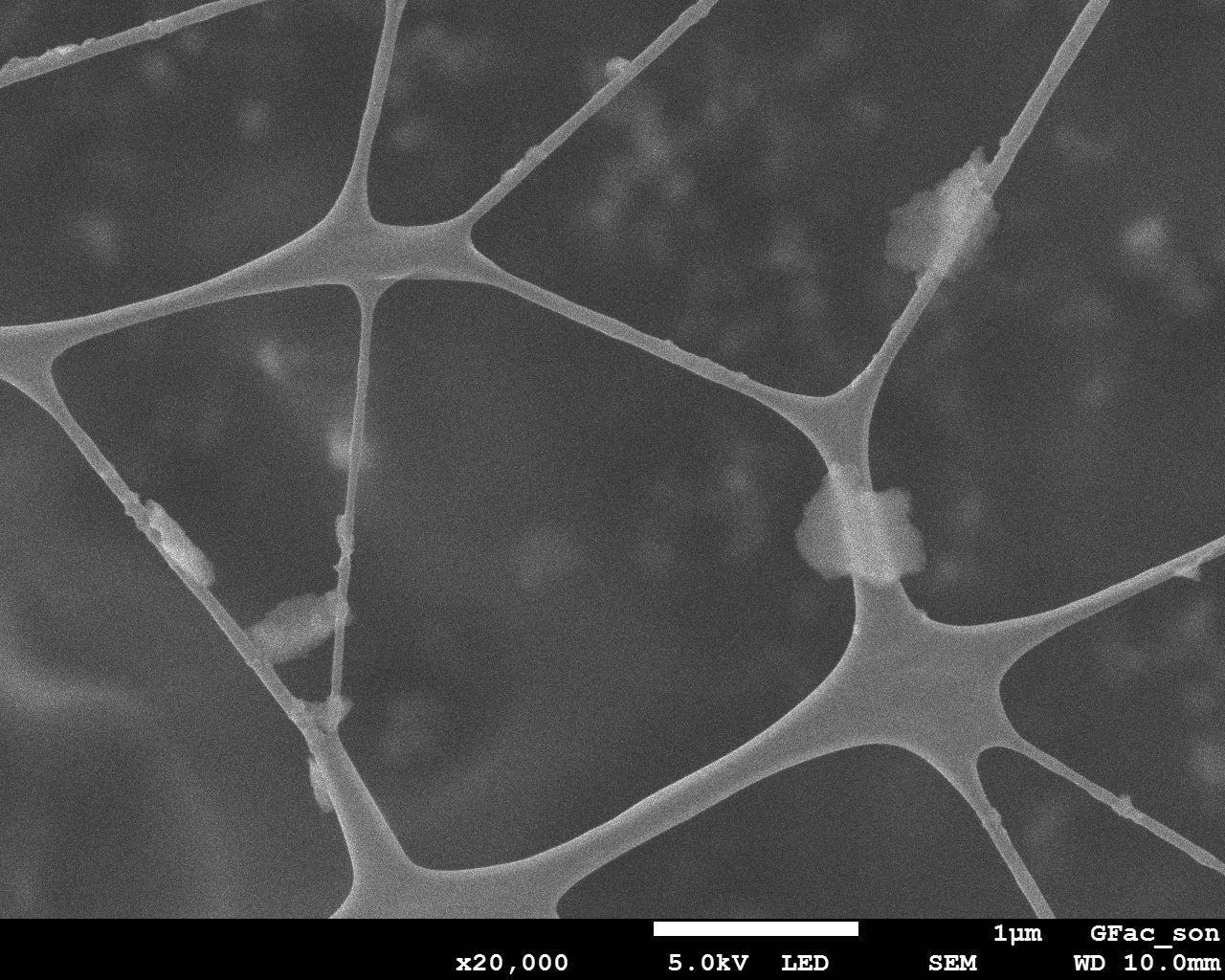

Supplement: Supplementary file 1 — ja3c13296_si_001.zip [file ja3c13296_si_001.zip › Data_archive/SEM/SEM small FG size/GF_ac_son_im025.jpg]

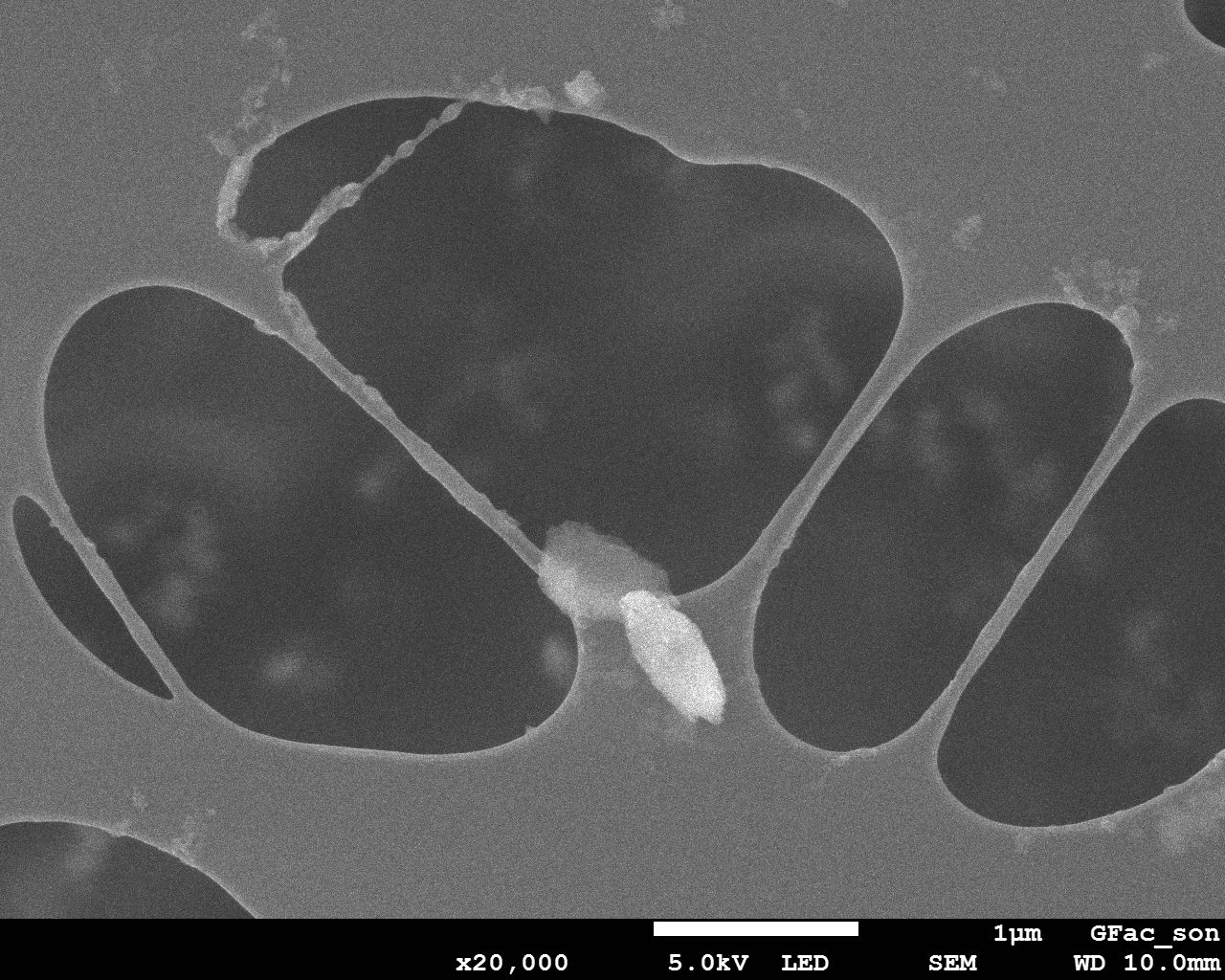

Supplement: Supplementary file 1 — ja3c13296_si_001.zip [file ja3c13296_si_001.zip › Data_archive/SEM/SEM small FG size/GF_ac_son_im026.jpg]

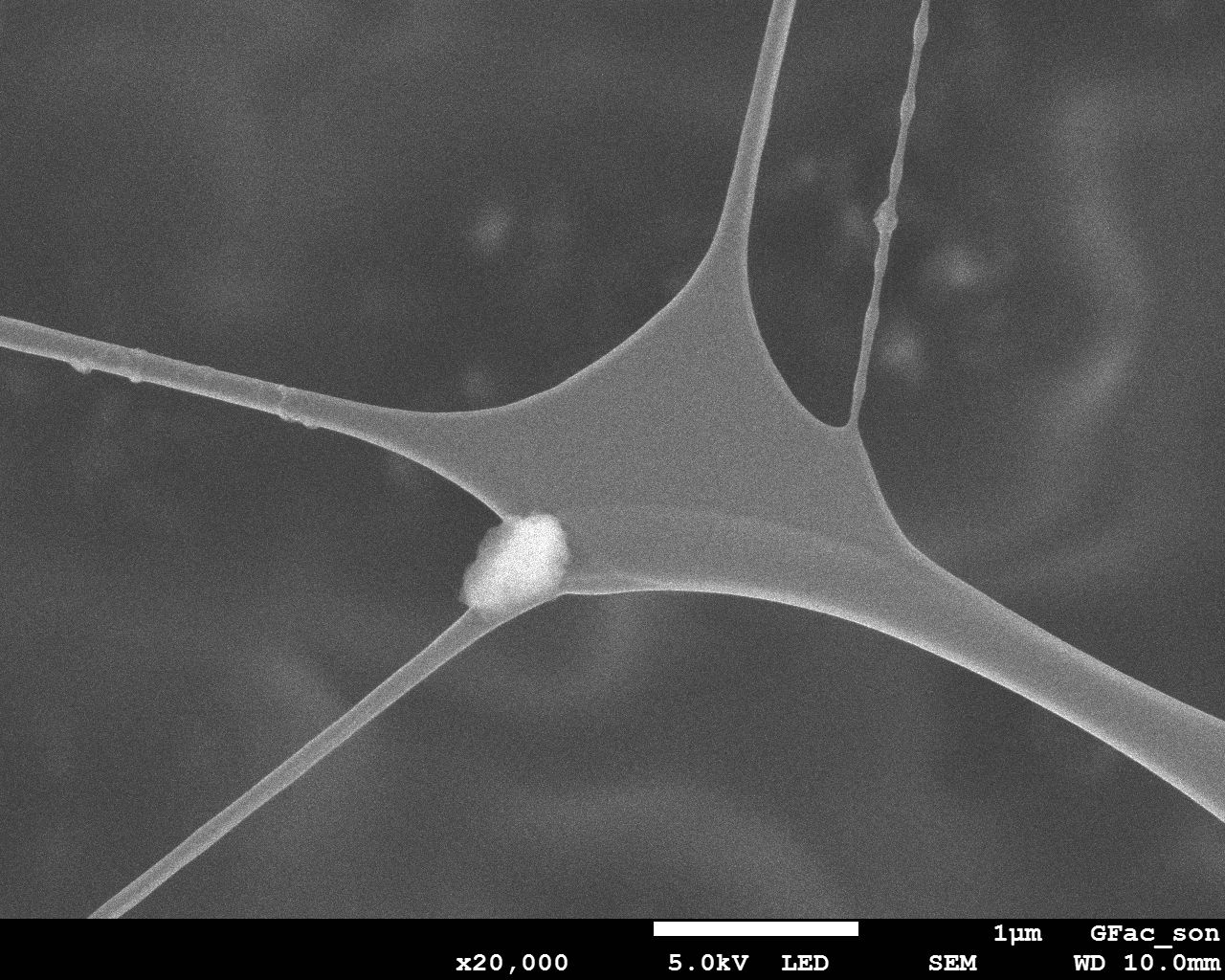

Supplement: Supplementary file 1 — ja3c13296_si_001.zip [file ja3c13296_si_001.zip › Data_archive/SEM/SEM small FG size/GF_ac_son_im027.jpg]

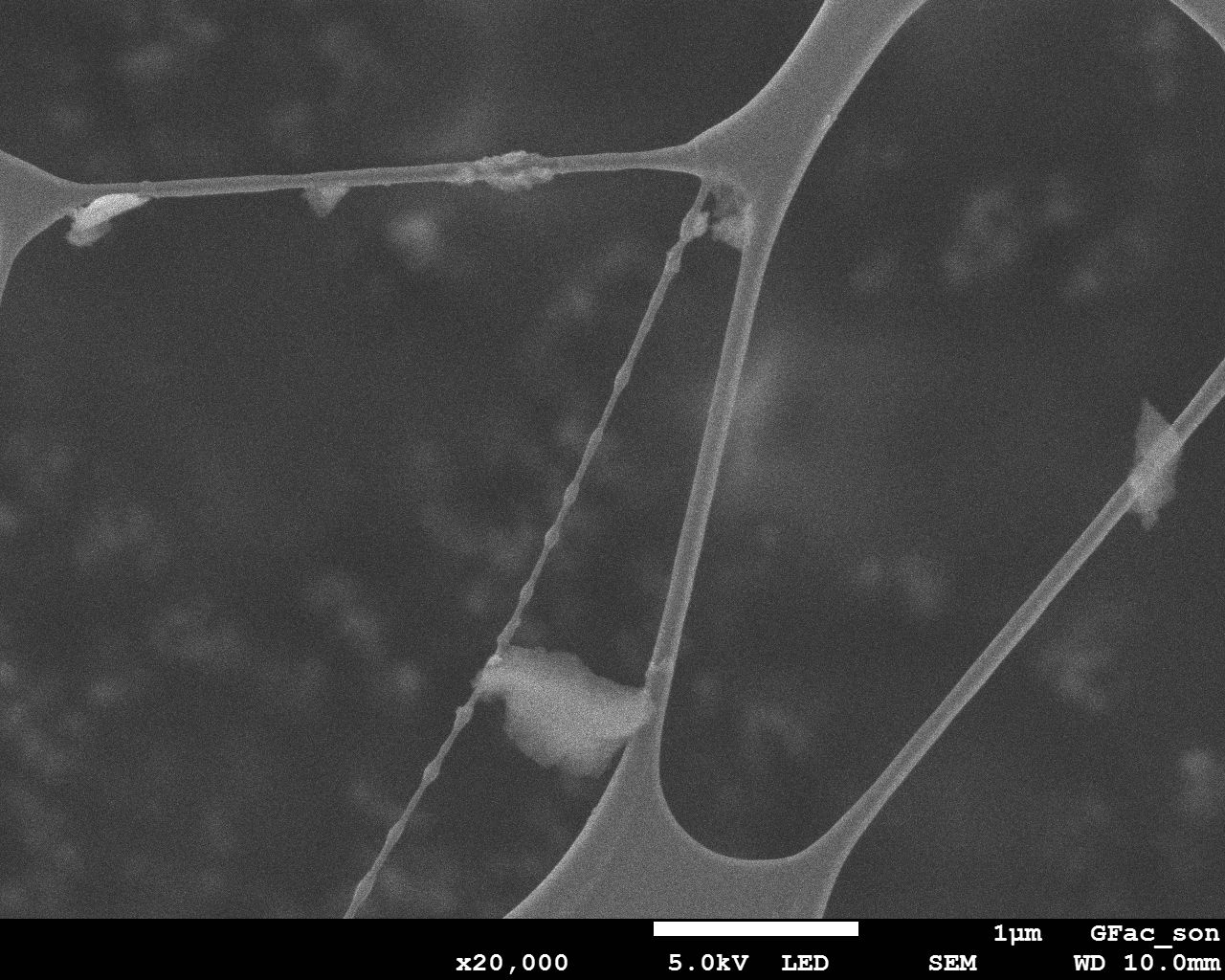

Supplement: Supplementary file 1 — ja3c13296_si_001.zip [file ja3c13296_si_001.zip › Data_archive/SEM/SEM small FG size/GF_ac_son_im028.jpg]

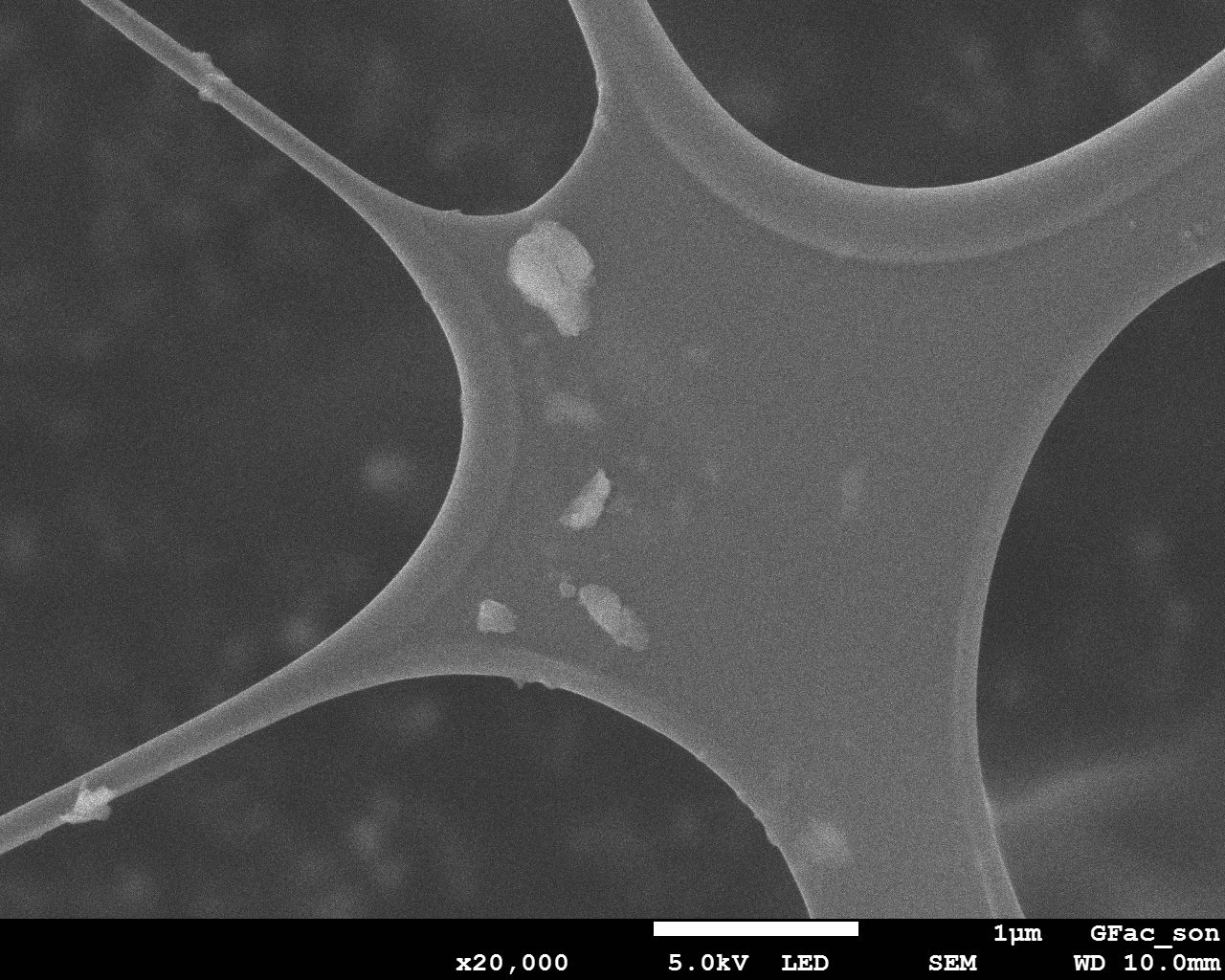

Supplement: Supplementary file 1 — ja3c13296_si_001.zip [file ja3c13296_si_001.zip › Data_archive/SEM/SEM small FG size/GF_ac_son_im029.jpg]

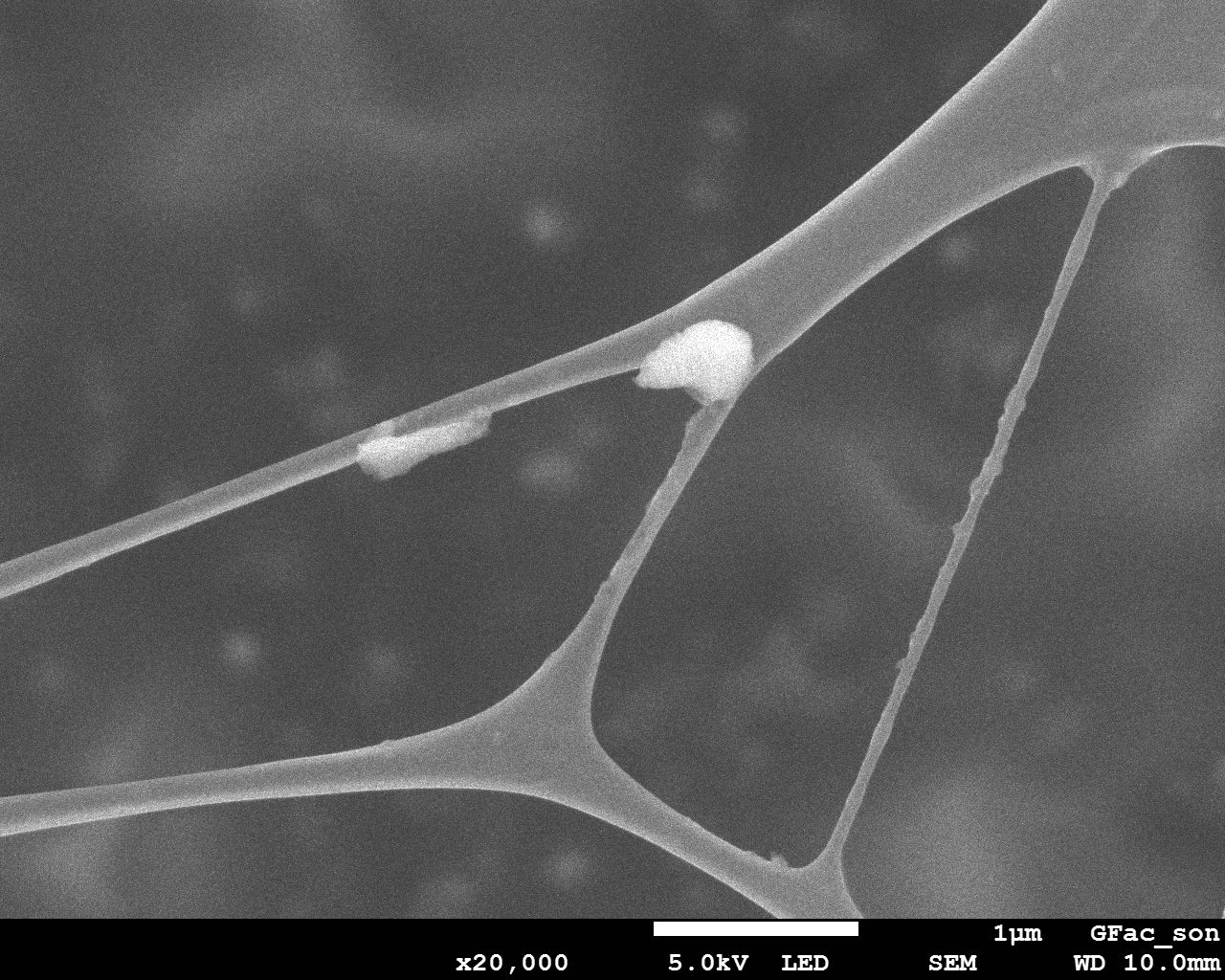

Supplement: Supplementary file 1 — ja3c13296_si_001.zip [file ja3c13296_si_001.zip › Data_archive/SEM/SEM small FG size/GF_ac_son_im030.jpg]

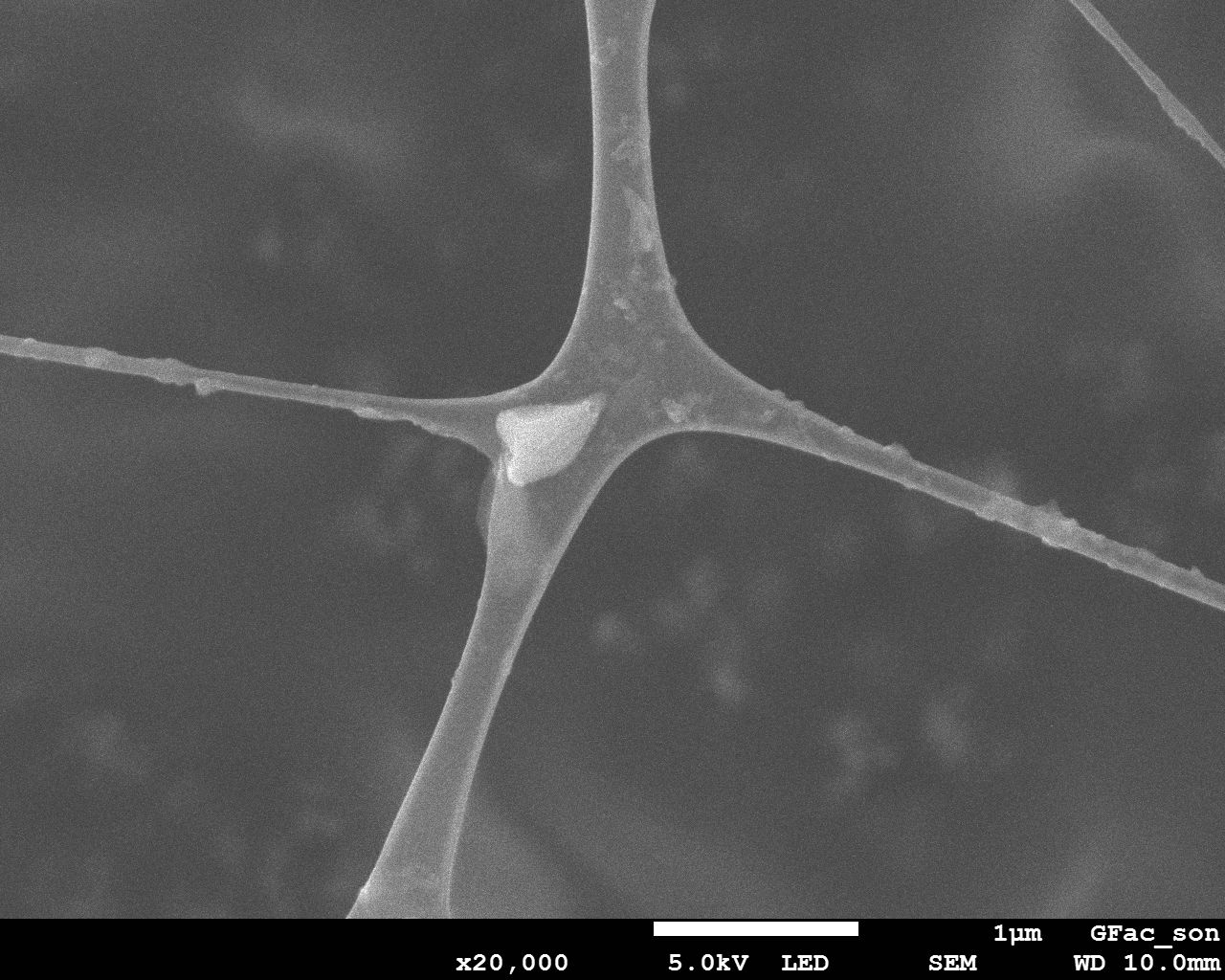

Supplement: Supplementary file 1 — ja3c13296_si_001.zip [file ja3c13296_si_001.zip › Data_archive/SEM/SEM small FG size/GF_ac_son_im031.jpg]

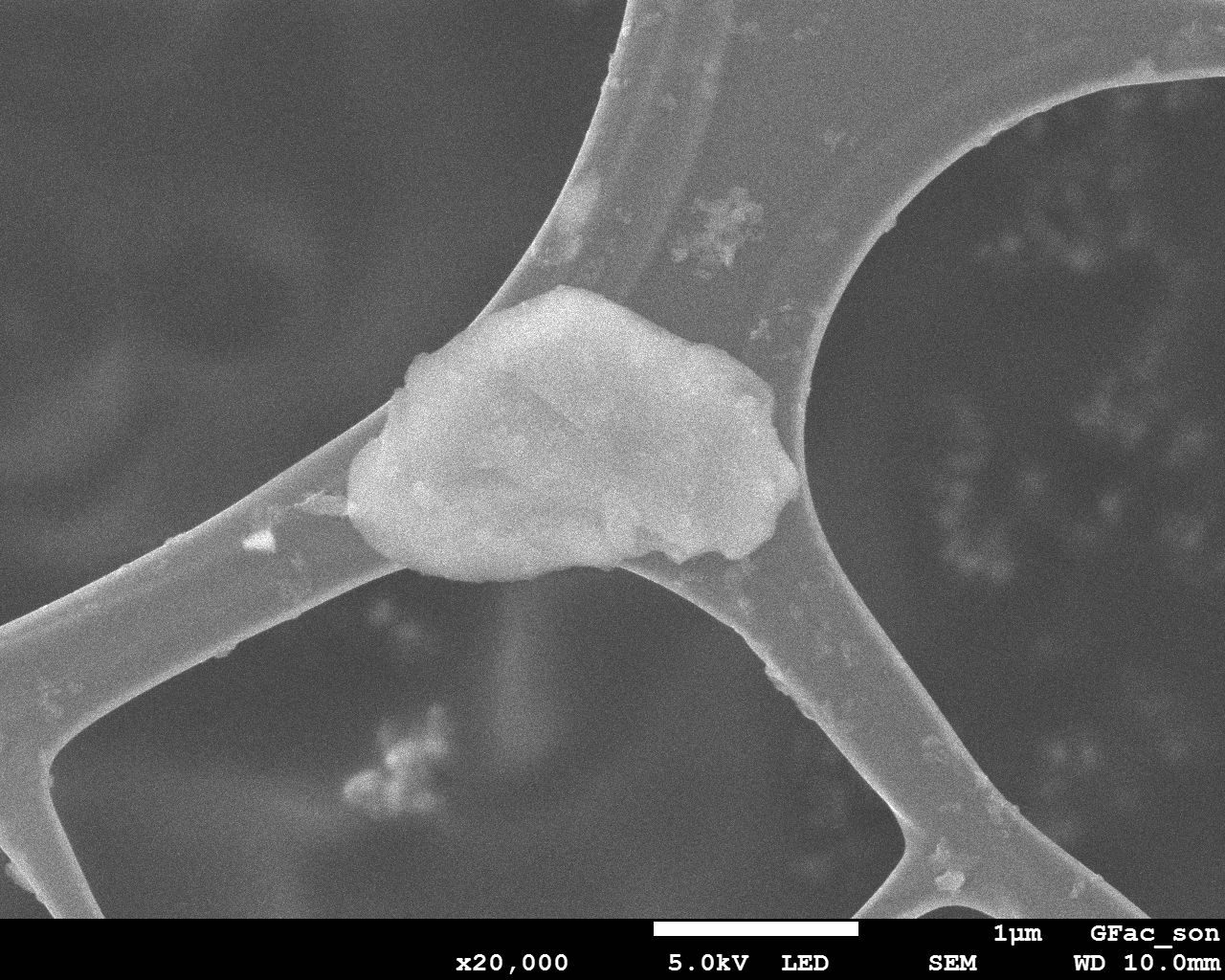

Supplement: Supplementary file 1 — ja3c13296_si_001.zip [file ja3c13296_si_001.zip › Data_archive/SEM/SEM small FG size/GF_ac_son_im032.jpg]

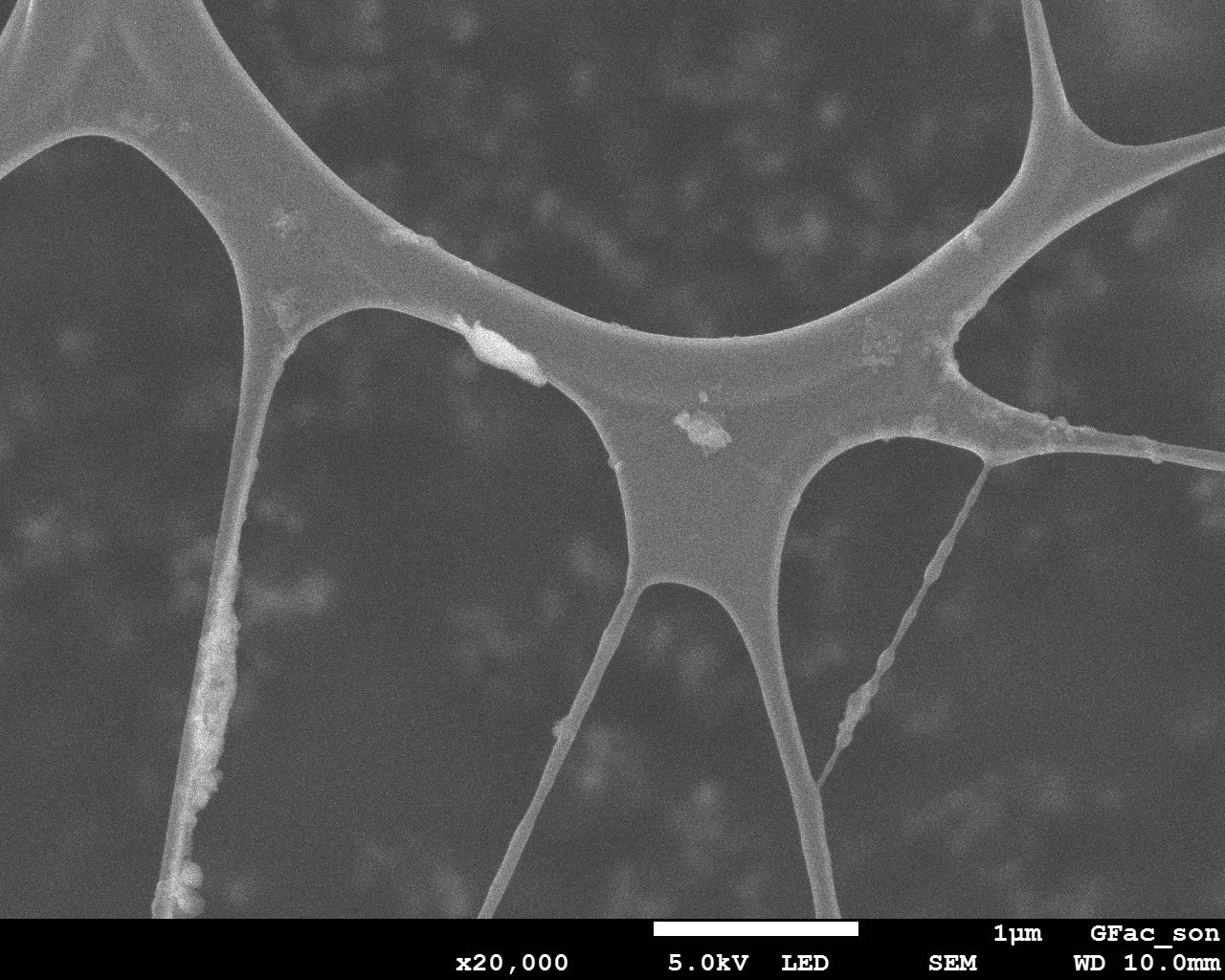

Supplement: Supplementary file 1 — ja3c13296_si_001.zip [file ja3c13296_si_001.zip › Data_archive/SEM/SEM small FG size/GF_ac_son_im033.jpg]

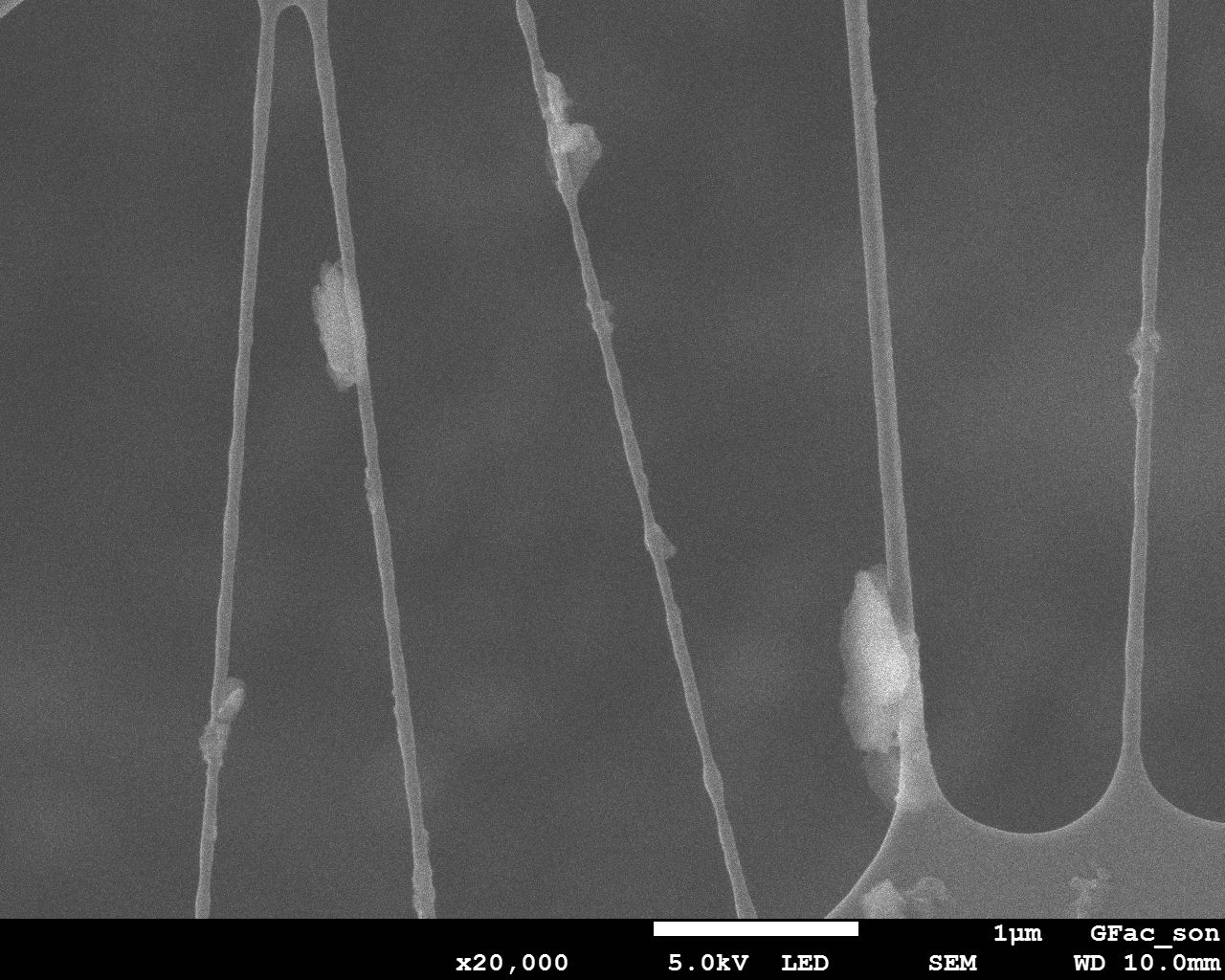

Supplement: Supplementary file 1 — ja3c13296_si_001.zip [file ja3c13296_si_001.zip › Data_archive/SEM/SEM small FG size/GF_ac_son_im034.jpg]

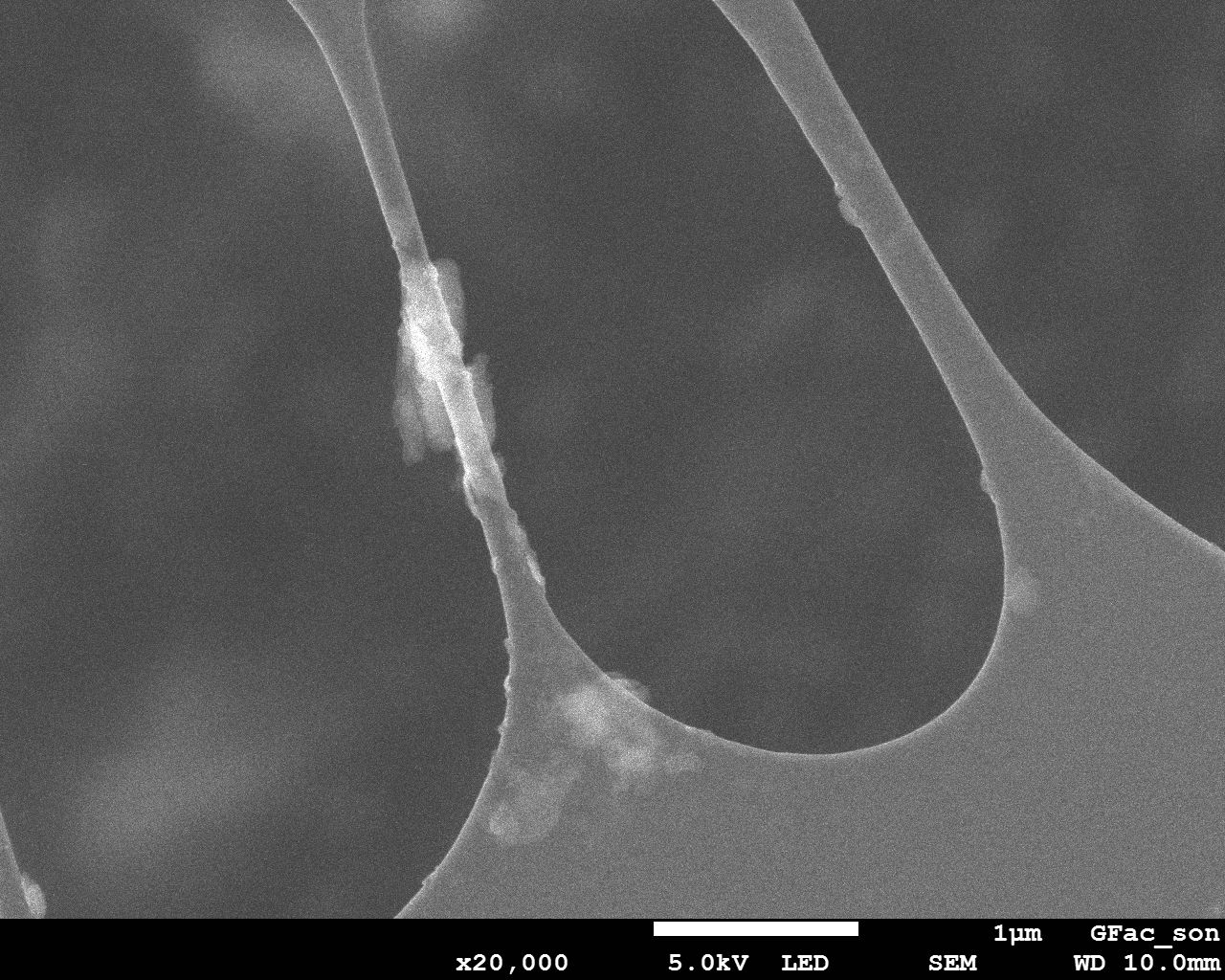

Supplement: Supplementary file 1 — ja3c13296_si_001.zip [file ja3c13296_si_001.zip › Data_archive/SEM/SEM small FG size/GF_ac_son_im035.jpg]

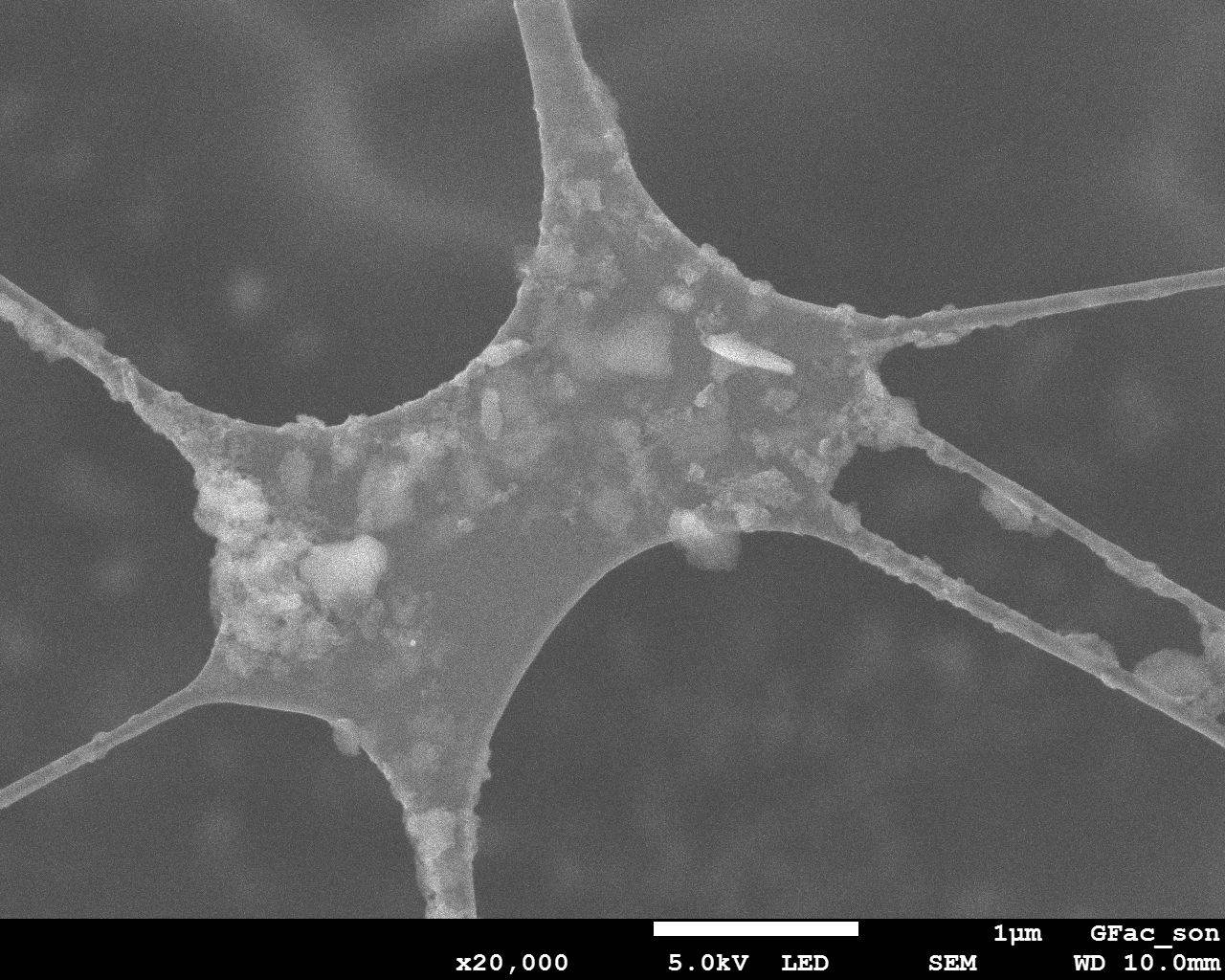

Supplement: Supplementary file 1 — ja3c13296_si_001.zip [file ja3c13296_si_001.zip › Data_archive/SEM/SEM small FG size/GF_ac_son_im036.jpg]

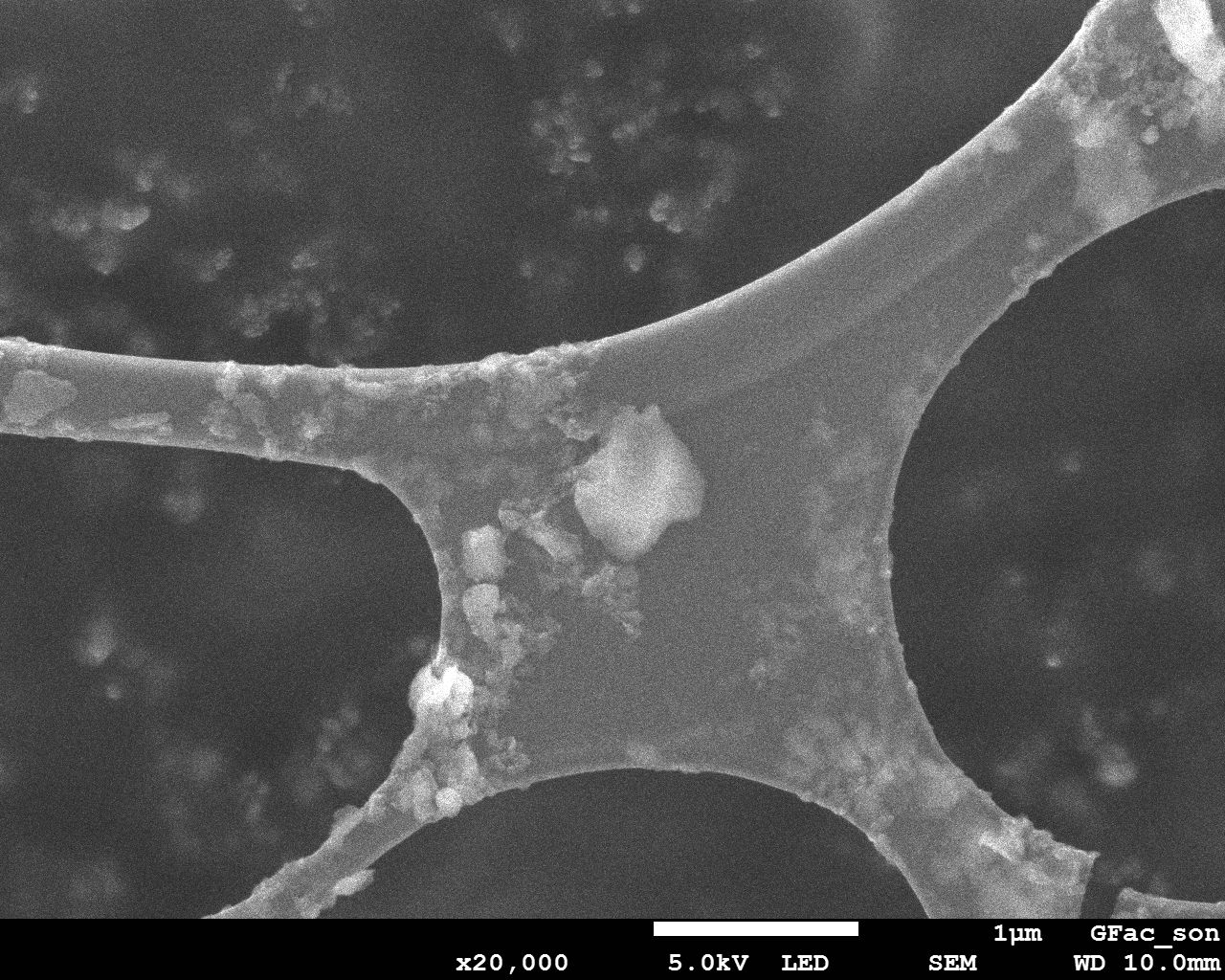

Supplement: Supplementary file 1 — ja3c13296_si_001.zip [file ja3c13296_si_001.zip › Data_archive/SEM/SEM small FG size/GF_ac_son_im038.jpg]

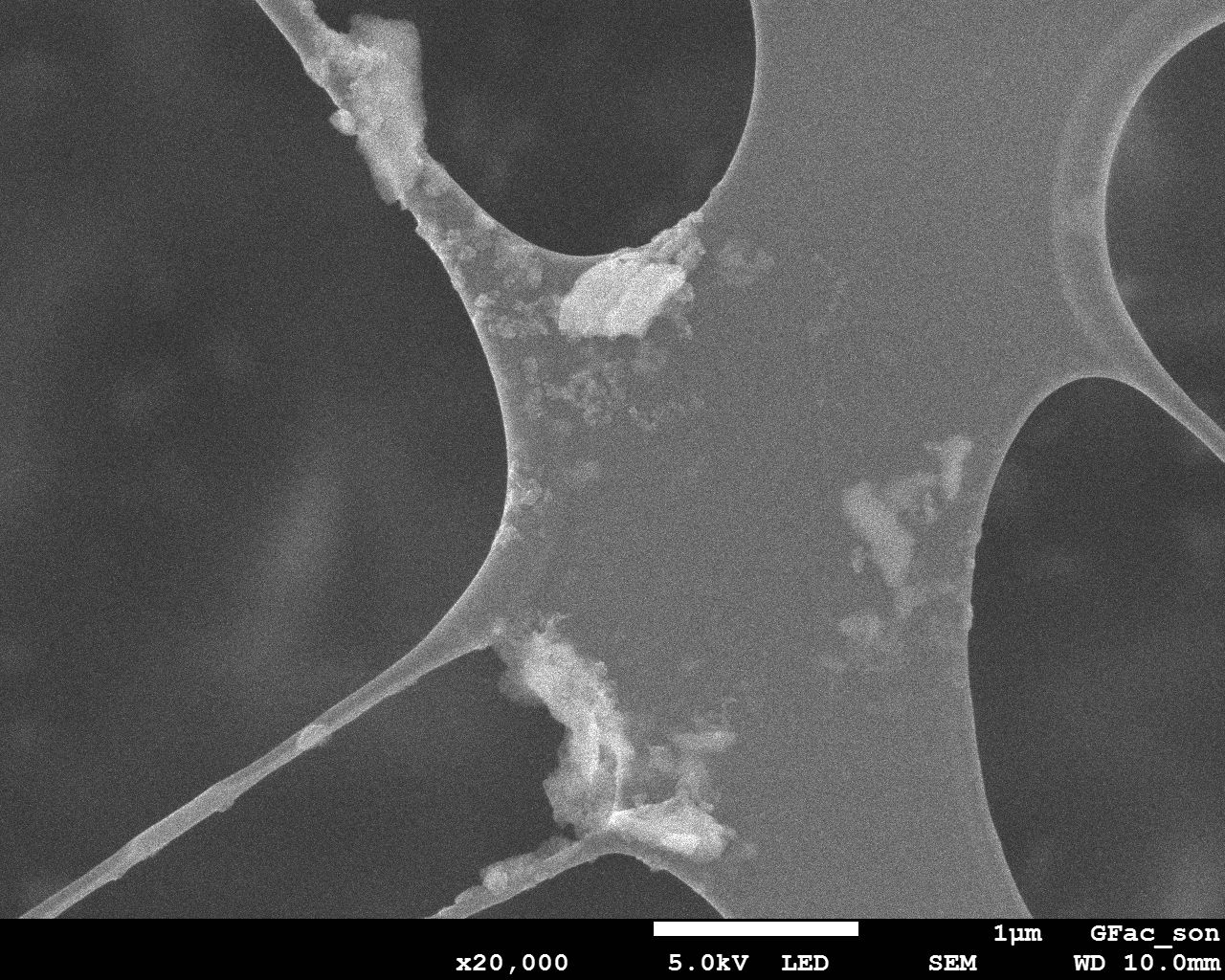

Supplement: Supplementary file 1 — ja3c13296_si_001.zip [file ja3c13296_si_001.zip › Data_archive/SEM/SEM small FG size/GF_ac_son_im039.jpg]

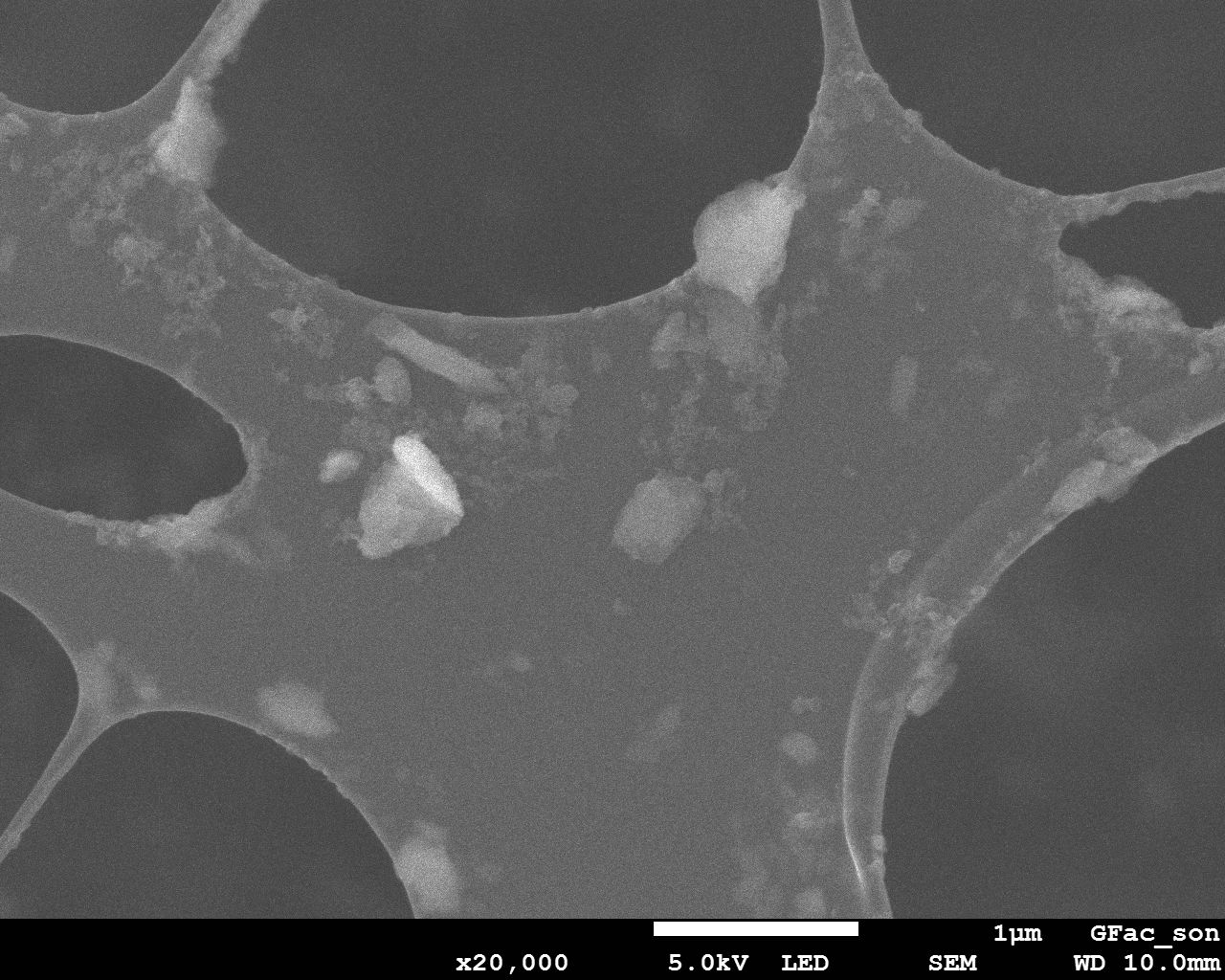

Supplement: Supplementary file 1 — ja3c13296_si_001.zip [file ja3c13296_si_001.zip › Data_archive/SEM/SEM small FG size/GF_ac_son_im040.jpg]
