# Supplementary material for: Solvent Controlled Generation of Spin Active Polarons in Two-Dimensional Material under UV Light Irradiation
Source: J Am Chem Soc. 2024 May 2;146(22):15010–8. doi: 10.1021/jacs.3c13296 (PMC11157526; doi:10.1021/jacs.3c13296)
Supplement: Supplementary file 2 — ja3c13296_si_002.pdf [file ja3c13296_si_002.pdf]

Supporting information

to

Solvent controlled generation of spin active polarons in 2D material under UV light irradiation

Giorgio Zoppellaro,[1,2] Miroslav Medveď,[1,3] Vítězslav Hrubý,[1,4] Radek Zbořil,[1,2] Michal Otyepka[1,5,*] and Petr Lazar[1,*]

1. Regional Centre of Advanced Technologies and Materials, The Czech Advanced Technology and Research Institute (CATRIN), Palacký University Olomouc, Šlechtitelů 27, 779 00 Olomouc, Czech Republic

2. Nanotechnology Centre, Centre for Energy and Environmental Technologies (CEET), VSB – Technical University of Ostrava, 17. listopadu 2172/15, 708 00 Ostrava-Poruba, Czech Republic

3. Department of Chemistry, Faculty of Natural Sciences, Matej Bel University, Tajovského 40, 974 01 Banská Bystrica, Slovak Republic

4. Department of Physical Chemistry, Faculty of Science, Palacký University Olomouc, 17. Listopadu 12, 771 46 Olomouc, Czech Republic

5. IT4Innovations, VSB – Technical University of Ostrava, 17. listopadu 2172/15, 708 00 Ostrava-Poruba, Czech Republic

The zip archive contains: raw data from EPR experiments, XRD, XPS, and FTIR characterization, SEM images for the size distribution determinations, *xyz* coordinates for DFT calculations using local models, structures and output files for periodic DFT models, BSE amplitudes and band character decompositions for periodic FG radical/acetone system.

Materials synthesis

For all the main experiments, a commercial FG (Graphite, fluorinated, polymer) from Sigma-Aldrich was used without any further treatment and was simply suspended in the solvent of interest. To inspect the effect of smaller FG sheets on the observed phenomena, 100 mg of FG from Sigma-Aldrich was dispersed in 50 mL of acetone (WVR Chemicals) in 100mL round-bottom flask, and sonicated for 4 hours using MS73 sonication horn (Bandelin Sonoplus) at power of 95 W with 0.3s pulses. To avoid heating of the sample and excessive evaporation of acetone, the flask with the dispersion was cooled by a water bath with a temperature of 2 °C controlled by a thermostat during the whole treatment. The total energy received by sonication was 293.708 kJ. After that, the bigger particles of the dispersion were allowed to sediment at room temperature for 20 hours. The upper part of the dispersion containing only the small FG flakes was then carefully taken using a pipette into a separate container and used for analyses and further experiments.

Characterization of the FG samples

The powder X-ray diffraction (XRD) pattern of the pristine FG was recorded on the Aeris diffractometer (Malvern PANalytical, Ltd, USA) in the Bragg–Brentano geometry (iron-filtered Co Kα radiation: λ = 0.178901 nm, 40 kV and 15 mA). Prior to the measurement, the FG was placed on a zero-background Si slide, gently pressed with sheet glass to create a uniform surface layer, and scanned with a step size of

0.0217° within a scan range from 5° to 105°. The recorded XRD pattern (Figure S1a) was indexed according to the reference.[1] Although looking amorphous-like, the position of the (002) diffraction line indicates the interlayer spacing between the individual fluorographene layers. The broadness of the line (FWHM of 3.72°) is caused by the irregularities in the stacking of the individual layers and many in-plane defects. The *d*-spacing was calculated according to Bragg's law

$$d = \frac{n\lambda}{2\sin\theta}$$

where d is the interplanar distance, n is an order of the diffraction ($n = 1$), λ is the wavelength of incident X-ray radiation in nm and θ is the diffraction angle (half of the 2θ position of the maximum reflection of the relevant plane). The overall XRD pattern and the determined interlayer spacing of 6.85 Å are typical of fully fluorinated graphite prepared by high-temperature fluorination of petroleum cokes.[1] Moreover, no (002) graphite diffraction line was observed.

The Fourier-transformed infrared (FTIR) spectra of both pristine and small FG sheets were recorded using a Nicolet iS5 FTIR spectrometer (Thermo Scientific) using the Smart Orbit ZnSe ATR accessory with nitrogen flow through the accessory. Before each measurement, the crystal was cleaned with ethanol after which the background was scanned. The pristine FG powder was pressed against the face of the ATR crystal. The acetone-based small FG dispersion was deposited onto the crystal and allowed to dry. The spectra were acquired with 4 cm^{-1} resolution by summing 32 scans in the range of 500-4000 cm^{-1}. The FTIR spectra of both pristine and small FG sheets were almost identical with only subtle differences (Figure S1b). The strongest band at 1210 cm^{-1} is typical of C-F stretching vibration while the shoulder at 1315 cm^{-1} was assigned to a vibration of CF_2 groups in the defects or edges of the FG sheets.

X-ray photoelectron spectroscopy (XPS) was performed using a PHI VersaProbe II (Physical Electronics) spectrometer using an Al Kα source (15 kV, 50 W). Both FG samples were deposited onto the silicon slide from acetone-based dispersions that were let to dry prior to the measurement. The obtained spectra were evaluated using the MultiPak (Ulvac - PHI, Inc.) software package. The spectral analysis included elemental composition determination with Shirley background subtraction. According to the analysis, the spectra of both pristine FG (Figure S1c) and small FG sheets (Figure S1d) were again almost identical with a minor difference in C/F ratio (see the insets of both panels). The minor defluorination of the small FG sheets was probably induced by the excessive sonication of the sample.

Finally, to determine the lateral sizes of FG structures, both samples were inspected using a scanning electron microscope (SEM) JEOL 7900F (JEOL, Japan), with an accelerating voltage of 5 kV. Both samples were drop-casted onto the carbon grid from the acetone-based dispersions and left to dry before the imaging. The size of an individual structure was recorded as the manually-measured longest margin of the structure on the acquired images. The pristine FG is rather polydisperse material exhibiting a comb size distribution (Figure S1e) with the highest frequency (10 %) in the size range of 2-2.5 µm. 91 % of the overall 385 analyzed structures were in the size range of 0-10 µm. Representative images of the pristine FG are in the Figure S3. The images show large particles with and smaller fragments evident layered structure. The sizes of the small FG sheets exhibited a monomodal distribution with the highest frequency (29 %) for the size range of 100-200 nm (Figure S1f). 99 % of the overall 267 analyzed structures were in the size range of 0-1300 nm. Representative images of the small FG sheets are in the Figure S4.

References

(1) Watanabe, N.; Nakajima, T.; Touhara, H. *Graphite Fluorides*; Studies in inorganic chemistry; Elsevier: Amsterdam ; New York, 1988.

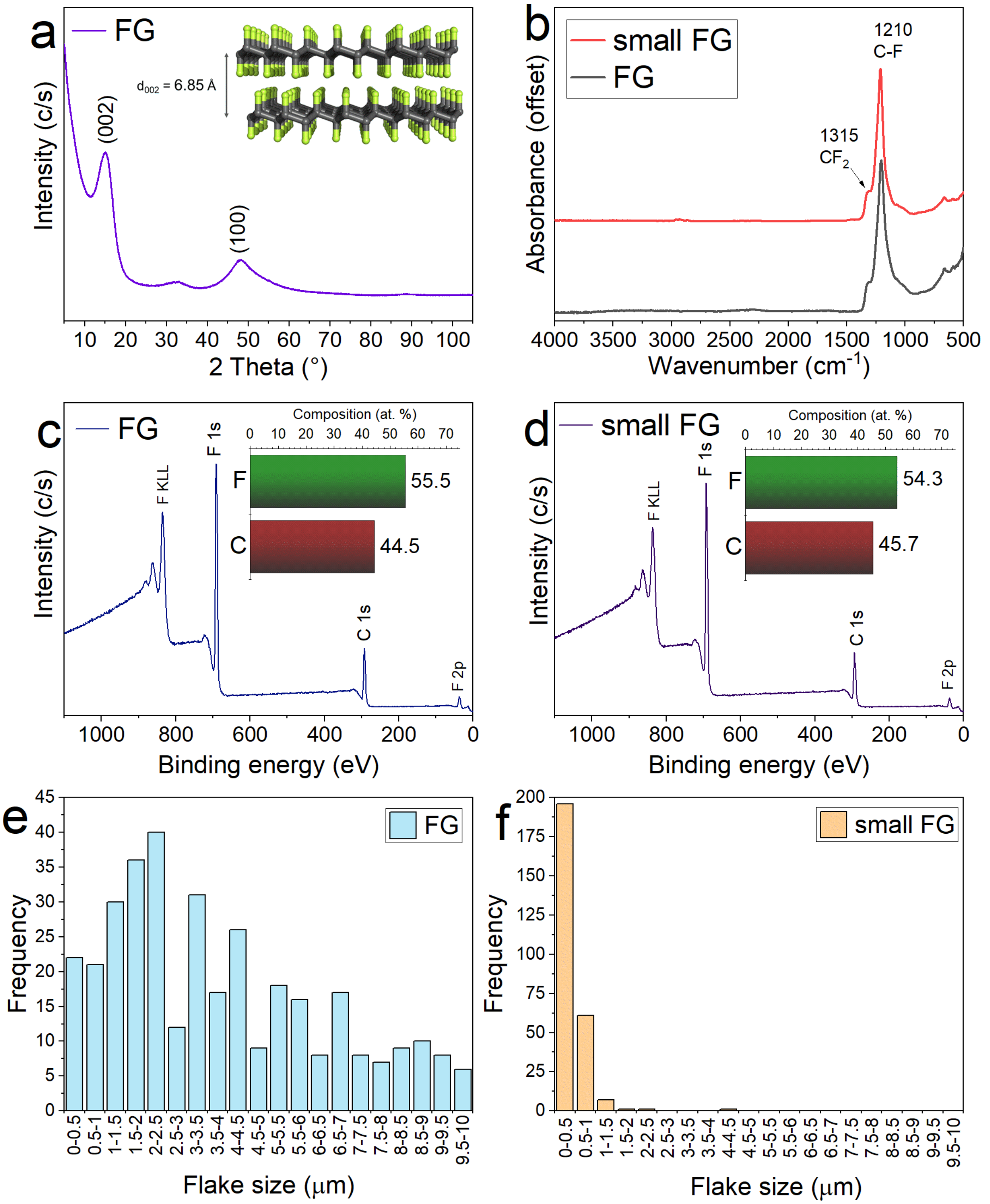

Figure S1. Characterization of the pristine FG and small FG prepared by horn sonication in acetone. XRD pattern of the pristine FG with the determined *d*-spacing between the individual FG sheets according to Bragg's law in the inset (a), comparison of FTIR spectra of pristine FG and small FG sheets with bands assigned to the individual vibration modes (b), survey XPS spectrum of pristine FG (c) and small FG sheets (d) with the determined elemental composition in the insets of both panels, and size distributions of the pristine FG (e) and small FG sheets (f) based on the SEM imaging.

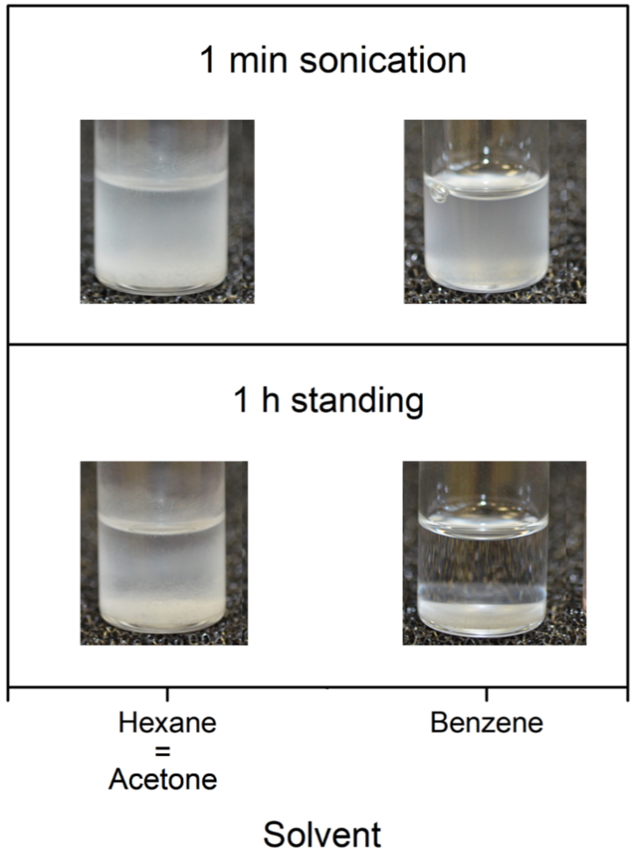

Figure S2. Optical photographs of commercial graphene fluoride powder (C_1F_1) (large sheets) dispersed in oxygen free hexane, benzene or acetone as solvents, recorded after 1 min of water-bath sonication (upper panel) and after 1h standing after the sonication, at room temperature (lower panel).

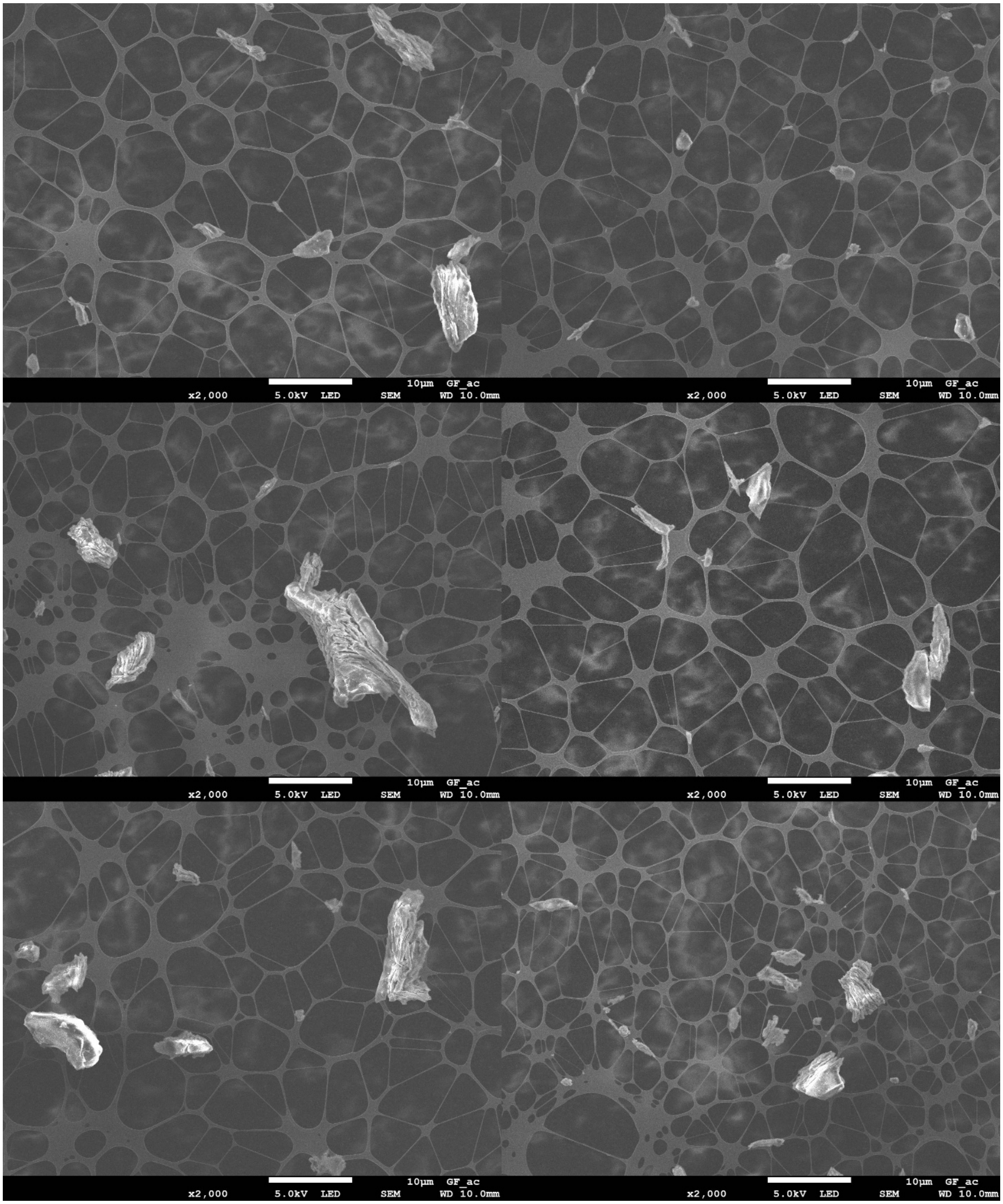

Figure S3. Representative SEM images of pristine FG.

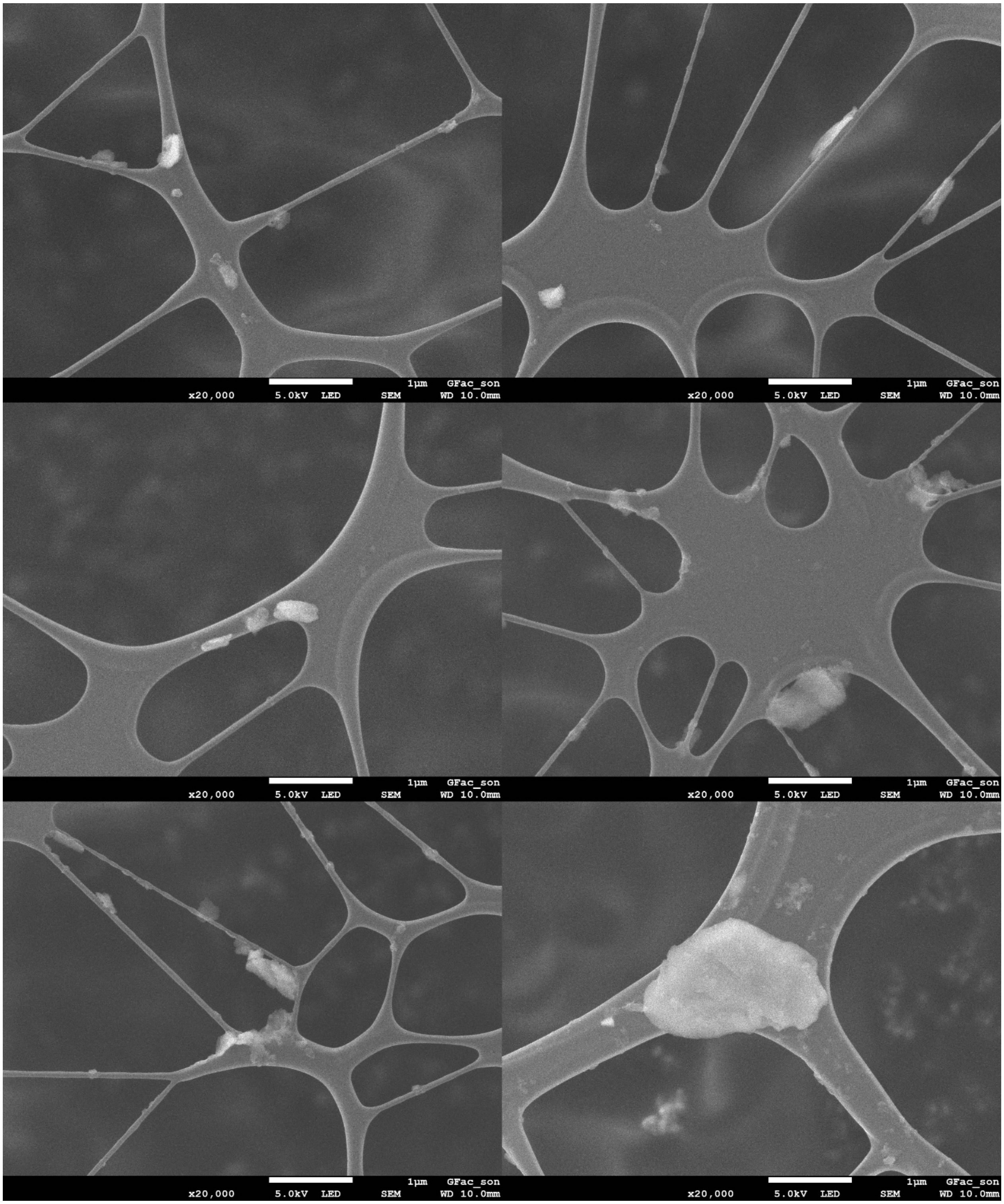

Figure S4. Representative SEM images of small FG sheets.

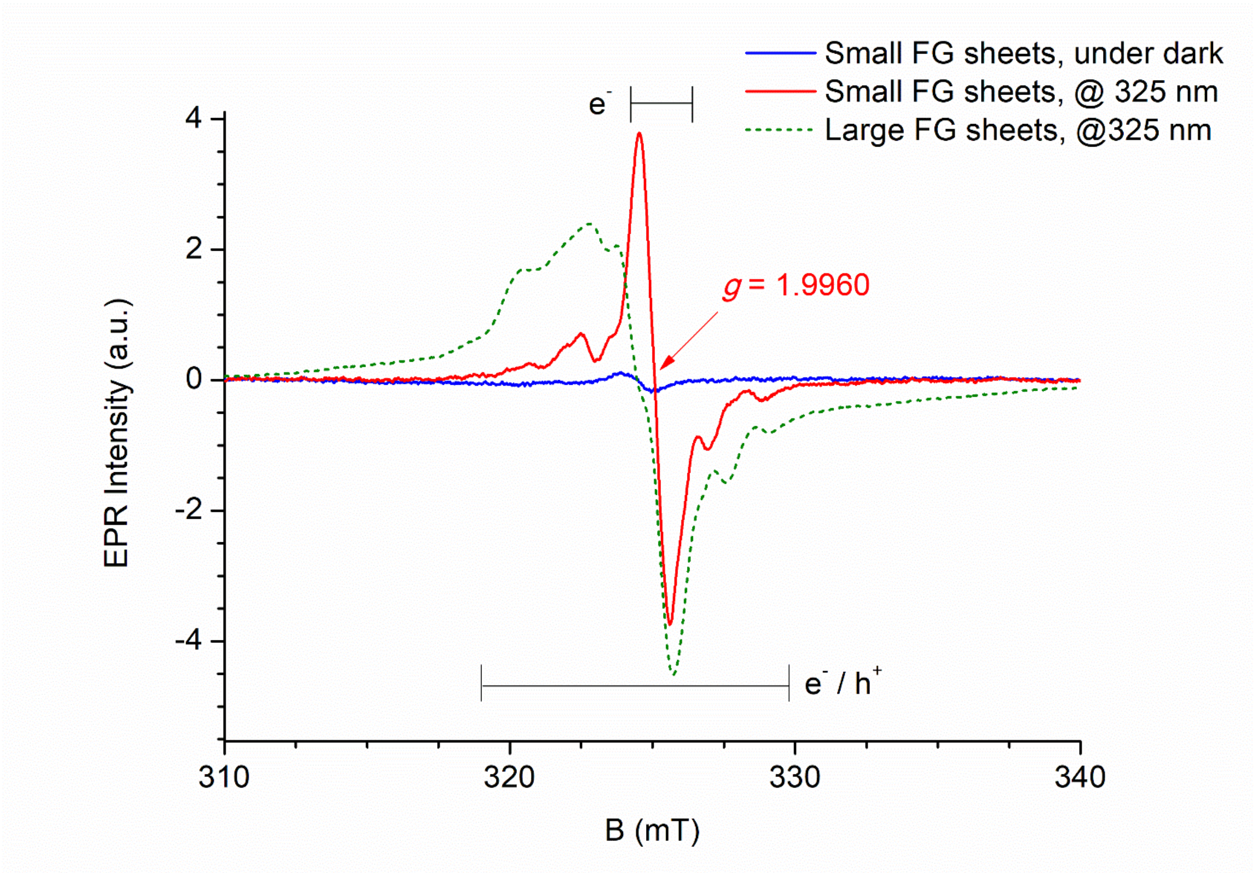

Figure S5. X-band EPR spectra of small-size FG sheets obtained by sonication (see materials synthesis), freshly dispersed in acetone solution, recorded under dark conditions (blue spectrum) and under *in situ* UV irradiation (@325 nm, red spectrum). The green spectrum (dotted line) corresponds to the large C_1F_1 sheets dispersed in acetone (commercial batch) recorded under UV irradiation; the resonance has been added here to allow easier comparison of the photoexcited spin species formed in small and large FG sheets. Experimental conditions used to record the resonance for the small FG sheets: 9.07649 GHz, 0.900 mW applied microwave power, 0.5 mT modulation width, T = 90 K. The irradiation time was set to 7.5 min before acquisition.

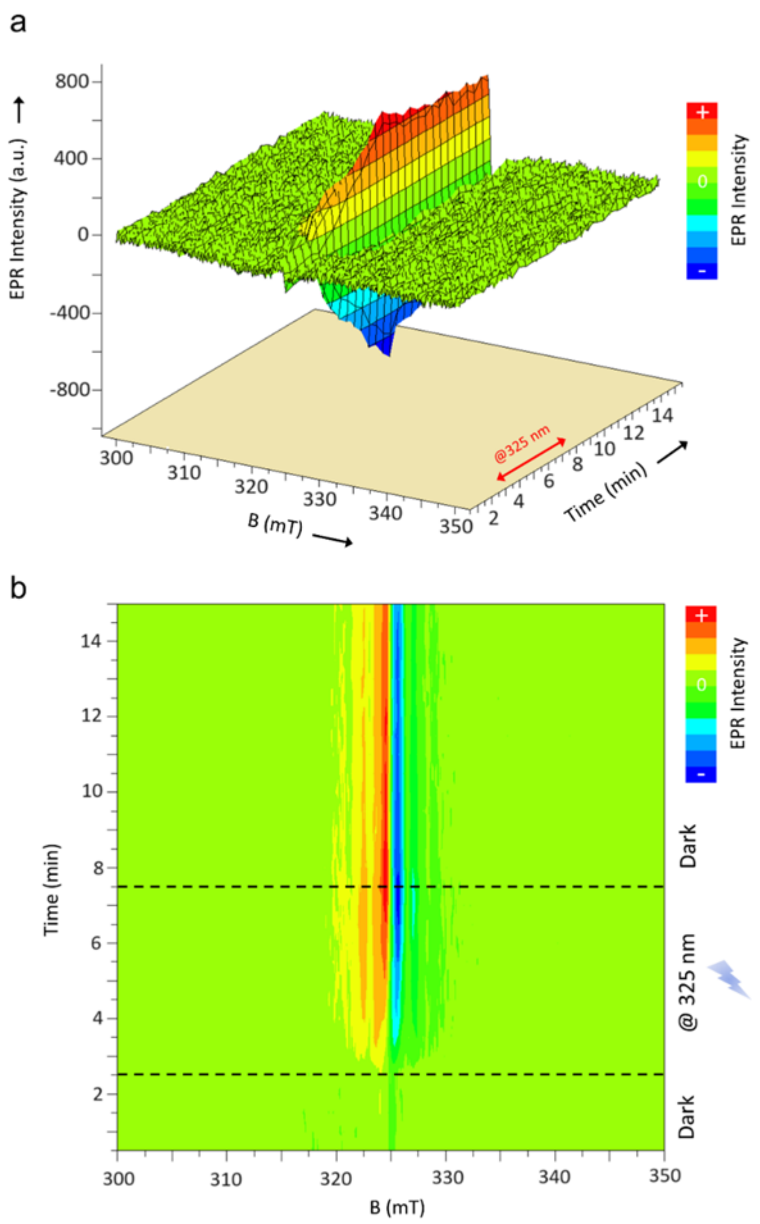

Figure S6. *In situ* light-induced X-band EPR spectra (LEPR) of the small sheets of FG obtained by sonication, being freshly dispersed in an oxygen-free acetone solution, showing the formation of spin active (S = ½) photoexcited states (e^-/h^+) upon irradiation. The sample was kept a in nitrogen-saturated atmosphere. Panel (a) shows the 3D LEPR plot (x, Field (B); y, time (min); z, EPR intensity). Experimental parameters during signal acquisition: 9.07682 GHz frequency, 0.6 mW microwave power, 100 kHz modulation frequency, 0.5 mT modulation width, 30 s acquisition time for each sequential spectrum, T = 90 K. Signal acquisition sequence: 2.5 min under dark conditions, followed by 5.0 min under light irradiation (@ 325 nm), then 7.5 min under dark conditions. Panel (b) shows the correspondent 2D LEPR plot of the spectra shown in panel (a).

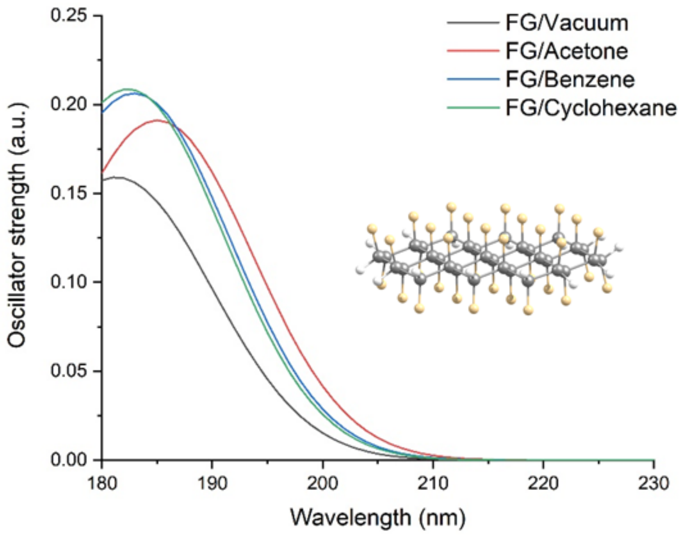

Figure S7. Absorption spectra of pristine FG (modelled as fluorinated ovalene shown in inset) in vacuum (black line), acetone (red line), benzene (blue line), and cyclohexane (green line) computed without an explicit solvent molecule, i.e., the solvent effects were only included implicitly using the SMD solvation model. Computational level: CAM-B3LYP/6-31++G(d,p). The inhomogeneous broadening was mimicked by using FWHM = 20 nm.

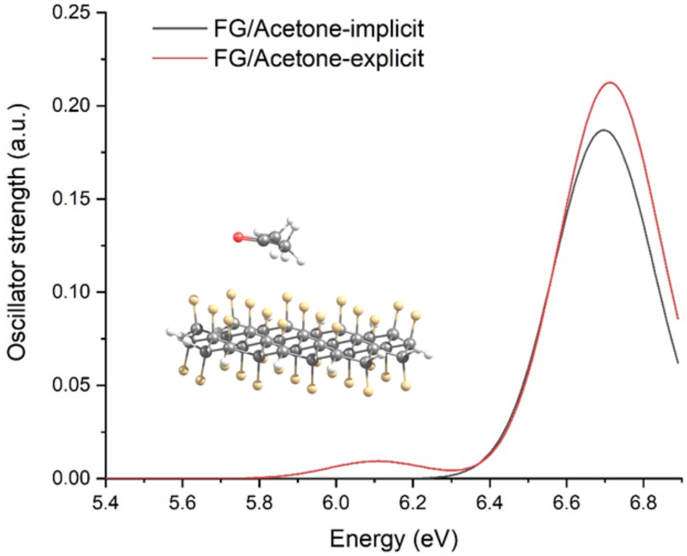

Figure S8. Absorption spectra of pristine FG (modelled as fluorinated ovalene) in acetone computed without (black line) and with (red line) an explicit solvent molecule (as shown in the inset). Computational level: CAM-B3LYP/6-31++G(d,p)/SMD(solvent = acetone). The inhomogeneneous broadening was mimicked by using FWHM parameter set to 0.3 eV.

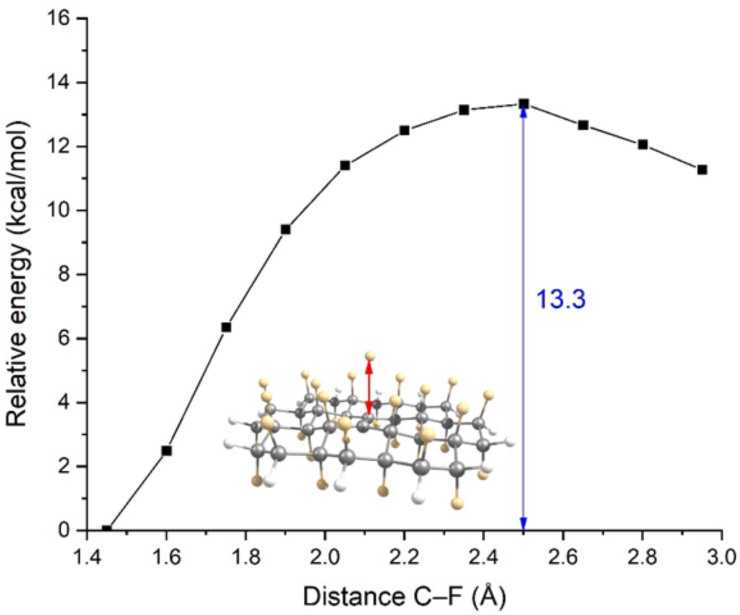

Figure S9. Relaxed scan along the C–F distance (marked by a red arrow in the inset) of anionic defective FG (modelled as fluorinated ovalene with a removed fluorine atom) in acetone. The reference level corresponds to the fully optimized structure. Computational level: ωB97X-D/6-31++G(d,p)/SMD(solvent = acetone).

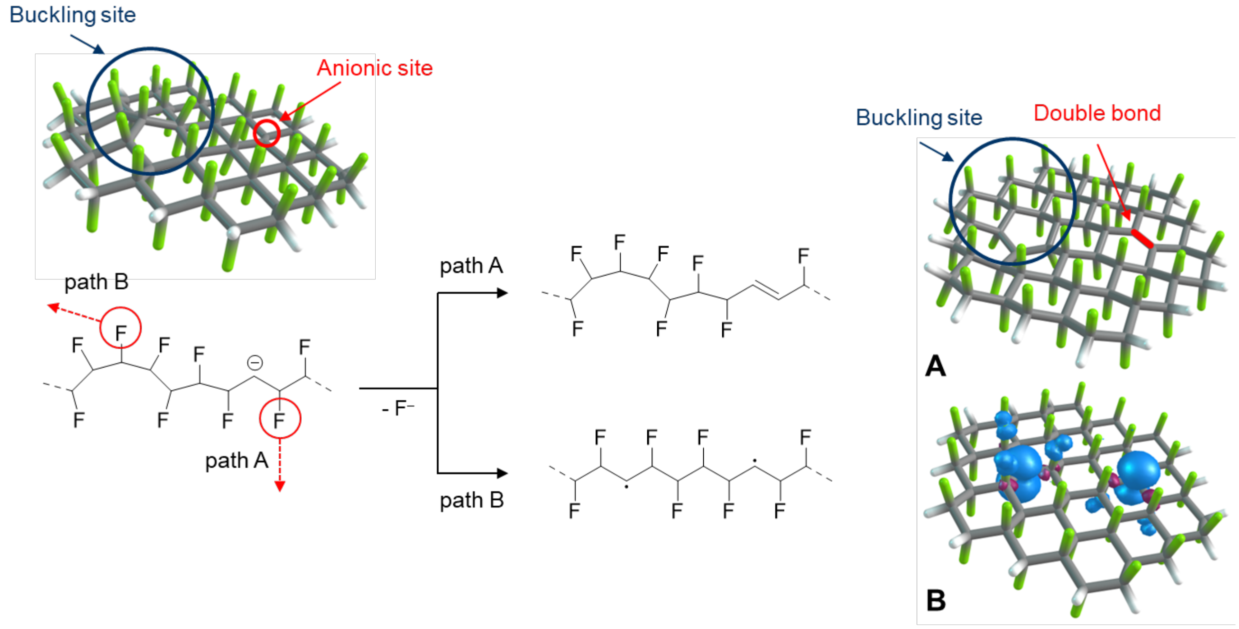

Figure S10. Possible defluorination pathways in FG model containing a buckle defect nearby an anionic site. Path A: Defluorination occurring next to the anionic site leading to formation of a double bond. Path B: Defluorination occurring at the buckling site leading to formation of spin active species (spin density surface is displayed for the isovalue = 0.0025).

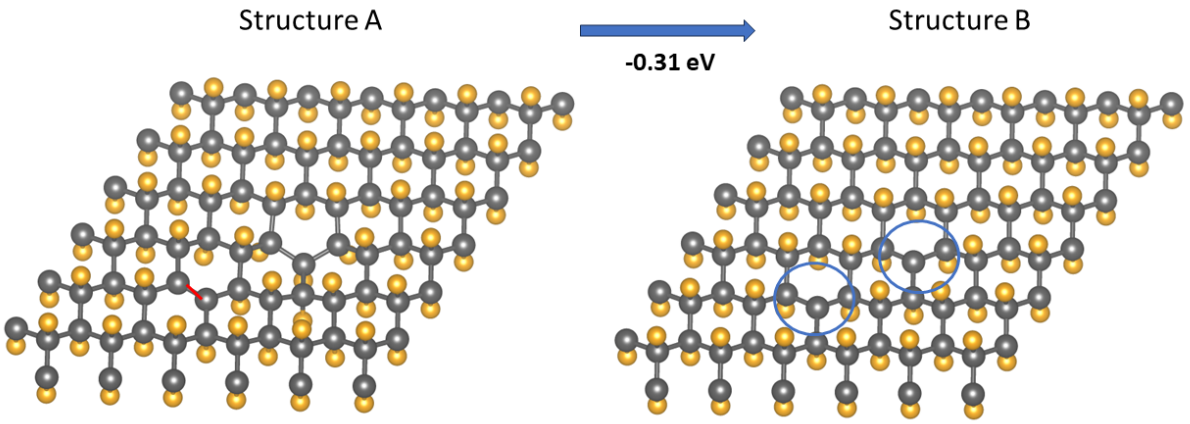

Figure S11. Possible defluorination pathways in periodic FG model containing a buckle defect nearby an anionic site. Structure A: Defluorination occurring next to the anionic site leading to formation of a double bond. Structure B: Defluorination occurring at the buckling site leading to formation of spin active species

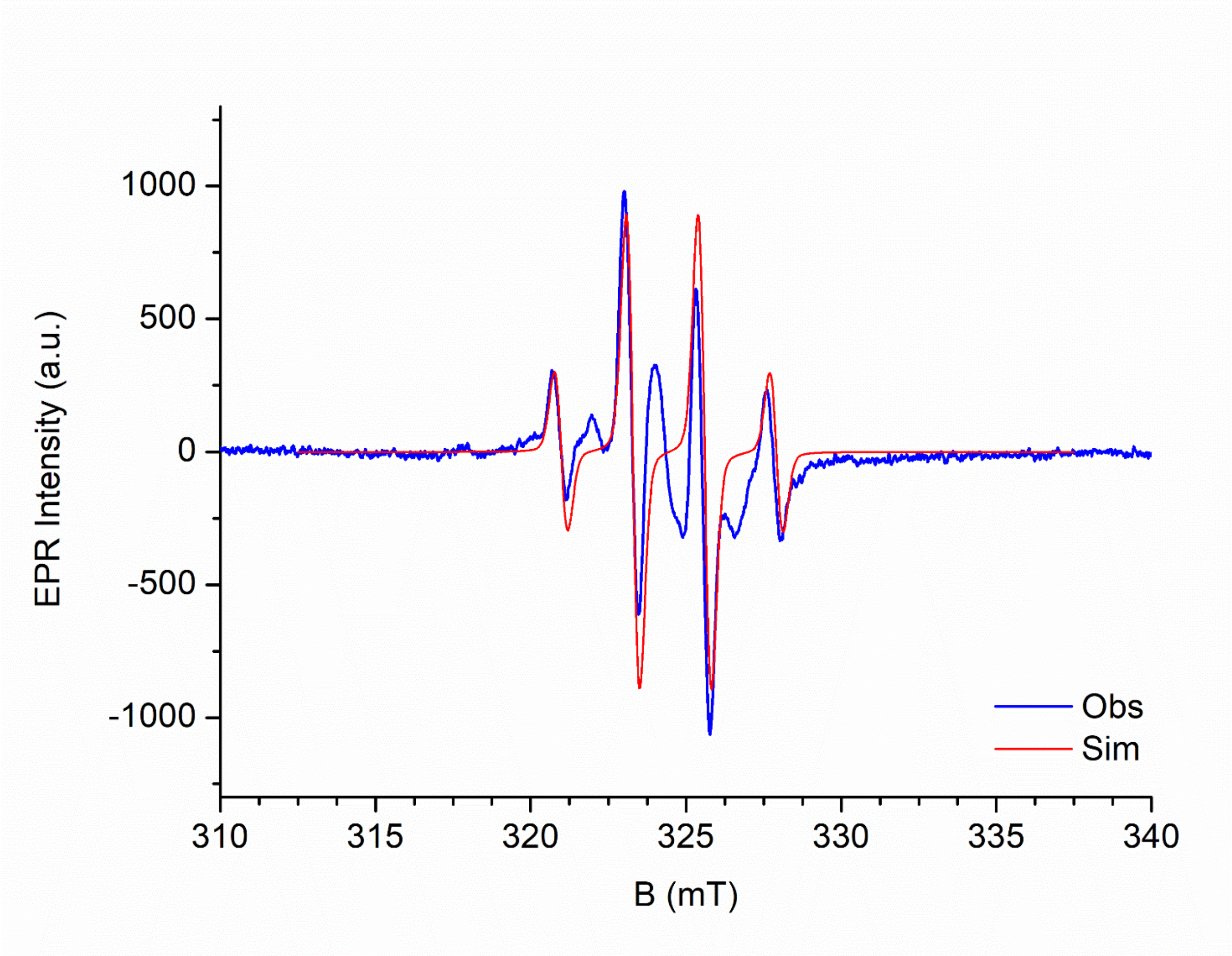

Figure S12. X-band EPR spectra of graphene fluoride powder (C_1F_1) (large sheets) freshly dispersed in oxygen free acetone solution recorded at T = 90 K after 3 min under in situ UV irradiation (@325 nm).

Experimental parameters: 9.07548 GHz, 100 kHz modulation frequency, 0.99800 mW power, 0.5 mT modulation width, 0.03 s time constant, 2 min sweep time. The dotted spectrum represents the computer simulation (S=1/2) by perturbation theory (second order) with spin-Hamiltonian parameters as follows: g_e-tensor (x,y,z) = 2.0013, 2.0013, 2.0013; A-tensor = 22.5 G, 22.5 G, 22.5 G (g_n-tensor: 2.000, 2.000, 2.000), number 3 ^{1}H (I=1/2). Line shape (x,y,z) = 5.0 G, 5.0 G, 5.0 G. Integration sphere (phi, theta) = 200, 200. Lorentzian/Gaussian ratio = 1.00. WinEPR SimFonia software (ver. 1.25, Bruker GmbH).

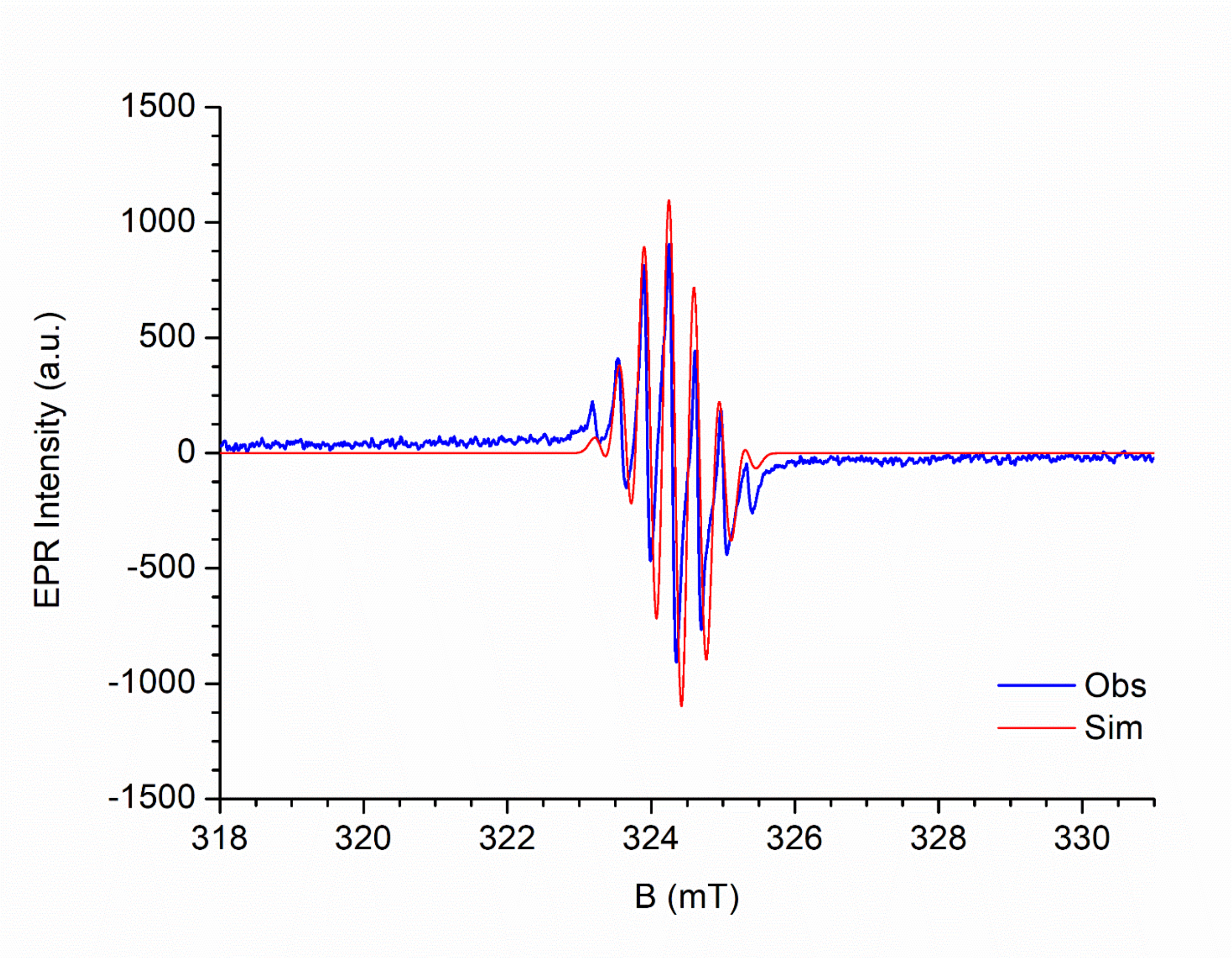

Figure S13. X-band EPR spectrum of freshly prepared C_1F_1/deuterated acetone (acetone-d_6) (large sheets) recorded at T = 90 K in oxygen-free solution and recorded after 7.5 min under UV irradiation (@325 nm) followed by 10 min under dark conditions. Experimental parameters: 9.07383 GHz, 100 kHz modulation frequency, 0.09970 mW power, 0.05 mT modulation width, 0.03 s time constant, 1 min sweep time, 9 scans accumulated and averaged. The dotted spectrum represents the computer simulation (S=1/2) by perturbation theory (second order) with spin-Hamiltonian parameters as follows: g_e-tensor (x,y,z) = 2.0005, 2.0005, 2.0005; A-tensor = 3.4 G, 3.4 G, 3.4 G (g_n-tensor: 2.000, 2.000, 2.000), number 6 ^{19}F (I=1/2). Line shape (x,y,z) = 2.0 G, 2.0 G, 2.0 G. Integration sphere (phi, theta) = 200, 200. Lorentzian/Gaussian ratio = 1.00. WinEPR SimFonia software (ver. 1.25, Bruker GmbH).

Note on the use of deuterated acetone d_6: In this way one can learn whether the hydrogens bonded to carbons interact with the electron spin of the free radical. The proton and deuteron have different spins, 1/2

and 1, respectively, also have different magnetic moments, 2.79255 and 0.857354, respectively. For these reasons the structures which they impart to the electron spin resonance patterns are substantially different. A single interacting proton gives a doublet, whereas a deuteron substituted would give a triplet with components of equal intensity and with only about one sixth the spacing of the doublet. In general, the total spread of the hydrogen hyperfine structure of a given free radical will be about three times that of its completely deuterated counterpart.

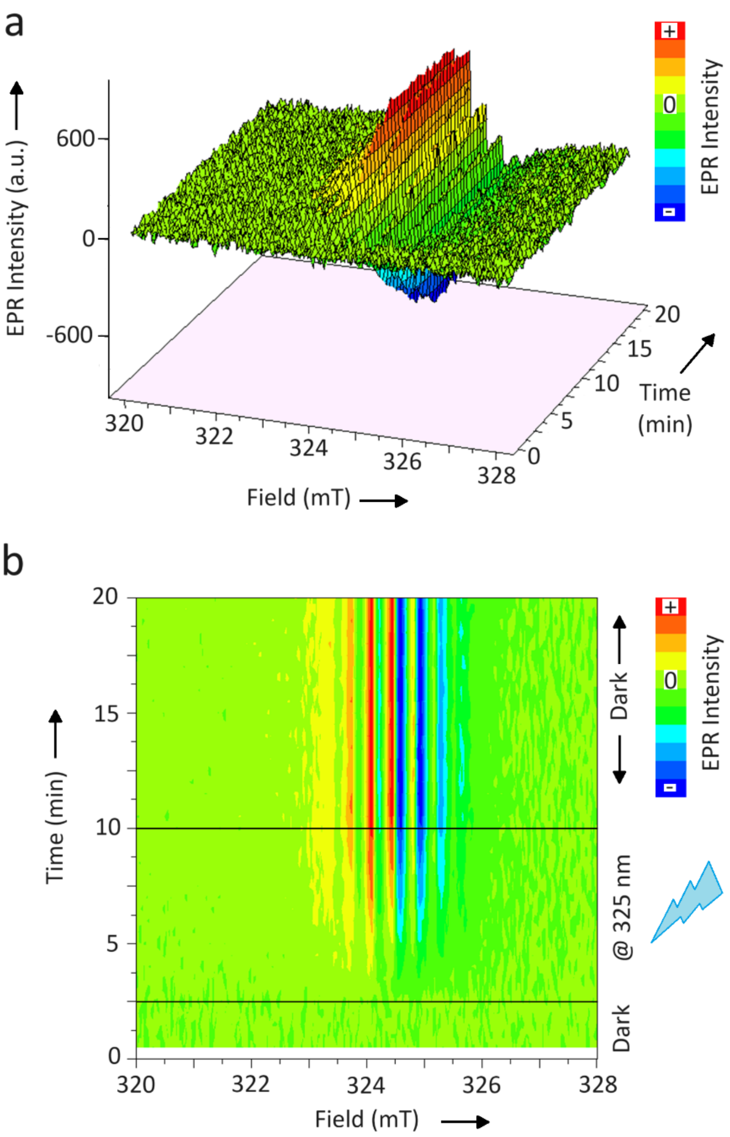

Figure S14. *In situ* light induced X-band EPR spectra (LEPR) of freshly prepared C_1F_1/deuterated acetone (acetone-d_6) (large sheets, commercial source). See also Figure S3. The sample was kept in nitrogen saturated atmosphere. Panel (a) shows the 3D LEPR plot (x, Field (B); y, time (min); z, EPR intensity). Experimental parameters used during signal acquisition: 9.07814 GHz frequency, 0.400 mW microwave power, 100 kHz modulation frequency, 0.05 mT modulation width, 30 s acquisition time for each sequential spectrum, $T = 90$ K. Signal acquisition sequence: 2.5 min under dark conditions, followed by 7.5 min under

continuous in situ light irradiation (@ 325 nm) and then 10 min back to dark conditions. Panel (b) shows the correspondent 2D LEPR plot of the spectra shown in panel (a).

Note: the LEPR spectrum (C_1F_1/deuterated acetone (acetone-d_6), large sheets, commercial source) clearly shows that the photoexcited e^- in FG remains firmly localized onto the fluorographene backbone (“Holstein” type) and do not undergo any diffusion along the carbon framework, which can translate in significant alteration in the total spread of the resonance line along the magnetic field; such phenomenon is in fact not observed to occur here. The EPR features of the h^- counterpart on the acetone d_6 molecules remains hidden due to the deuterium substitution.

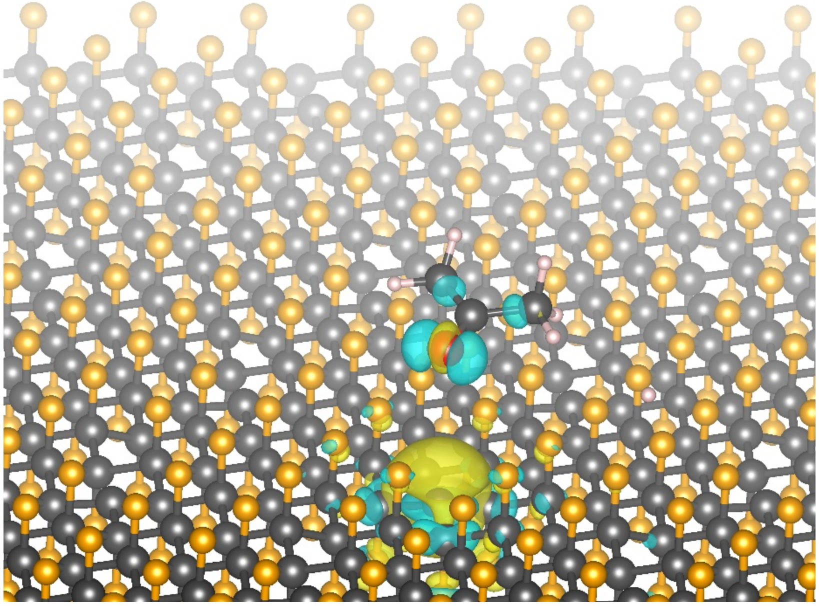

Figure S15. The charge density difference due to the excitation of an electron from acetone to FG radical system. The difference is calculated with respect to the ground-state charge density (yellow – negative charge, cyan - positive charge, the isovalue = 0.0025).

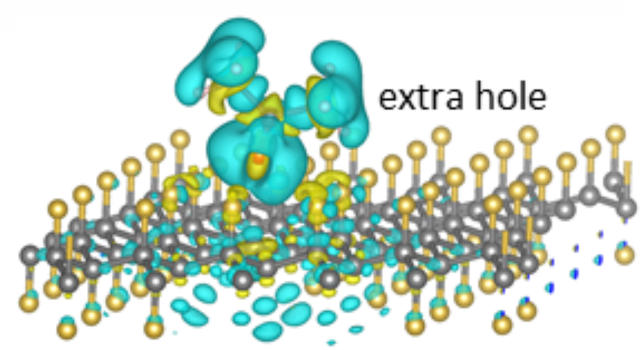

Figure S16. The charge density difference due to an extra hole in FG radical/acetone system. The difference is calculated with respect to the sum of the charge densities of isolated neutral components, the acetone molecule and FG radical (yellow – negative charge, cyan - positive charge, the isovalue = 0.0025).

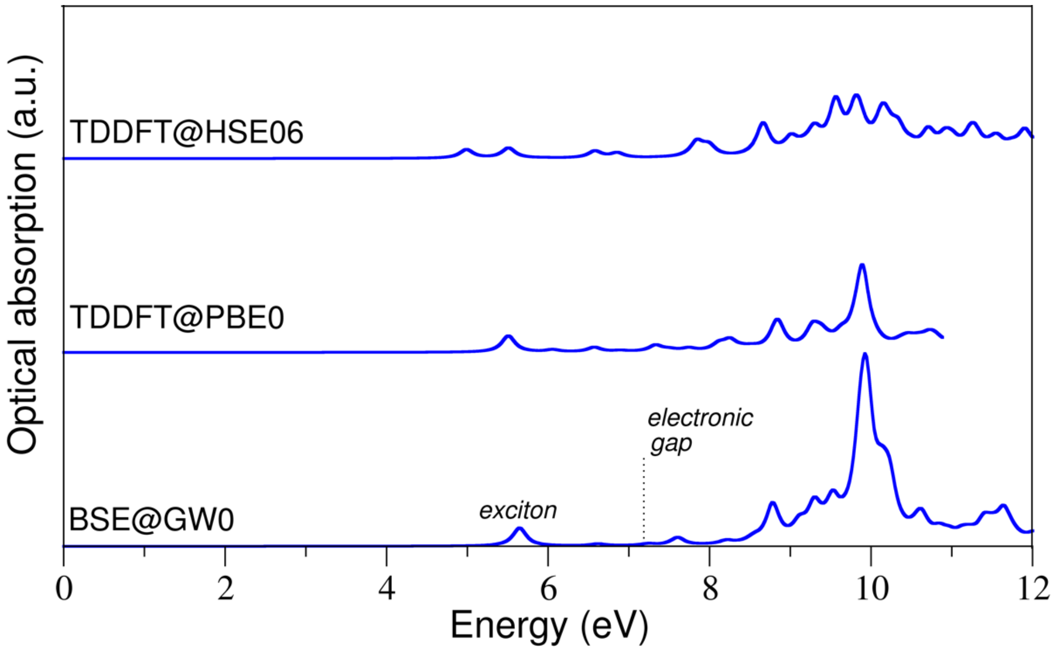

Figure S17. Optical absorption spectrum of pristine FG calculated at the BSE@GW0 level and TD-DFT with the PBE0 and HSE06 hybrid functional as the kernel. See main text for computational details.
